# Supplementary material for: Integration of stepped care for perinatal mood and anxiety disorders among women attending maternal and child health clinics in Kenya: Protocol for a cluster randomized controlled trial
Source: PLoS One. 2026 Jun 11;21(6):e0349732. doi: 10.1371/journal.pone.0349732 (PMC13257984; doi:10.1371/journal.pone.0349732)
Supplement: S2 FIle — (DOCX) [file pone.0349732.s002.docx]

|  | **APPLICATION IRB Protocol** |
| --- | --- |
|  | |
|  | |

The Human Subjects Division (HSD) strives to ensure that people with disabilities have access to all services and content. **If you experience any accessibility-related issues with this form or any aspect of the application process, email** [**hsdinfo@uw.edu**](mailto:hsdinfo@uw.edu) **for assistance.**

INSTRUCTIONS

- **This form is only for studies that will be reviewed by the UW IRB**. Before completing this form, check [HSD’s website](https://www.washington.edu/research/hsd/is-the-uw-irb-the-right-irb/) to confirm that this should not be reviewed by an external (non-UW) IRB.
- **If you are requesting a determination** about whether the planned activity is human subjects research or qualifies for exempt status, you may skip all questions except those marked with **[DETERMINATION]** For example **1.1. [DETERMINATION]** must be answered. Do not upload consent materials for determinations in ***Zipline*** as HSD does not review or approve them.
- **Answer all questions**. If a question is not applicable to the research or if you believe you have already answered a question elsewhere in the application, state “NA” (and if applicable, refer to the question where you provided the information). If you do not answer a question, the IRB does not know whether the question was overlooked or whether it is not applicable. This may result in unnecessary “back and forth” for clarification. Use non-technical language as much as possible.
- For collaborative or multi-site research, describe only the UW activities unless you are requesting that the UW IRB provide the review and oversight for non-UW collaborators or co-investigators as well.
- You may reference other documents (such as a grant application) if they provide the requested information in non-technical language. Be sure to provide the document name, page(s), and specific sections, and upload it to ***Zipline***. Also, describe any changes that may have occurred since the document was written (for example, changes that you’ve made during or after the grant review process). In some cases, you may need to provide additional details in the answer space as well as referencing a document.
- **NOTE: Do not convert this Word document to PDF.** The ability to use “tracked changes” is required in order to modify your study and respond to screening requests

INDEX

**1.** [Overview](#OVERVIEW)

**2.** [Participants](#PARTICIPANTS)

**3.** [Non-UW Research Setting](#NONUWRESEARCHSETTINGS)

**4.** [Recruiting and Screening Participants](#RECRUTINGANDSCREENINGPARTICIPANTS)

**5.** [Procedures](#PROCEDURES)

**6.** [Children (Minors) and Parental Permission](#CHILDRENANDPARENTALPERMISSION)

**7.** [Assent of Children (Minors)](#ASSENTOFCHILDREN)

**8.** [Consent of Adults](#CONSENTOFADULTS)

**9.** [Privacy and Confidentiality](#PRIVACYANDCONFIDENTIALITY)

**10**. [Risk / Benefit Assessment](#RISKBENEFITASSESSMENT)

**11**. [Economic Burden to Participants](#ECONOMICBURDENTOPARTICIPANTS)

**12.** [Resources](#RESOURCES)

**13.** [Other Approvals, Permissions, and Regulatory Issues](#OTHERAPPROVALSPERMISSIONSANDREGULATORYIS)

1. OVERVIEW

| **Integration of stepped care for perinatal mood and anxiety disorders among women attending MCH clinics in Kenya** |
| --- |

**Study Title:**

**1.1. [DETERMINATION]**  **Home institution.** Identify the institution through which the lead researcher listed on the IRB application will conduct the research. Provide any helpful explanatory information.

*In general, the home institution is the institution (1) that provides the researcher’s paycheck and that considers them to be a paid employee, or (2) at which the researcher is a matriculated student. Scholars, faculty, fellows, and students who are visiting the UW and who are the lead researcher: identify your home institution and describe the purpose and duration of your UW visit, as well as the UW department/center with which you are affiliated while at the UW.*

*Note that many UW clinical faculty members are paid employees of non-UW institutions.*

*The UW IRB provides IRB review and oversight for only those researchers who meet the criteria described in the* [***SOP Use of the UW IRB***](https://www.washington.edu/research/policies/sop-use-of-the-uw-irb-2/)*.*

| Kenyatta National Hospital, Nairobi Kenya |
| --- |

**1.2. [DETERMINATION]**  **Consultation history**. Has there been any consultation with someone at HSD about this study?

*It is not necessary to obtain advance consultation. However, if advance consultation was obtained, answering this question will help ensure that the IRB is aware of and considers the advice and guidance provided in that consultation.*

**No**

**Yes →** Briefly describe the consultation: approximate date, with whom, and method (e.g., by email, phone call, in-person meeting).

| Click or tap here to enter text. |
| --- |

**1.3. [DETERMINATION]** **Similar and/or related studies**. Are there any related IRB applications that provide context for the proposed activities?

*Examples of studies for which there is likely to be a related IRB application: Using samples or data collected by another study; recruiting subjects from a registry established by a colleague’s research activity; conducting Phase 2 of a multi-part project or conducting a continuation of another study; serving as the data coordinating center for a multi-site study that includes a UW site.*

*Providing this information (if relevant) may significantly improve the efficiency and consistency of the IRB’s review.*

**No**

**Yes →** Briefly describe the other studies or applications and how they relate to the proposed activities. If the other applications were reviewed by the UW IRB, please also provide: the UW IRB number, the study title, and the lead researcher’s name.

|  |
| --- |

**1.4. [DETERMINATION]**  **Externally-imposed urgency or time deadlines**. Are there any externally-imposed deadlines or urgency that affect the proposed activity?

*HSD recognizes that everyone would like their IRB applications to be reviewed as quickly as possible. To ensure fairness, it is HSD policy to review applications in the order in which they are received. However, HSD will assign a higher priority to research with externally-imposed urgency that is beyond the control of the researcher. Researchers are encouraged to communicate as soon as possible with their HSD staff contact person when there is an urgent situation (in other words, before submitting the IRB application). Examples: a researcher plans to test an experimental vaccine that has just been developed for a newly emerging epidemic; a researcher has an unexpected opportunity to collect data from students when the end of the school year is only four weeks away.*

*HSD may ask for documentation of the externally-imposed urgency. A higher priority should not be requested to compensate for a researcher’s failure to prepare an IRB application in a timely manner. Note that IRB review requires a certain minimum amount of time; without sufficient time, the IRB may not be able to review and approve an application by a deadline.*

**No**

**Yes** **→** Briefly describe the urgency or deadline as well as the reason for it.

| Funding allocation for this study from the NIH is contingent on achieving IRB approval from the Kenyatta National Hospital and University of Washington. We are seeking IRB approval as soon as possible. We have not been provided with a specific due date, but “as soon as possible” is the general guidance from NIH. |
| --- |

**1.5. [DETERMINATION]**  **Objectives**. Using lay language, describe the purpose, specific aims, or objectives that will be met by this specific project. If hypotheses are being tested, describe them. You will be asked to describe the specific procedures in a later section.

If this application involves the use of a HUD “humanitarian” device: describe whether the use is for “on-label” clinical patient care, “off-label” clinical patient care, and/or research (collecting safety and/or effectiveness data).

| We have identified three evidence-based interventions to promote perinatal mental health: universal WHO Mental Health Gap Action Programme (mhGAP)-recommended mental health screening, the Problem Management Plus (PM+) counseling intervention for women experiencing perinatal mood and anxiety disorders (PMAD), and telepsychiatry for women with severe symptoms, suicidality or no response to PM+. We propose to combine these interventions in a stepped care model, named the Integrated Perinatal Mental Health program (IPMH), and develop implementation strategies to support the model’s integration into routine perinatal care in Kenya. We will then evaluate IPMH’s effectiveness and implementation outcomes in a Hybrid Type II trial.  SPECIFIC OBJECTIVES  Aim 1: Using participatory design, optimize and adapt IPMH and develop implementation strategies.  (a) IPMH intervention components will be optimized and adapted, guided by client, provider and policymaker stakeholder input to ensure that IPMH can be implemented in these facilities. We will conduct quantitative surveys, key informant interviews (KIIs) and focus group discussions (FGDs), followed by workshops with stakeholders to co-design the intervention.  (b) Implementation strategies will be developed to address stakeholders’ perceived barriers to implementing IPMH using the Consolidated Framework for Implementation Research (CFIR). We will conduct KIIs and FGDs with providers and policymakers to identify barriers, followed by workshops with stakeholders to co-design strategies to overcome them.  Hypothesis: Implementation barriers include insufficient provider knowledge and skills in counselling techniques, overburdened staff, and inefficient patient flow. We predict that promising implementation strategies will include provider training, structured supervision, multidisciplinary teleconferenced team-based learning, streamlining service delivery points, provision of job aides and manuals and audit and feedback in the first 6 months of implementation.  Aim 2: Determine the effect of IPMH and implementation strategies on mental health, HIV care, and pregnancy outcomes among perinatal women from pregnancy to 6 months postpartum.  We will conduct a 1:1 cluster-randomized trial in 20 facilities in Western Kenya among pregnant women (3rd trimester) with PMAD symptoms (Patient Health Questionnaire-2 [PHQ-2]≥3 or Generalized Anxiety Disorder-7 [GAD-7]≥3). Control facilities will provide enhanced standard of care (PMAD screening followed by standard of care). Intervention facilities will provide IPMH. Effectiveness outcomes will be compared between study arms. Primary outcomes include PMAD symptoms (PHQ-9 score and GAD-7 score) at 6 months postpartum. Secondary outcomes include quality of life and adverse pregnancy outcomes. Exploratory outcomes include HIV viral suppression, MTCT, and HIV-related stigma.  Hypothesis: Perinatal women at facilities randomized to IPMH will have lower depressive and anxiety symptoms, lower adverse pregnancy outcomes, and improved quality of life compared with standard of care.  Aim 3: Determine effect of IPMH and its implementation strategies on service delivery and implementation outcomes, and identify multilevel drivers of successful implementation.  a) Guided by Proctor’s Implementation Outcomes Framework (IOF), we will quantitatively compare penetration, efficiency and equity of screening and care provision. Additionally, acceptability, adoption, fidelity, and cost of IPMH will be described within the intervention arm.  b) Guided by the Consolidated Framework for Implementation Research (CFIR), we will explore intervention-, facility- and provider-level drivers of implementation success, by conducting quantitative and qualitative analysis in facilities with high vs. low penetration, equity, efficiency, acceptability, and fidelity.  Hypothesis: IPMH will increase penetration and equity of care provision to perinatal women experiencing PMAD without negatively impacting efficiency of care delivery. |
| --- |

**1.6. [DETERMINATION]**  **Study design**. Provide a one-sentence description of the general study design and/or type of methodology.

*Your answer will help HSD in assigning applications to reviewers and in managing workload. Examples: a longitudinal observational study; a double-blind, placebo-controlled randomized study; ethnographic interviews; web scraping from a convenience sample of blogs; medical record review; coordinating center for a multi-site study.*

| This is a Hybrid Type II Trial which includes a cluster-randomized controlled trial to evaluate the clinical and implementation science outcomes of IPMH informed by a qualitative participatory design formative phase. |
| --- |

**1.7. [DETERMINATION]**  **Intent**. Check all the descriptors that apply to your study. You must check at least one box.

*This question is essential for ensuring that your application is correctly reviewed. Please read each option carefully.*

| **Check all that apply** | **Descriptor** |
| --- | --- |
|  | Class project or other activity whose purpose is to provide an educational experience for the researcher (for example, to learn about the process or methods of doing research). |
|  | Part of an institution, organization, or program’s own internal operational monitoring. |
|  | Improve the quality of service provided by a specific institution, organization, or program. |
|  | Designed to expand the knowledge base of a scientific discipline or other scholarly field of study, and produce results that:   - Are expected to apply to a larger population beyond the site of data collection or the specific subjects studied, or - Are intended to be used to develop, test, or support theories, principles, and statements of relationships, or to inform policy beyond the study. |
|  | Focus directly on the specific individuals about whom the information or biospecimens are collected through oral history, journalism, biography, or historical scholarship activities, to provide an accurate and evidence-based portrayal of the individuals. |
|  | A quality improvement or program improvement activity conducted to improve the implementation (delivery or quality) of an accepted practice, or to collect data about the implementation of the practice for clinical, practical, or administrative purposes. This does not include the evaluation of the efficacy of different accepted practices, or a comparison of their efficacy. |
|  | Public health surveillance activities conducted, requested, or authorized by a public health authority for the sole purpose of identifying or investigating potential public health signals or timely awareness and priority setting during a situation that threatens public health. |
|  | Preliminary, exploratory or research development activities (such as pilot and feasibility studies, or reliability/validation testing of a questionnaire). |
|  | Expanded access use of a drug or device not yet approved for this purpose. |
|  | Use of a Humanitarian Use Device. |
|  | Other. Explain: |

| Click or tap here to enter text. |
| --- |

**1.8. Background, experience, and preliminary work**. Answer this question **only** if the proposed activity has one or more of the following characteristics. The purpose of this question is to provide the IRB with information that is relevant to its risk/benefit analysis.

- Involves more than minimal risk (physical or non-physical)
- Is a clinical trial, or
- Involves having the subjects use a drug, biological, botanical, nutritional supplement, or medical device.

*“Minimal risk” means that the probability and magnitude of harm or discomfort anticipated in the research are not greater than those ordinarily encountered in daily life or during the performance of routine physical or psychological examinations or tests.*

**1.8.a.** Background. Provide the rationale and the scientific or scholarly background for the proposed activity, based on existing literature (or clinical knowledge). Describe the gaps in current knowledge that the project is intended to address.

*This should be a plain language description. Do not provide scholarly citations. Limit your answer to less than one page or refer to an attached document with background information that is no more than three pages long.*

| Perinatal mood and anxiety disorders (PMAD), defined as depression and anxiety during pregnancy or up to 1 year postpartum, account for substantial morbidity and mortality among birthing people globally. Approximately 10-20% of pregnant and postpartum women experience PMAD, with elevated burden in low- and middle-income countries (LMICs) (>20% vs. 13% in high-income countries). In addition to causing significant disability among perinatal women, PMAD is associated with risks to the infant, including elevated risk of preterm birth, low birth weight and intra uterine growth retardation, pregnancy loss and stillbirth. Kenya, like many LMICs, experiences a severe shortage of specialized mental healthcare workers (HCWs) and poor coverage of screening and treatment for PMAD. It is estimated that over 75% of those who need mental health services do not receive them, and those who do often travel large distances and experience long waits to see specialist providers. The World Health Organization (WHO) and Kenya’s national mental health strategy have prioritized integration of mental health care in primary care settings, with care provision by non-specialist providers, engagement of lay workers, and use of technology to improve intervention reach.  Several evidence-based interventions (EBIs) are recommended by the WHO for identification and management of PMAD by non-specialist providers in resource-limited settings. However, their implementation in routine care in Kenya is currently limited, due to lack of provider training and comfort with perinatal mental health treatments and poor linkage across cadres of providers (such as primary care providers and mental health providers). Sustainable integration of perinatal mental healthcare in Kenya’s routine perinatal services requires development and testing of tailored interventions that strengthen workforce capacity and facilitate linkage across provider cadres, as well as implementation strategies to facilitate high-quality intervention delivery. We have identified three EBIs to promote perinatal mental health: universal WHO Mental Health Gap Action Programme (mhGAP)-recommended mental health screening, the Problem Management Plus (PM+) counseling intervention for women experiencing PMAD, and telepsychiatry for women with severe symptoms, suicidality or no response to PM+. We propose to combine these interventions in a stepped care model, named the Integrated Perinatal Mental Health program (IPMH), and develop implementation strategies to support the model’s integration into routine perinatal care in Kenya. We will then evaluate IPMH’s effectiveness and implementation outcomes in a Hybrid Type II trial. |
| --- |

**1.8.b.** Experience and preliminary work. Briefly describe experience or preliminary work or data (if any) that you, your team, or your collaborators/co-investigators have that supports the feasibility and/or safety of this study.

*It is not necessary to summarize all the discussion that has led to the development of the study protocol. The IRB is interested only in short summaries about experiences or preliminary work that suggest the study is feasible and that risks are reasonable relative to the benefits. Examples: Your team has already conducted a Phase 1 study of an experimental drug which supports the Phase 2 study being proposed in this application; your team has already done a small pilot study showing that the reading skills intervention described in this application is feasible in an after-school program with classroom aides; your team has experience with the type of surgery that is required to implant the study device; the study coordinator is experienced in working with subjects who have significant cognitive impairment.*

| Our approach to integrate mental health services in MCH in Kenya is novel, potentially scalable and aligns with the Kenyan National initiatives and mental health policy goals.  The study has brought together a team of experts that includes reproductive health, mental health, HIV, epidemiologists and implementation scientists highly experienced in the conduct of collaborative research and large clinical trials. The primary investigators have all led similar clinical trials including among pregnant women and including evaluation of mental health outcomes. |
| --- |

**1.8.c.** Subject matter expertise. Is the study a clinical trial and/or does the study involve use of a drug, biologic, botanical, nutritional supplement and/or is the study otherwise considered to be greater than minimal risk to subjects?

**No →** Answering this question is optional.

**Yes →** Provide the name, degree(s), and contact information (e.g., email, phone number) of someone with appropriate expertise in the subject matter described in the objectives and design of this study. The individual should be unaffiliated with the study and have no other apparent conflict of interest. The individual may be associated with the UW or external to the University. Ensure the individual is aware they may be contacted by HSD.

*Provision of this information is* ***required*** *for all clinical trials, for studies involving the use of a drug, biologic, botanical, nutritional supplement and for studies involving greater than minimal risk. For all other studies, the information is optional, though HSD reserves the right to request researcher assistance in providing a consultant if necessary to complete review of the study.*

*For the consultant, the request involves a brief email or phone call with targeted questions that usually can be responded to in 30 minutes or less.*

| Click or tap here to enter text. |
| --- |

**1.9. Supplements**. Check all boxes that apply, to identify relevant SUPPLEMENTS that should be completed and uploaded to ***Zipline***.

*This section is here instead of at the end of the form to reduce the risk of duplicating information in this IRB Protocol form that you will need to provide in these Supplements.*

| **Check all that apply** | **Type of Research** | **Supplement Name and Link** |
| --- | --- | --- |
|  | **Department of Defense**  The research involves Department of Defense funding, facilities, data, or personnel. | [SUPPLEMENT Department of Defense](https://www.washington.edu/research/forms-and-templates/supplement-department-of-defense/) |
|  | **Department of Energy**  The research involves Department of Energy funding, facilities, data, or personnel. | [SUPPLEMENT Department of Energy](https://www.washington.edu/research/forms-and-templates/supplement-department-of-energy/) |
|  | **Drug, biologic, botanical, supplement**  Procedures involve the use of any drug, biologic, botanical or supplement, even if the item is not the focus of the proposed research. | [SUPPLEMENT Drugs](https://www.washington.edu/research/forms-and-templates/supplement-drugs-biologics-botanicals-supplements/) |
|  | **Emergency exception to informed consent**  Research that requires this special consent waiver for research involving more than minimal risk. | [SUPPLEMENT Exception from Informed Consent for Emergency Research (EFIC)](https://www.washington.edu/research/forms-and-templates/supplement-exception-from-informed-consent-for-emergency-research-efic/) |
|  | **Genomic data sharing**  Genomic data are being collected and will be deposited in an external database (such as the NIH dbGaP database) for sharing with other researchers, and the UW is being asked to provide the required certification or to ensure that the consent forms can be certified. | [SUPPLEMENT Genomic Data Sharing](https://www.washington.edu/research/forms-and-templates/supplement-genomic-data-sharing/) |
|  | **Medical device**  Procedures involve the use of any medical device, even if the device is not the focus of the proposed research, except when the device is FDA-approved and is being used through a clinical facility in the manner for which it is approved. | [SUPPLEMENT Devices](https://www.washington.edu/research/forms-and-templates/supplement-devices/) |
|  | **Multi-site or collaborative study**  The UW IRB is being asked to review on behalf of one or more non-UW institutions in a multi-site or collaborative study. | [SUPPLEMENT Multi-site or Collaborative Research](https://www.washington.edu/research/forms-and-templates/supplement-multi-site-or-collaborative-research/) |
|  | **Non-UW Individual Investigators**  The UW IRB is being asked to review on behalf of one or more non-UW individuals who are not affiliated with another organization for the purpose of the research. | [SUPPLEMENT Non-UW Individual Investigators](https://www.washington.edu/research/forms-and-templates/supplement-non-uw-individual-investigators/) |
|  | **Other REDCap Installation Attestation for Electronic Consent**  The research will use a non-UW installation of REDCap for conducting and/or documenting informed consent. | [SUPPLEMENT Other REDCap Installation](https://www.washington.edu/research/forms-and-templates/supplement-other-redcap-installation/) |
|  | None of the above. |  |

2. PARTICIPANTS

**2.1. [DETERMINATION]**  **Participants**. Describe the general characteristics of the subject populations or groups, including age range, gender, health status, and any other relevant characteristics.

| The study will involve perinatal women attending perinatal care at the facilities as well as providers and policy makers in maternal and child health and prevention of vertical HIV transmission. Perinatal women will be ≥14 years of age while providers and policy makers will be ≥18years of age. |
| --- |

**2.2. [DETERMINATION]**  **Inclusion and exclusion criteria.**

**2.2.a. Inclusion criteria**. Describe the specific criteria that will be used to decide who will be included in the research from among interested or potential subjects. Define any technical terms in lay language.

| Aim 1:   - Perinatal women   - ≥14 years   - receiving perinatal care at the study facility   - pregnant or <1 year postpartum. - Healthcare workers   - ≥ 18 years in age   - a nurse, clinical officer, HIV testing services (HTS) provider or lay healthcare worker at study facility, or clinical psychologist or psychiatrist at county or national referral hospital - Policy makers   - age ≥18   - involved in perinatal and/or mental health policy at national or county level   Aim 2 and 3:   - Perinatal women   - pregnant at ≥28 weeks gestation   - age ≥14 years   - screen positive for PMAD symptoms (PHQ-2≥3 and/or GAD-2≥3)   - attending ANC care at the facility   - willing to return to MCH/PMTCT clinics for study visits.   - Participants on antidepressants will be eligible if they have been on a stable dose for ≥6 months. - Healthcare workers   - ≥ 18 years in age   - a nurse, clinical officer, HIV testing services (HTS) provider or lay healthcare worker at study facility, or clinical psychologist or psychiatrist at county or national referral hospital |
| --- |

**2.2.b. Exclusion criteria**. Describe the specific criteria that will be used to decide which of the subjects who meet the inclusion criteria listed above will be excluded from the research. Define any technical terms in lay language.

| Any woman at high risk of self-harm based on a study self-harm assessment protocol, has cognitive impairments, or psychotic symptoms will not be eligible to participate, but will be directly assessed by a supervising clinician who will determine next steps in referral and treatment. |
| --- |

**2.3. [DETERMINATION]**  **Prisoners**. IRB approval is required in order to include prisoners in research, even when prisoners are not an intended target population.

Is the research likely to have subjects who become prisoners while participating in the study?

*For example, a longitudinal study of youth with drug problems is likely to have subjects who will be prisoners at some point during the study.*

**No**

**Yes →** If a subject becomes a prisoner while participating in the study, will any study procedures and/or data collection related to the subject be continued while the subject is a prisoner?

**No**

**Yes →** Describe the procedures and/or data collection that will continue with prisoner subjects.

| Click or tap here to enter text. |
| --- |

**2.4.** **[DETERMINATION]** Will the proposed research recruit or obtain data from individuals that are known to be prisoners?

*For records reviews: if the records do not indicate prisoner status and prisoners are not a target population, select “No”. See the* [*GUIDANCE Prisoners*](https://www.washington.edu/research/hsd/guidance/protected-vulnerable-populations/prisoners/) *for the definition of “prisoner”, which is not necessarily tied to the type of facility in which a person is residing.*

**No**

**Yes →** Answer the following questions (**2.4.a.** – **2.4.d.)**

**2.4.a.** Describe the type of prisoners, and their locations(s).

| Click or tap here to enter text. |
| --- |

**2.4.b.** One concern about prisoner research is whether the effect of participation on prisoners’ general living conditions, medical care, quality of food, amenities, and/or opportunity for earnings in prison will be so great that it will make it difficult for prisoners to adequately consider the research risks. How will the chances of this be reduced?

| Click or tap here to enter text. |
| --- |

**2.4.c.** Describe what will be done to make sure that (a) recruitment and subject selection procedures will be fair to all eligible prisoners and (b) prison authorities or other prisoners will not be able to arbitrarily prevent or require particular prisoners from participating.

| Click or tap here to enter text. |
| --- |

**2.4.d.** If the research is funded by one of these federal departments and agencies (Health & Human Services; Energy; Defense; Homeland Security; CIA; Social Security Administration), and/or will involve prisoners in federal facilities or in state/local facilities outside of Washington State: check the box below to provide assurance that study team members will (a) not encourage or facilitate the use of a prisoner’s participation in the research to influence parole or pardon decisions, and (b) clearly inform each prisoner in advance (for example, in a consent form) that participation in the research will have no effect on his or her parole or pardon.

**Confirmed**

**2.5. [DETERMINATION]**  **Protected populations.** IRB approval is required for the use of the subject populations listed here. Check the boxes for any of these populations that will be purposefully included. (In other words, being a part of the populations is an inclusion criterion for the study.)

*The WORKSHEETS describe the criteria for approval but do not need to be completed and should not be submitted.*

| **Check all that apply** | **Population** | **Worksheet Name and Link** |
| --- | --- | --- |
|  | Fetuses in utero | [WORKSHEET Pregnant Women](https://www.washington.edu/research/forms-and-templates/worksheet-pregnant-women/) |
|  | Neonates of uncertain viability | [WORKSHEET Neonates](https://www.washington.edu/research/forms-and-templates/worksheet-neonates/) |
|  | Non-viable neonates | [WORKSHEET Neonates](https://www.washington.edu/research/forms-and-templates/worksheet-neonates/) |
|  | Pregnant women | [WORKSHEET Pregnant Women](https://www.washington.edu/research/forms-and-templates/worksheet-pregnant-women/) |

**2.5.a.** If you check any of the boxes above, use this space to provide any information that may be relevant for the IRB to consider.

| The proposed study will evaluate integration of stepped care for perinatal mood and anxiety disorders among perinatal women. |
| --- |

**2.6. [DETERMINATION]**  **Native Americans or non-U.S. indigenous populations**. Will Native American or non-U.S. indigenous populations be actively recruited through a tribe, tribe-focused organization, or similar community-based organization?

*Indigenous people are defined in international or national legislation as having a set of specific rights based on their historical ties to a particular territory and their cultural or historical distinctiveness from other populations that are often politically dominant.*

*Examples: a reservation school or health clinic; recruiting during a tribal community gathering.*

**No**

**Yes →** Name the tribe, tribal-focused organization, or similar community-based organization. The UW IRB expects that tribal/indigenous approval will be obtained before beginning the research. This may or may not involve approval from a tribal IRB. The study team and any collaborators/investigators are also responsible for identifying any tribal laws that may affect the research.

| Click or tap here to enter text. |
| --- |

**2.7. [DETERMINATION]**  **UW Medicine and UW Dentistry residents and fellows.** Will the research involve UW Medicine or UW Dentistry residents or fellows as study subjects?

**If it will → (1)** Describe in the Recruiting section ([4.1](#RECRUTINGANDSCREENINGPARTICIPANTS)) and Risks section ([10.1](#RISKBENEFITASSESSMENT)) how you will ensure that residents and fellows feel free to truly make a voluntary decision about participation (i.e., no negative consequences from supervisors for saying “no”) and how you will ensure that any research data will not be used in the residents’ and fellows’ supervisor or program evaluation of them; **AND**

**(2)** You must inform the UW HR Labor Relations representative who negotiates with the resident’s and fellows’ union about the study before beginning it. This is currently Jennifer Mallahan [mallaj@uw.edu](mailto:mallaj@uw.edu) .

**2.8. [DETERMINATION]**  **Third party subjects.** Will the research collect private identifiable information about individuals *other than* the study subjects? Common examples include: collecting medical history information or contact information about family members, friends, co-workers.

*“Identifiable” means any direct or indirect identifier that, alone or in combination, would allow you or another member of the research team to readily identify the person. For example, suppose that the research is about immigration history. If subjects are asked questions about their grandparents but are not asked for names or other information that would allow easy identification of the grandparents, then private identifiable information is not being collected about the grandparents and the grandparents are not subjects.*

**No**

**Yes →** These individuals are considered human subjects in the study. Describe them and what data will be collected about them.

| Click or tap here to enter text. |
| --- |

**2.9. Number of subjects**. Is it possible to predict or describe the maximum number of subjects (or subject units) needed to complete the study, for each subject group?

*Subject units mean units within a group. For most research studies, a group will consist of individuals. However, the unit of interest in some research is not the individual. Examples:*

- *Dyads such as caregiver-and-Alzheimer’s patient, or parent and child*
- *Families*
- *Other units, such as student-parent-teacher*

*Subject group means categories of subjects that are meaningful for the specific study. Some research has only one subject group – for example, all UW students taking Introductory Psychology. Some common ways in which subjects are grouped include:*

- *By intervention – for example, an intervention group, and a control group.*
- *By subject population or setting – for example, urban versus rural families*
- *By age – for example, children who are 6, 10, or 14 years old.*

*The IRB reviews the number of subjects in the context of risks and benefits. Unless otherwise specified, if the IRB determines that the research involves no more than minimal risk: there are no restrictions on the total number of subjects that may be enrolled. If the research involves more than minimal risk: The number of enrolled subjects must be limited to the number described in this application. If it is necessary later to increase the number of subjects, submit a Modification. Exceeding the IRB-approved number (*[*over-enrollment*](https://www.washington.edu/research/glossary/over-enrollment/)*) will be considered non-compliance.*

**No →** Provide the rationale in the box below. Also, provide any other available information about the scope/size of the research. You do not need to complete the table.

*Example: It may not be possible to predict the number of subjects who will complete an online survey advertised through Craigslist, but you can state that the survey will be posted for two weeks and the number who respond is the number who will be in the study.*

| Click or tap here to enter text. |
| --- |

**Yes →** For each subject group, use the table below to provide the estimate of the maximum desired number of individuals (or other subject unit, such as families) who will complete the research.

| **Group name/description** | **Maximum desired number or individuals (or other subject unit) who will complete the research**  ***Provide numbers for the site(s) reviewed by the UW IRB and for the study-wide total number; example: 20/100*** |
| --- | --- |
| 1. healthcare workers and perinatal clients in formative data collection (Aim 1) | 630 |
| 2) perinatal clients in the longitudinal RCT cohort (Aim 2) | 560 |
| 1. healthcare workers providing care in the RCT (Aim 3) | 200 |
|  | Click or tap here to enter text. |
|  | Click or tap here to enter text. |
|  | Click or tap here to enter text. |

3. NON-UW RESEARCH SETTINGS

*Complete this section only if UW investigators and people named in the* [*SUPPLEMENT Non-UW Individual Investigators*](https://www.washington.edu/research/forms-and-templates/supplement-non-uw-individual-investigators) *will conduct research procedures outside of UW and Harborview*

**3.1. [DETERMINATION]**  **Research locations and rationale**. Identify the locations where the research will be conducted and include a description of the reason(s) for choosing the locations. If the research will be conducted internationally, be sure to list all the countries where the research will take place.

*This is especially important when the research will occur in locations or with populations that may be vulnerable to exploitation. One of the three ethical principles the IRB must consider is Justice: ensuring that reasonable, non-exploitative, and well-considered procedures are administered fairly, with a fair distribution of costs and potential benefits.*

| The study will be conducted in 20 health care facilities in Kisumu, Siaya, and Homa Bay counties of Western Kenya. Facilities will be selected based on HIV sero-prevalence (15-20%) and ANC volume (≥20 new ANC clients per month) and will include a diversity of facility characteristics such as rural/urban location, mental healthcare model, and facility level. |
| --- |

**3.2. [DETERMINATION]**  **Local context**. Culturally appropriate procedures and an understanding of local context are an important part of protecting subjects. Describe any site-specific cultural issues, customs, beliefs, or values that may affect the research, how it is conducted, or how consent is obtained or documented.

*Examples: It would be culturally inappropriate in some international settings for a woman to be directly contacted by a male researcher; instead, the researcher may need to ask a male family member for permission before the woman can be approached. It may be appropriate to obtain permission from community leaders prior to obtaining consent from individual members of a group. In some distinct cultural groups, signing forms may not be the norm.*

*This federal site maintains an international list of human research standards and requirements:* [*http://www.hhs.gov/ohrp/international/index.html*](http://www.hhs.gov/ohrp/international/index.html)

| English, Kiswahili and Dholuo will be used to obtain consents and for study questionnaires so as to be responsive to the language needs of the community. Back-translations will be done to ensure that everything in the study is expressed in a culturally appropriate manner. |
| --- |

**3.3. [DETERMINATION]**  **Location-specific laws.** Describe any local laws that may affect the research (especially the research design and consent procedures). The most common examples are laws about:

- **Specimens** – for example, some countries will not allow biospecimens to be taken out of the country.
- **Age of consent** – laws about when an individual is considered old enough to be able to provide consent vary across states, and countries.
- **Legally authorized representative** – laws about who can serve as a legally authorized representative (and who has priority when more than one person is available) vary across states and countries.
- **Use of healthcare records** – many states have laws that are similar to the federal HIPAA law but that have additional requirements.

| In Kenya pregnant women below 18 years of age are considered emancipated minors and can provide consent to participate in research without parental consent. |
| --- |

**3.4. [DETERMINATION]**  **Location specific administrative or ethical requirements**. Describe local administrative or ethical requirements that affect the research.

*Example: A school district may require researchers to obtain permission from the head district office as well as school principals before approaching teachers or students; a factory in China may allow researchers to interview factory workers but not allow the workers to be paid for their participation.*

| We have applied for ethical approval at the study’s primary institution, Kenyatta National Hospital. Once ethical clearance is done, county government approvals will be sought prior to conducting the study. |
| --- |

**3.5. [DETERMINATION]**  **If the PI is a student: Does the research involve traveling outside of the U.S.?**

**NA**

**No**

**Yes →** Confirm by checking the box that (1) you will register with the [**UW Office of Global Affairs**](http://www.washington.edu/globalaffairs/) before traveling; (2) you will notify your advisor when the registration is complete; and (3) you will request a UW Travel Waiver if the research involves travel to the [list of countries](http://www.washington.edu/globalaffairs/global-travelers/warnings-waivers/#myanchor) requiring a UW Travel Waiver.

**Confirmed**

4. RECRUITING AND SCREENING PARTICIPANTS

**4.1. [DETERMINATION]**  **Recruiting and screening**. Describe how subjects will be identified, recruited, and screened. Include information about: how, when, where, and in what setting. Identify who (by position or role, not name) will approach and recruit subjects, and who will screen them for eligibility.

*Note: Per UW Medicine policy, the UW Medicine eCare/MyChart system may not be used for research recruitment purposes. Additionally, researchers may not use UW Medicine’s Epic Care Everywhere data for research purposes unless the clinical data is necessary for patient/participant safety activities. This means Care Everywhere data cannot be used for recruitment, data abstraction, or any research activities other than those necessary for patient/participant safety.*

| Aim 1:  Potential perinatal client and healthcare worker participants will be approached by study staff and referred to the study research assistant at each clinic to obtain additional information and provide informed consent if interested. The study team will provide information about the study and facility staff will be encouraged to contact the study if they are interested. Policymakers will be individually recruited based on existing contact with the study team, email contacts from government directory searches, or network referral.  Aim 2:  Pregnant women seeking ANC at each of the 20 RCT facilities will be recruited for study participation through in-person outreach by the study team at the facilities. Potential participants will be approached by MCH clinic staff and referred to the study research assistant at each clinic to obtain additional information and provide informed consent if interested. Study staff will assess eligibility with a tablet-based screening questionnaire. Any woman at high risk of self-harm based on a study self-harm assessment protocol, with cognitive impairments, or psychotic symptoms will not be eligible to participate, but will be directly assessed by a supervising clinician who will determine next steps in referral and treatment. All perinatal women will be screened for PMAD symptoms by lay workers, using the PHQ-2 and GAD-2. Positive screeners (PHQ-2≥3 and/or GAD-2≥3) will be eligible for RCT enrollment.  Aim 3: Perinatal women, HTS providers, lay workers, facility nurses, and psychology/psychiatry specialists at study facilities will be recruited by study staff as in Aim 1. |
| --- |

**4.2. Recruitment materials.**

**4.2.a.** What materials (if any) will be used to recruit and screen subjects?

*Examples: talking points for phone or in-person conversations; video or audio presentations; websites; social media messages; written materials such as letters, flyers for posting, brochures, or printed advertisements; questionnaires filled out by potential subjects.*

| Talking points will be used by study staff for in-person recruitment. Tablet-based questionnaires will be used for screening. |
| --- |

**4.2.b.** Upload descriptions of each type of material (or the materials themselves) to ***Zipline***. If letters or emails will be sent to any subjects, these should include a statement about how the subject’s name and contact information were obtained. No sensitive information about the person (such as a diagnosis of a medical condition) should be included in the letter. The text of these letters and emails must be uploaded to ***Zipline*** (i.e., a description will not suffice).

*HSD encourages researchers to consider uploading descriptions of most recruitment and screening materials instead of the materials themselves. The goal is to provide the researchers with the flexibility to change some information on the materials without submitting a Modification for IRB approval of the changes. Examples:*

- *Provide a list of talking points that will be used for phone or in-person conversations instead of a script.*
- *For the description of a flyer, include the information that it will provide the study phone number and the name of a study contact person (without providing the actual phone number or name). This means that a Modification would not be necessary if/when the study phone number or contact person changes. Also, instead of listing the inclusion/exclusion criteria, the description below might state that the flyer will list one or a few of the major inclusion/exclusion criteria.*
- *For the description of a video or a website, include a description of the possible visual elements and a list of the content (e.g., study phone number; study contact person; top three inclusion/exclusion criteria; payment of $50; study name; UW researcher).*

**4.3. [DETERMINATION]**  **Relationship with participant population**. Do any members of the study team have an existing relationship with the study population(s)?

*Example: a study team member may have a dual role with the study population such as being their clinical care provider, teacher, laboratory directory or tribal leader in addition to recruiting them for their research.*

**No**

**Yes →** Describe the nature of the relationship.

|  |
| --- |

**4.4. Payment to participants**. The IRB must evaluate subject payment for the possibility that it will unduly influence subjects to participate. Refer to [**GUIDANCE Subject Payment**](https://www.washington.edu/research/hsd/guidance/subject-payment/) when designing subject payment plans. Provide the following information about your plans for paying research subjects in the text box below or note that the information can be found in the consent form.

- The total amount/value of the payment
- Schedule/timing of the payment [i.e., when will subjects receive the payment(s)]
- Purpose of the payment [e.g., reimbursement, compensation, incentive]
- Whether payment will be “pro-rated” so that participants who are unable to complete the research may still receive some part of the payment

*The IRB expects the consent process or study information provided to the subjects to include all of the above-listed information about payment, including the number and amount of payments, and especially when subjects can expect to receive payment. One of the most frequent complaints received by HSD is from subjects who expected to receive cash or a check on the day that they completed a study and who were angry or disappointed when payment took 6-8 weeks to reach them.*

*Researchers should review current UW Financial Management requirements about when Social Security Numbers must be collected, and when research payment must be reported to the UW Tax Office and the IRS:* [*https://finance.uw.edu/ps/how-pay/research-subjects*](https://finance.uw.edu/ps/how-pay/research-subjects)*.*

*If your study involves the use of Amazon’s Mechanical Turk (MTurk), you must comply with the* [*UW Procurement Services policy*](https://finance.uw.edu/ps/amt/policy) *that no UW employee, family member, or student directly involved in the research will participate as a subject. The policy requires adding a qualifying question that asks whether the subject is a UW employee or family member, or UW student who is directly involved in the research. If they answer yes, they must be disqualified from MTurk activities.*

| Women will receive KSH. 500 (~$5) at each clinical visit for transportation costs and effort at each study visit.  Women will receive KSH. 1000 (~$5) for the focused group discussions for transportation costs, time and effort.  These compensation amounts are consistent with other ongoing studies in this context and have been determined to be appropriate and not coercive. |
| --- |

**4.5. [DETERMINATION]**  **Non-monetary compensation**. Describe any non-monetary compensation that will be provided. Example; extra credit for students; a toy for a child.

| None |
| --- |

**4.5.a.** If class credit will be offered to students, there must be an alternate way for the students to earn the extra credit without participating in the research. If class credit will be offered, describe the alternative non-research method by which students can earn that same course credit, including who will provide the alternative (e.g., a student subject pool; the course instructor).

| NA |
| --- |

**4.6. [DETERMINATION]**  **Will data or specimens be accessed or obtained for recruiting and screening procedures prior to enrollment?**

*Examples: names and contact information; the information gathered from records that were screened; results of screening questionnaires or screening blood tests; Protected Health Information (PHI) from screening medical records to identify possible subjects.*

**No →** Skip the rest of this section; go to [question **5.1.**](#QFiveDotOne)

**Yes →** Describe the data and/or specimens (including PHI) and whether it will be retained as part of the study data.

| Click or tap here to enter text. |
| --- |

**4.7. Consent for recruiting and screening.** Will consent be obtained for any of the recruiting and screening procedures? (Section [8: Consent of Adults](#CONSENTOFADULTS) asks about consent for the main study procedures).

*“Consent” includes: consent from individuals for their own participation; parental permission; assent from children; consent from a legally authorized representative for adult individuals who are unable to provide consent.*

*Examples:*

- *For a study in which names and contact information will be obtained from a registry: the registry should have consent from the registry participants to release their names and contact information to researchers.*
- *For a study in which possible subjects are identified by screening records: there will be no consent process.*
- *For a study in which individuals respond to an announcement and call into a study phone line: the study team person talking to the individual may obtain non-written consent to ask eligibility questions over the phone.*

**No →** Skip the rest of this section; go to [question **5.1.**](#QFiveDotOne)

**Yes →** Describe the consent process.

| Pregnant women seeking ANC at each of the facilities will be recruited for study participation through in-person outreach by the study team at the facilities. Potential participants will be approached by MCH clinic staff and referred to the study research assistant at each clinic to obtain additional information and provide verbal consent for screening. Study staff will assess eligibility with a tablet-based screening questionnaire. |
| --- |

**4.7.a.** Documentation of consent. Will a written or verifiable electronic signature from the subject on a consent form be used to document consent for the recruiting and screening procedures?

**No →** Describe the information that will be provided during the consent process and for which procedures.

| Participants will be informed about the types of questions that will be asked in the screening procedures and given a general overview of the study. Participants will be informed that they can decline to answer any questions and this won’t impact the care or treatment they receive at the facility. |
| --- |

**Yes, written** **→** If yes and a written signature will be used to document consent:

- Upload the consent form to ***Zipline***.

**Yes, electronic →** If yes and an electronic signature will be used to document consent:

- Upload the consent form to ***Zipline***.
- **If the eSignature process or method for recruiting and screening is different than for the main study procedures**, use the questions about electronic consent in Sections 8.3. and 8.4. to differentiate between recruiting/screening and main study electronic consent. **If electronic consent will be used for recruiting/screening but not main study consent**, use 8.3. and 8.4. to describe e-consent and note that it is only for recruiting/screening.

5. PROCEDURES

**5.1. [DETERMINATION]**  **Study procedures**. Using lay language, provide a complete description of the study procedures, including the sequence, intervention, or manipulation (if any), drug dosing information (if any), blood volumes and frequency of draws (if any), use of records, time required, and setting/location. If it is available: Upload a study flow sheet or table to ***Zipline***.

*For studies comparing standards of care: It is important to accurately identify the research procedures. Review the section titled, “When to describe risks for studies evaluating medically recognized standards of care” in the* [***GUIDANCE Consent***](https://www.washington.edu/research/hsd/guidance/consent/#r) *and the draft guidance from the federal Office of Human Research Protections, “*[***Guidance on Disclosing Reasonably Foreseeable Risks in Research Evaluating Standards of Care***](http://www.hhs.gov/ohrp/newsroom/rfc/comstdofcare.html)*”; October 20, 2014.*

*Information about pediatric blood volume and frequency of draws that would qualify for expedited review can be found in this* [**reference table**](http://seattlechildrenslab.testcatalog.org/catalogs/185/files/4069) *on the Seattle Children’s IRB website.*

| Aim 1:  We will conduct cross-sectional surveys among health care workers and perinatal women to characterize current mental health services, barriers and facilitators among a broad sample of participants. We aim for a statistically representative sample of participants for this quantitative survey; based on past studies conducted by this team, we expect low refusal rates. We will then conduct individual semi-structured key informant interviews (KIIs) with purposively selected policymakers and FGDs with purposively selected HCWs and perinatal women for in-depth exploration. Each FGD will include 6-12 participants, large enough to stimulate discussion but not so large to hinder participation. FGDs and KIIs will be facilitated in participants’ preferred language(s) by a Kenyan qualitative interviewer fluent in English, Swahili and Luo, the most common languages in the area. FGDs and KIIs will be audio-recorded, transcribed verbatim, and translated into English. Discussion guides will first enumerate experienced barriers and facilitators to mental healthcare implementation, and key design questions to optimize IPMH. Then they will elicit and prioritize implementation strategies drawing from the CFIR and published literature on barriers and implementation strategies.  Aim 2:  In intervention sites, after screening by PHQ-2 and GAD-2 by HTS counselors, participants will be referred to nurses to administer additional screening by PHQ-9 and GAD-7. Those with likely depression (PHQ-9≥10) or anxiety (GAD-7≥10) will be offered PM+, delivered weekly by a lay provider, under the supervision of MCH nurses. Women with severe depressive symptoms (PHQ-9≥15), those who endorse suicidality, or non-responders to PM+ will be provided mental healthcare via in-facility tele-linkage to a psychiatrist based at a regional or national referral hospital. Nonresponse is defined as <50% reduction or <5-point decrease in PHQ-9 or GAD-7 score after conclusion of PM+ sessions, ascertained by a MCH nurse. Regardless of PHQ-9 score, those who endorse PHQ-9 item 9 (suicidal ideation) will be assessed further using our study self-harm protocol and those considered high risk will be withdrawn from the study and assessed by a supervising clinician. Those deemed appropriate to continue in the study will receive in-facility tele-linkage to a psychiatrist. The psychiatrist will make recommendations regarding medication management prescribed by facility provider or referral to a psychologist for tele-linked psychotherapy for 10-12 cognitive behavioral therapy sessions.  Enhanced standard of care for control sites: eSOC includes two enhancements: 1-HIV testing services providers and lay workers will conduct screening for PMAD symptoms (PHQ-2 and GAD-2). 2-The study team will provide a PMAD referral information sheet to all facilities describing inpatient and outpatient psychiatry services at the nearby referral hospitals (JOORTH and Kisumu County Hospital).  Aim 3:  Perinatal women will participate in questionnaires at each study visit and study close. Healthcare providers will participate in questionnaires at study launch, 6 months and 12 months after study launch. No active efforts will be made to retain providers in their professional positions. However, telephone numbers will be collected for providers at enrollment. If a provider misses a study visit but is confirmed to still be in their professional role, study staff will trace the provider by phone. |
| --- |

**5.2. [DETERMINATION]** **Recordings**. Does the research involve creating audio or video recordings?

**No →** Go to [question **5.3.**](#QFiveDotThree)

**Yes →** Verify that you have described what will be recorded in the answer to [question **5.1**](#QFiveDotOne)**.**, and answer question the question below**.**

**5.2.a.** Before recording, will consent for being recorded be obtained from subjects and any other individuals who may be recorded?

**No →** Email [hsdinfo@uw.edu](mailto:hsdinfo@uw.edu) before submitting this application in ***Zipline***. In the email, include a brief description of the research and a note that individuals will be recorded without their advance consent.

**Yes**

**5.3. [DETERMINATION]**  **MRI scans**. Will any subjects have a Magnetic Resonance Imaging (MRI) scan as part of the study procedures?

*This means scans that are performed solely for research purposes or clinical scans that are modified for research purposes (for example, using a gadolinium-based contrast agent when it is not required for clinical reasons).*

**No →** Go to [question **5.4.**](#QFiveDotFour)

**Yes →** Answer questions **5.3.a** through **5.3.c.**

**5.3.a. Describe the MRI scan(s)**. Specifically:

- What is the purpose of the scan(s)? *Examples: obtain research data; safety assessment associated with a research procedure.*
- Which subjects will receive an MRI scan?
- Describe the minimum and maximum number of scans per subject, and over what time period the scans will occur. *For example: all subjects will undergo two MRI scans, six months apart.*

| Click or tap here to enter text. |
| --- |

**5.3.b. MRI facility**. At which facility(ies) will the MRI scans occur? Check all that apply.

UWMC Radiology/Imaging Services (the UWMC clinical facility)

DISC Diagnostic Imaging Sciences Center (UWMC research facility)

CHN Center for Human Neuroscience MRI Center (Arts & Sciences research facility)

BMIC Biomolecular Imaging Center (South Lake Union research facility)

Harborview Radiology/Imaging Services (the Harborview clinical facility)

Northwest Diagnostic Imaging

Other: identify in the text box below:

| Click or tap here to enter text. |
| --- |

**5.3.c. Personnel**. For MRI scans that will be conducted at the DISC, CHN or BMIC research facilities: Indicate who will be responsible for operating the MRI scanner by checking all that apply.

MRI technician who is formally qualified

Researcher who has completed scanner operator training provided by a qualified MRI operator

**5.4. [DETERMINATION]**  **Data variables**. Describe the specific data that will be obtained (including a description of the most sensitive items). Alternatively, a list of the data variables may be uploaded to ***Zipline***.

| We have uploaded a description of variables to Zipline. |
| --- |

**5.5. [DETERMINATION]**  **Data sources**. For all types of data that will be accessed or collected for this research: Identify whether the data are being obtained from the subjects (or subjects’ specimens) or whether they are being obtained from some other source (and identify the source).

*If you have already provided this information in* [*Question 5.1*](#QFiveDotOne)*, you do not need to repeat the information here.*

| Aim 1:  We will engage several stakeholder groups: perinatal women, specialist and non-specialist HCWs (HTS and lay providers), and policymakers working in perinatal and mental health at the national and county levels.  Data sources will include quantitative surveys followed by qualitative key informant interview (KIIs) and focused group discussions (FGDs).  A subset of 12 participants from FGDs and KIIs across all stakeholder groups will be invited to join a participatory design group (PDG). This will be a compensated group of policymakers, HCWs, HTS providers, lay providers and perinatal women who will be invited to participate in a series of 4-6 workshops aimed at analyzing the summary of survey, KII and FGD data and adapting IPMH and defining its implementation strategies.  Aim 2:  Study visits data collection will be conducted by study staff using tablet-based questionnaires at enrollment (pregnancy), 6 weeks, 14 weeks, and 6 months postpartum among participants in both study arms to align with routine well-baby visits. Questionnaires will be used to record self-reported outcomes and sociodemographic and clinical characteristics that may be associated with them, such as maternal education, household income, employment, parity, distance from home to clinic, intimate partner violence, food insecurity, prior healthcare experiences, and infant sex. The exit visit questionnaire will also evaluate participants’ experiences and satisfaction with the care received. Data will also be abstracted from patient facility records and the MCH booklet for pregnancy outcomes. Participants who are 2 weeks late for a study visit will be actively traced by phone call and/or home visit to optimize completeness of visit data. This approach has been highly acceptable in our other cohorts of peripartum women in Western Kenya and has resulted in excellent retention.  Aim 3:  Service delivery and implementation outcomes will be determined from several data sources:  RCT questionnaires: Self-report questionnaires will be administered to RCT participants at study visits to ascertain IPMH acceptability, receipt of screening, linkage to care, and PM+ components received. They will be administered to RCT participants at study visits (see Aim 2). These will ascertain receipt of screening by PHQ-9 and linkage to care for depression or anxiety, including HTS/ lay HCW delivered PM+, specialist-delivered psychiatric care, or antidepressant description. Participants reporting receipt of IPMH will be asked which topics they discussed. Additionally, participants’ RCT exit questionnaire will include standardized questions assessing IPMH acceptability  Checklists: We will assess HCW competence directly after training using a self-administered checklist.  Routine program data: We will abstract anonymous patient count data on a number of MCH and PMTCT appointments completed per month from registers at all facilities.  RCT HCW questionnaires: All HCWs will complete a questionnaire at RCT launch, 6 months and 12 months after launch to assess IPMH acceptability and HCW time use.  Time and motion study and direct observation of patient flow: This will be conducted to determine the time and resources necessary to provide IPMH compared to eSOC. Time and motion studies will be conducted over a two-week period at each site at RCT launch and 12 months later. Trained research assistants will conduct direct observation of patient flow (physical walk throughs to graphically represent patient flow pathways) and time-and-motion data collection (counting minutes spent waiting and receiving services).  Costing: Measures of costs will include (a) clinical and health system costs and (b) participant cost. We will use a combination of methods for collecting data using project expenditures and micro-costing methods to estimate the incremental economic costs in each arm in a subset of 5-10 facilities, purposively selected to represent heterogeneity in drivers of cost. We will collect site-specific inputs for labor and non-labor costs related to planning, training, facilitation administration, and IPMH service delivery. For perinatal women, we will estimate the value of their time as well as costs incurred for childcare and lost wages. We will estimate start-up and recurrent costs for all activities.Where available, we will obtain expenditure data directly from project records or facility financial records to ensure that we capture all costs associated with planning, program design, adaptation, materials development, training, and clinical service delivery. For resource use not captured in project expense reports, we will conduct interviews with IPMH providers, perinatal women, and administrators on the resource use for each intervention component and implementation strategy, capturing key information on quantities and prices each activity, as possible. At the facility level, we will collect site-specific inputs for labor (personnel time, volunteer labor) and non-labor costs (commodities, capital goods (e.g., vehicles, computers, cell phones, other equipment, overhead) related to planning, training, facilitation administration, and IPMH service delivery. All goods and services will be valued regardless of if they were paid for by the project or donated. For perinatal women, we will estimate the value of their time traveling to and from facilities and receiving care services, as well as costs incurred for childcare and lost wages. To estimate a total incremental cost and unit cost, we will estimate the cost of IPMH plus implementation strategies compared to eSOC in control facilities, along with the number of participants receiving care in each arm. We will conduct costing activities in a subset of 5-10 facilities purposively selected to represent heterogeneity in drivers of cost (e.g., patient volume, staffing, location, prevalence of PMAD symptoms).  RCT for perinatal women & HCW questionnaires: We will administer standardized questionnaires to perinatal women and HCW during the RCT assessing determinants of implementation success quantitatively.  Qualitative interviews with perinatal women and HCWs: IDIs, FGDs and KIIs will be conducted among perinatal women and HCWs in intervention facilities to explore drivers of implementation success. |
| --- |

**5.6. [DETERMINATION]**  **Identifiability of data and specimens.** Answer these questions carefully and completely. This will allow HSD to accurately determine the type of review that is required and the relevant compliance requirements. Review the following definitions before answering the questions:

***Access*** *means to view or perceive data, but not to possess or record it. See, in contrast, the definition of “obtain”.*

***Identifiable*** *means that the identity of an individual is or may be readily (1) ascertained by the researcher or any other member of the study team from specific data variables or from a combination of data variables, or (2) associated with the information.*

***Direct identifiers*** *are direct links between a subject and data/specimens. Examples include (but are not limited to): name, date of birth, medical record number, email or IP address, pathology or surgery accession number, student number, or a collection of data that is (when taken together) identifiable.*

***Indirect identifiers*** *are information that links between direct identifiers and data/specimens. Examples: a subject code or pseudonym.*

***Key*** *refers to a single place where direct identifiers and indirect identifiers are linked together so that, for example, coded data can be identified as relating to a specific person. Example: a master list that contains the data code and the identifiers linked to the codes.*

***Obtain*** *means to possess or record in any fashion (writing, electronic document, video, email, voice recording, etc.) for research purposes and to retain for any length of time. This is different from accessing, which means to view or perceive data.*

**5.6.a.** Will you or any members of you team have access to any direct or indirect identifiers?

**Yes →** Describe which identifiers and for which data/specimens.

| We will collect names and phone numbers for scheduling of FGDs and KIIs with Aim 1 participants and to aid with retention in Aim 2. |
| --- |

**No →** Select the reason(s) why you (and all members of your team) will not have access to direct or indirect identifiers.

There will be no identifiers

Identifiers or the key have been (or will have been) destroyed before access.

There is an agreement with the holder of the identifiers (or key) that prohibits the release of the identifiers (or key) to study team members under any circumstances.

*This agreement should be available upon request from the IRB. Examples: a Data Use Agreement, Repository Gatekeeping form, or documented email.*

There are written policies and procedures for the repository/database/data management center that prohibit the release of the identifiers (or identifying link). This includes situations involving an Honest Broker.

There are other legal requirements prohibiting the release of the identifiers or key. Describe them below.

|  |
| --- |

**5.6.b**. Will you or any study team members obtain any direct or indirect identifiers?

**Yes →** Describe which identifiers and for which data/specimens.

| Study staff will collect contact information, phone numbers, and participant names as part of the enrollment process. |
| --- |

**No →** Select the reason(s) why you (and all members of your team) will not obtain direct or indirect identifiers.

There will be no identifiers.

Identifiers or the key have been (or will have been) destroyed before access.

There will be an agreement with the holder of the identifiers (or key) that prohibits the release of the identifiers (or key under any circumstances.

*This agreement should be available upon request from the IRB. Examples: a Data Use Agreement, Repository Gatekeeping form, or documented email.*

There are written policies and procedures for the repository/database/data management center that prohibit the release of the identifiers (or identifying link). This includes situations involving an Honest Broker.

There are other legal requirements prohibiting the release of the identifiers or key. Describe them below.

|  |
| --- |

**5.6.c.** If any identifiers will be obtained, indicate how the identifiers will be stored (and for which data). NOT: Do not describe the data security plan here, that information is requested in [question **9.6**.](#QNineDotSix)

Identifiers will be stored with the data. Describe the data to which this applies:

| Click or tap here to enter text. |
| --- |

Identifiers and study data will be stored separately but a link will be maintained between the identifiers and the study data (for example, through the use of a code). Describe the data to which this applies:

| We will store identifiers separately from study data but maintain a link with a code. This will include all identifiable data – names and phone numbers. |
| --- |

Identifiers and study data will be stored separately, with no link between the identifiers and the study data. Describe the data to which this applies:

| Click or tap here to enter text. |
| --- |

**5.6.d. Research collaboration**. Will individuals who provide coded information or specimens for the research also collaborate on other activities for this research? If yes, identify the activities and provide the name of the collaborator’s institution/organization.

*Examples include but are not limited to: (1) study, interpretation, or analysis of the data that results from the coded information or specimens; and (2) authorship on presentations or manuscripts related to this work*.

| No |
| --- |

**5.7. [DETERMINATION]**  **Protected Health Information (PHI).** Will participants’ identifiable PHI be accessed, obtained, used, or disclosed for any reason (for example, to identify or screen potential subjects, to obtain study data or specimens, for study follow-up) that does not involve the creation or obtaining of a Limited Data Set?

*PHI is individually identifiable healthcare record information or clinical specimens from an organization considered a “covered entity” by federal HIPAA regulations, in any form or media, whether electronic, paper, or oral.* ***You must answer yes to this question if the research involves identifiable health care records (e.g., medical, dental, pharmacy, nursing, billing, etc.), identifiable healthcare information from a clinical department repository, or observations or recordings of clinical interactions.***

*For information about what constitutes the UW Covered Entity, see UW Medicine Compliance* [*Patient Information Privacy Policy 101*](https://depts.washington.edu/comply/comp_101/) *and* [*diagram of the healthcare components*](http://depts.washington.edu/comply/docs/101_G1.pdf)*.*

**No →** Skip the rest of this question; go to [question **5.8**.](#QFiveDotEight)

**Yes →** Answer all of the questions below (**5.7.a.** through **5.7.f.**)

**5.7.a.** Describe the PHI and the reason for using it. *Be specific. For example, will any “free text” fields (such as physician notes) be accessed, obtained, or used?*

| Click or tap here to enter text. |
| --- |

**5.7.b**. Is any of the PHI located in Washington State?

**No**

**Yes**

**5.7.c**. Describe the pathway of how the PHI will be accessed or obtained, starting with the source/location and then describing the system/path/mechanism by which it will be identified, accessed, and copied for the research. *Be specific. For example: directly view records; search through a department’s clinical database; submit a request to Leaf.*

| Click or tap here to enter text. |
| --- |

**5.7.d.** For which PHI will subjects provide HIPAA authorization before the PHI is accessed, obtained and/or used?

| Click or tap here to enter text. |
| --- |

Confirm by checking the box that UW Medicine [HIPAA Authorization](https://www.washington.edu/research/forms-and-templates/template-hipaa-authorization/) form maintained on the HSD website will be used to access obtain, use, or disclose any UW Medicine PHI.

**Confirmed**

**5.7.e.** Will you obtain any HIPAA authorizations electronically (i.e., e-signature)?

**No**

**Yes →** Confirm by checking the box that you have read and understand the *Electronic Documentation of Consent* section of the [**WORKSHEET Consent Requirements and Waivers**](https://www.washington.edu/research/forms-and-templates/worksheet-consent/) the [**GUIDANCE Consent** *Documentation of Consent*](https://www.washington.edu/research/hsd/guidance/consent/#10) for information regarding the use of electronic signatures and HIPAA authorizations.

**Confirmed**

**5.7.f.** For which PHI will HIPAA authorization NOT be obtained from the subjects?

| Click or tap here to enter text. |
| --- |

Provide the following assurances by checking the boxes.

The minimum necessary amount of PHI to accomplish the purposes described in this application will be accessed, obtained and/or used.

The PHI will not be reused or disclosed to any other person or entity, except as required by law, for authorized oversight of the research study, or for other research for which the use or disclosure of PHI would be permitted.

The HIPAA “accounting for disclosures” requirement will be fulfilled, if applicable. See [UW Medicine Compliance Policy #104](http://depts.washington.edu/comply/comp_104/).

There will be reasonable safeguards to protect against identifying, directly or indirectly, any patient in any report of the research.

**5.8. [DETERMINATION]**  **Genomic data sharing**. Will the research obtain or generate genomic data?

**No**

**Yes** **→** Answer the question below.

**5.8.a.** Will genomic data from this research be sent to a national database (for example, NIH’s dbGaP database)?

**No**

**Yes** **→** Complete the [**SUPPLEMENT Genomic Data Sharing**](https://www.washington.edu/research/forms-and-templates/supplement-genomic-data-sharing/) and upload it to ***Zipline***.

**5.9. Whole genome sequencing**. For research involving biospecimens: Will the research include whole genome sequencing? NA

*Whole genome sequencing is sequencing of a human germline or somatic specimen with the intent to generate the genome or exome sequence of that specimen.*

**No**

**Yes**

**5.10. [DETERMINATION]**  **Cannabis (marijuana), hemp, and related compounds**. These questions are about: cannabis (any part of the plant in any form), hemp, cannabidiol (CBD), delta-8-THC, any product derived from cannabis or hemp, and related synthesized compounds. All UW research must comply with federal laws about cannabis because of conditions associated with the federal money that UW receives. Answer the questions below so that HSD can determine whether the federal laws apply to your specific situation. See the [UW Guidance on Research Involving Marijuana](https://www.washington.edu/research/policies/guidance-on-research-involving-marijuana/) for additional information.

**5.10.a.** Does your research involve any of the following? Check all that apply.

Study staff will obtain or handle any of the above items

Study will provide money to the participants to obtain any of the above items

Study participants will use or consume any of the above items on campus or in any UW-owned or leased facility

None of the above

**5.10.b.** If you checked any box except “None of the above”, provide the following information about each cannabis and related item your research will involve: Name of the item, how you will obtain it, the source, and whether it contains ≥0.3% THC (tetrahydrocannabinol).

| NA |
| --- |

**5.11**. **Possible secondary use or sharing of information, specimens, or subject contact information.** Please consider the broadest possible future plans and whether consent will be obtained now from the subjects for future sharing or research uses (which it may not be possible to describe in detail at this time).

*Many federal grants and contracts now require data or specimen sharing as a condition of funding, and many journals require data sharing as a condition of publication. “Sharing” may include (for example): informal arrangements to share banked data/specimens with other investigators; establishing a repository that will formally share with other researchers through written agreements; or sending data/specimens to a third-party repository/archive/entity such as the Social Science Open Access Repository (SSOAR), or the UCLA Ethnomusicology Archive.*

Answer all of the questions below. Write **NA** in response to questions **5.11.b** through **5.11.g** if sharing is unlikely or if the only sharing will be through the NIH Genomic Data Sharing described in [question **5.8**](#QFiveDotEight).

**5.11.a** Is this research funded by an NIH funding application submitted on or after January 25, 2023.

**No**

**Yes** **→** [NIH Data Management and Sharing Policy](https://sharing.nih.gov/data-management-and-sharing-policy/about-data-management-and-sharing-policy/research-covered-under-the-data-management-sharing-policy#after) applies to this research*.* Complete the rest of this section. If the policy applies and data will not be shared, provide the justification in response to **5.11.e** and write **NA** in response to the other questions.

**5.11.b.** Describe what will be stored for future use, including whether any direct or indirect (e.g., subject codes) identifiers will be stored.

| DATA MANAGEMENT AND SHARING PLAN  ELEMENT 1: DATA TYPE  A. Types and amount of scientific data expected to be generated in the project:  This proposal will generate data from approximately 1390 participants:  1) 630 participants (healthcare workers and perinatal clients) in formative data collection (Aim 1)  2) 560 participants (perinatal clients) in the longitudinal RCT cohort (Aim 2)  3) 200 participants (healthcare workers) providing care in the RCT (Aim 3)  Data include quantitative questionnaire data from all participant groups, transcripts of key informant interviews (KIIs) and focus group discussions (FGDs) from Aims 1 and 3 participants. Additionally, we will conduct costing activities that will generate time, resource and cost estimates for intervention delivery.  B. Scientific data that will be preserved and shared, and the rationale for doing so:  All study materials will be stored in a password-protected folder on a secure server maintained by the Kenyatta National Hospital (KNH) with back-up at the end of each day. All datasets will be subject to data standards such as documentation of methods of collection through codebooks, SOPs for data collection, and limitations associated with data collection. Data containing participant identifiers will not be shared, but will be preserved for six years after termination of the study, per institutional policy. Questionnaire data will be collected and stored electronically in HIPAA-compliant, secure, password-protected encrypted REDCap databases. These databases will be managed by the PIs and study coordinator.  A de-identified dataset will also be created to share publicly for dissemination and publication. The final versions of any de-identified data collected and/or generated will be made publicly available within 12 months of publication. Final data gathered from this project will be archived for data sharing purposes in Dryad, NIH’s recommended open-access generalist data repository. As an open access repository, Dryad only takes data with no personally identifiable information and deposits must be made open access to the public. No special software is needed to submit materials. In addition, the repository software creates persistent URLs that will not change over time.  C. Metadata, other relevant data, and associated documentation:  The study protocols, sample informed consent/assent forms, data collection instruments (including questionnaires or interview guides, as appropriate), and data dictionaries will be made accessible in data repositories where data are shared. Each variable in the data dictionaries will include a brief description of the item, question text, variable name/label, value name/label, and codes for missing values (including unknown, not reported, not applicable, or refusal). Documentation will be provided in PDF, Word, or Excel format. Deposit of materials to Dryad requires a minimum set of descriptive information (metadata) to be provided at the point of deposit. Some of this metadata will be automatically generated by the software used by Dryad; other pieces including author, title, and date, will be provided by the depositor.  ELEMENT 2: RELATED TOOLS, SOFTWARE AND/OR CODE  For the study use, questionnaire data will be collected in and accessed from the REDCap databases. Data entered into REDCap will be de-identified. These data will be analyzed using R statistical software and de-identified data and analysis files developed during the study will be stored in the KNH server and only accessible to investigators from KNH and UW. Focus group transcripts, time and motion data, and cost estimates will be stored as text documents and stored in the UW’s OneDrive. No specialized tools, software, and/or code are needed to access or manipulate shared scientific data. In addition to sharing study data, statistical analysis code will be made available as part of publications, by posting it in GitHub.  ELEMENT 3: STANDARDS  The FAIR Guiding Principles will be applied to the scientific data generated by this study: the data will be findable, accessible, interoperable, and reusable. The data will be assigned a persistent unique identifier and indexed in a searchable repository. An authentication and authorization procedure will be put in place to make the data accessible. Data will be shared in an interoperable format to allow for use by third parties. Finally, the data will be well-described through data dictionaries, codebooks, and reference lists to allow for replication in different settings. Dryad integrates with any SWORD-compliant repository.  ELEMENT 4: DATA PRESERVATION, ACCESS, AND ASSOCIATED TIMELINES  A. Repository where scientific data and metadata will be archived:  Scientific data arising from the supplement will be archived in Dryad within 12 months of publication date. Dryad is one of NIH’s recommended open-access generalist repositories and UW is a member of Dryad. Select study data and information will be made available to the research community free of charge.  B. How scientific data will be findable and identifiable:  Cleaned finalized data will be findable and identifiable using a persistent unique identifier (“digital object identifier”) assigned to the dataset by Dryad. Dryad also uses persistent unique identifiers for depositing authors (ORCID) and funders (Funder Registry).  C. When and how long the scientific data will be made available:  Cleaned de-identified data will be made available to other users within 12 months of an associated publication. Data deposited in Dryad is intended to remain permanently archived and available.  ELEMENT 5: ACCESS, DISTRIBUTION, OR REUSE CONSIDERATIONS  A. Factors affecting subsequent access, distribution, or reuse of scientific data:  De-identified and cleaned data will be shared as allowed by the participant’s informed consent, PI approval, and the institutional certification, following associated publication.  B. Whether access to scientific data will be controlled:  De-identified data deposited in Dryad will be openly available to the public under the terms of a Creative Commons Zero (CC0) waiver.  C. Protections for privacy, rights, and confidentiality of human research participants:  Throughout and following data collection procedures, all participant identifiers will be stored in a HIPAA-compliant, secure, password-protected encrypted REDCap database separate from the main study databases. Identifying information can only be accessed by study staff approved by the PIs. Informed consent documents completed at enrollment into the cohorts or during other data collection activities such as prior to focus group discussions and time and motion activities include explicit language about storage of data for use in future research and sharing of de-identified data. Participants will not be contacted or re-consented for future sharing or accessing data through repositories. Only de-identified data will be archived and available for sharing. |
| --- |

**5.11.c.** Describe what will be shared with other researchers or with a repository/database/registry, including whether direct identifiers will be shared and (for specimens) what data will be released with the specimens. If shared through a repository, specify if it is unrestricted access (i.e., publicly accessible).

| As described in b |
| --- |

**5.11.d.** Who will oversee and/or manage the sharing?

| ELEMENT 6: OVERSIGHT OF DATA MANAGEMENT AND SHARING  Monitoring of and compliance with this Data Management and Sharing Plan will be the responsibility of the PIs and study team. The PIs will provide oversight and ensure appropriate, ethical, data management adherence to KNH and UW standards. Issues related to confidentiality, security, and intellectual property will adhere to guidelines set forth by KNH and UW. |
| --- |

**5.11.e.** Describe the possible future uses, and any limitations or restrictions on future uses or users.

*Examples of limitations:*

- *Consent prohibits or limits the scope of sharing and use (e.g., consent states that data will be used only for cardiovascular research)*
- *Privacy or safety or research participants would be compromised (e.g., there is risk of reidentification and/or harm)*
- *Explicit federal, state, or local, or Tribal law, regulation, or policy prohibits disclosure*
- *Restrictions imposed by existing or anticipated agreements (e.g., with third party funders, partners, with repositories, medical centers providing health information under a data use agreement)*

| As described in b |
| --- |

**5.11.f**. Consent. Will consent be obtained now from subjects for the secondary use, banking, and/or future sharing?

**No**

**Yes →** Be sure to include the information about this consent process in the consent form (if there is one) and in the answers to the consent question in Section 8.

**5.11.g**. Withdrawal. Will subjects be able to withdraw their data/specimens from secondary use, banking or sharing?

**No**

**Yes →** Describe how, and whether there are any limitations on withdrawal.

*Example: data can be withdrawn from the repository but cannot be retrieved after they are released.*

| Click or tap here to enter text. |
| --- |

**5.11.h.** Agreements for sharing or release. Confirm by checking the box that the sharing or release will comply with UW (and, if applicable, UW Medicine) policies that require a formal agreement with the recipient for release of data or specimens to individuals or entities other than federal databases.

*Data Use Agreements or Gatekeeping forms are used for data; Material Transfer Agreements are used for specimens (or specimens plus data). Do not attach any template agreement forms; the IRB neither reviews nor approves them.*

**Confirmed**

**5.12. Communication with subjects during the study**. Describe the types of communication (if any) the research team will have with already-enrolled subjects during the study. Provide a description instead of the actual materials themselves.

*Examples: email, texts, phone, or letter reminders about appointments or about returning study materials such as a questionnaire; requests to confirm contact information.*

| Phone and texts for reminders on study appointments and questionnaires |
| --- |

**5.13**. **Future contact with subjects**. Is there a plan to retain any contact information for subjects so that they can be contacted in the future?

**No**

**Yes →** Describe the purpose of the future contact, and whether use of the contact information will be limited to the study team; if not, describe who else could be provided with the contact information. Describe the criteria for approving requests for information.

*Examples: inform subjects about other studies; ask subjects for additional information or medical record access that is not currently part of the study proposed in this application; obtain another sample.*

| Participants will be asked if they are willing to be contacted for future similar studies. Those willing to be contacted will have their contact information only accessible to the study team. |
| --- |

**5.14. Alternatives to participation**. Are there any alternative procedures or treatments that might be advantageous to the subjects?

*If there are no alternative procedures or treatments, select “No”. Examples of advantageous alternatives: earning extra class credit in some time-equivalent way other than research participation; obtaining supportive care or a standard clinical treatment from a health care provider instead of participating in research with an experimental drug.*

**No**

**Yes →** Describe the alternatives.

| Click or tap here to enter text. |
| --- |

**5.15. Upload to *Zipline*** all data collection forms (if any) that will be directly used by or with the subjects, and any scripts/talking points that will be used to collect the data. Do not include data collection forms that will be used to abstract data from other sources (such as medical or academic records), or video recordings.

- ***Examples****: survey, questionnaires, subject logs or diaries, focus group questions.*
- ***NOTE****: Sometimes the IRB can approve the general content of surveys and other data collection instruments rather than the specific form itself. This prevents the need to submit a modification request for future minor changes that do not add new topics or increase the sensitivity of the questions. To request this general approval, use the text box below to identify the questionnaires/surveys/ etc. for which you are seeking this more general approval. Then briefly describe the scope of the topics that will be covered and the most personal and sensitive questions. The HSD staff person who screens this application will let you know whether this is sufficient or whether you will need to provide more information.*
- ***For materials that cannot be uploaded****: upload screenshots or written descriptions that are sufficient to enable the IRB to understand the types of data that will be collected and the nature of the experience for the participant. You may also provide URLs (website addresses) or written descriptions below. Examples of materials that usually cannot be uploaded: mobile apps; computer-administered test; licensed and restricted standardized tests.*
- ***For data that will be gathered in an evolving way****: This refers to data collection/questions that are not pre-determined but rather are shaped during interactions with participants in response to observations and responses made during those interactions. If this applies to the proposed research, provide a description of the process by which the data collection/questions will be established during the interactions with subjects, how the data collection/questions will be documented, the topics likely to be addressed, the most sensitive type of information likely to be gathered, and the limitations (if any) on topics that will be raised or pursued.*

Use this text box (if desired) to provide:

- Short written descriptions of materials that cannot be uploaded, such as URLs
- A description of the process that will be used for data that will be gathered in an evolving way.
- The general content of questionnaires, surveys and similar instruments for which general approval is being sought. (See the **NOTE** bullet point in the instructions above.)

| We have uploaded a description of data variables to Zipline. We request general approval for the topics in our questionnaires and discussion guides based on the topics described in the uploaded attachment. |
| --- |

6. CHILDREN (MINORS) AND PARENTAL PERMISSION

**6.1. [DETERMINATION]**  **Involvement of minors**. Does the research include minors (children)?

**Minor or child** means someone who has not yet attained the legal age for consent for the research procedures, as described in the applicable laws of the jurisdiction in which the research will be conducted. This may or may not be the same as the definition used by funding agencies such as the National Institutes of Health.

- In Washington State the generic age of consent is 18, meaning that anyone under the age of 18 is considered a child.
- There are some procedures for which the age of consent is much lower in Washington State.
- The generic age of consent may be different in other states, and in other countries.

**No →** Go to [Section 8](#CONSENTOFADULTS).

**Yes →** Provide the age range of the minor subjects for this study and the legal age for consent in the study population(s). If there is more than one answer, explain.

| Click or tap here to enter text. |
| --- |

**Don’t know →** This means is it not possible to know the age of the subjects. For example, this may be true for some research involving social media, the Internet, or a dataset that is obtained from another researcher or from a government agency. Go to [Section 8](#CONSENTOFADULTS).

**6.2. Parental permission.** Parental permission means actively obtaining the permission of the parents. This is not the same as “passive” or “opt out” permission where it is assumed that parents are allowing their children to participate because they have been provided with information about the research and have not objected or returned a form indicating they don’t want their children to participate.

**6.2.a.** Will parental permission be obtained for:

All of the research procedures **→** Go to [question **6.2.b**](#QSixDotTwoDotB)**.**

None of the research procedures **→** Use the table below to provide justification and skip question **6.2.b.**

Some of the research procedures **→** Use the table below to identify the procedures for which parental permission will not be obtained.

*Be sure to consider all research procedures and plans, including screening, future contact, and sharing/banking of data and specimens for future work.*

| **Children Group^1^** | **Describe the procedures or data/specimen collection (if any) for which there will be NO parental permission^2^** | **Reason why parental permission will not be obtained** | **Will parents be informed about the research?^3^** | |
| --- | --- | --- | --- | --- |
|  |  |  | **YES** | **NO** |
| Click or tap here to enter text. | Click or tap here to enter text. | Click or tap here to enter text. |  |  |
| Click or tap here to enter text. | Click or tap here to enter text. | Click or tap here to enter text. |  |  |
| Click or tap here to enter text. | Click or tap here to enter text. | Click or tap here to enter text. |  |  |
| Click or tap here to enter text. | Click or tap here to enter text. | Click or tap here to enter text. |  |  |
| Click or tap here to enter text. | Click or tap here to enter text. | Click or tap here to enter text. |  |  |

*Table footnotes*

1. *If the answer is the same for all children groups or all procedures: collapse the answer across the groups and/or procedures.*
2. *If identifiable information or biospecimens will be obtained without parent permission, any waiver granted by the IRB does not override parents’ refusal to provide broad consent (for example, through the Northwest Biotrust).*
3. *Will parents be informed about the research beforehand even though active permission is not being obtained?*

**6.2.b.** Indicate the plan for obtaining parental permission. One or both boxes must be checked.

Both parents, unless one parent is deceased, unknown, incompetent, or not reasonably available, or when only one parent has legal responsibility for the care and custody of the child.

One parent, even if the other parent is alive, known, competent, reasonably available, and shares legal responsibility for the care and custody of the child.

*This is all that is required for minimal risk research.*

If both are checked explain:

| Click or tap here to enter text. |
| --- |

**6.3. Children who are wards**. Will any of the children be wards of the State or any other agency, institution, or entity?

**No**

**Yes** **→** An advocate may need to be appointed for each child who is a ward. The advocate must be in addition to any other individual acting on behalf of the child as guardian or in loco parentis. The same individual can serve as advocate for all children who are wards.

Describe who will be the advocate(s). The description must address the following points:

- Background and experience
- Willingness to act in the best interests of the child for the duration of the research
- Independence of the research, research team, and any guardian organization

| Click or tap here to enter text. |
| --- |

**6.4. UW Office of the Youth Protection Coordinator**. If the project involves interaction (in-person or remotely) with individuals under the age of 18, researchers must comply with UW Administrative Policy Statement 10.13 and the requirements listed at [this website](https://www.washington.edu/youth/policy/protecting-youth-at-uw-aps-10-13/). This includes activities that are deemed to be Not Research or Exempt. It does not apply to third-party led research (i.e., research conducted by a non-UW PI). [Information and FAQs](https://www.washington.edu/youth/policy/protecting-youth-at-uw-aps-10-13/youth-program-faqs/#researchers) for researchers are available.

**This point is advisory only; there is no need to provide a response.**

7. ASSENT OF CHILDREN (MINORS)

*Go to* [*Section 8*](#CONSENTOFADULTS) *if your research does not involve children (minors).*

When designing assent processes and forms, researchers should first review the [**GUIDANCE Consent** *Protected and Vulnerable Populations*](https://www.washington.edu/research/hsd/guidance/consent/#6) and [**TIPSHEET Consent**](https://www.washington.edu/research/hsd/guidance/consent/consent-tip-sheets/) *Assent and Legally Authorized Representative.*

**7.1. Assent of children (minors).** Though children do not have the legal capacity to “consent” to participate in research, they should be involved in the process if they are able to “assent” by having a study explained to them and/or by reading a simple form about the study, and then verbally expressing whether they want to participate. They may also provide a written assent if they are older. See [**GUIDANCE Consent** *Protected and Vulnerable Populations*](https://www.washington.edu/research/hsd/guidance/consent/#6) and [**WORKSHEET Children**](https://www.washington.edu/research/forms-and-templates/worksheet-children/) for circumstances in which a child’s assent may be unnecessary or inappropriate.

**7.1.a.** Will assent be obtained for:

All research procedures and child groups **→** Go to [question **7.2**](#QSevenDotTwo).

None of the research procedures and child groups **→** Use the table below to provide justification, then skip to [question **7.6**](#QSevenDotSix)**.**

Some of your research procedures and child groups **→** Use the table below to identify the procedures for which assent will not be obtained.

*Be sure to consider all research procedures and plans, including screening, future contact, and sharing/banking of data and specimens for future work.*

| **Children Group^1^** | **Describe the procedures or data/specimen collection (if any) for which assent will not be obtained** | **Reason why assent will not be obtained** |
| --- | --- | --- |
| Click or tap here to enter text. | Click or tap here to enter text. | Click or tap here to enter text. |
| Click or tap here to enter text. | Click or tap here to enter text. | Click or tap here to enter text. |
| Click or tap here to enter text. | Click or tap here to enter text. | Click or tap here to enter text. |
| Click or tap here to enter text. | Click or tap here to enter text. | Click or tap here to enter text. |
| Click or tap here to enter text. | Click or tap here to enter text. | Click or tap here to enter text. |

*Table footnotes*

1. *If the answer is the same for all children groups or all procedures, collapse your answer across the groups and/or procedures.*

**7.2. Assent process**. Describe how assent will be obtained, for each child group. If the research involves children of different ages, answer separately for each group. If the children are non-English speakers, include a description of how their comprehension of the information will be evaluated.

| Click or tap here to enter text. |
| --- |

**7.3. Dissent or resistance.** Describe how a child’s objection or resistance to participation (including non-verbal indications) will be identified during the research, and what the response will be.

| Click or tap here to enter text. |
| --- |

**7.4. E-consent.** Will any electronic processes (email, websites, electronic signatures, etc.) be used to present assent information to subjects/and or to obtain documentation (signatures) of assent? If yes, describe how this will be done.

| Click or tap here to enter text. |
| --- |

**7.5. Documentation of assent.** Which of the following statements describes whether documentation of assent will be obtained?

None of the research procedures and child groups **→** Use the table below to provide justification, then go to [question **7.5.b.**](#QSevenDotFiveDotB)

All of the research procedures and child groups **→** Go to [question **7.5.a**](#QSevenDotFiveDotA)., do not complete the table.

Some of the research procedures and/or child groups **→** Complete the table below and then go to [question **7.5.a.**](#QSevenDotFiveDotA)

| **Children Group^1^** | **Describe the procedures or data/specimen collection (if any) for which assent will NOT be documented** |
| --- | --- |
| Click or tap here to enter text. | Click or tap here to enter text. |
| Click or tap here to enter text. | Click or tap here to enter text. |
| Click or tap here to enter text. | Click or tap here to enter text. |
| Click or tap here to enter text. | Click or tap here to enter text. |
| Click or tap here to enter text. | Click or tap here to enter text. |

*Table footnotes*

1. *If the answer is the same for all children groups or all procedures, collapse your answer across the groups and/or procedures.*

**7.5.a.** Describe how assent will be documented. If the children are functionally illiterate or are not fluent in English, include a description of the documentation process for them.

| Click or tap here to enter text. |
| --- |

**7.5.b.** Upload all assent materials (talking points, videos, forms, etc.) to ***Zipline***. Assent materials are not required to provide all of the standard elements of adult consent; the information should be appropriate to the age, population, and research procedures. The documents should be in Word, if possible.

**7.6. Children who reach the legal age of consent during participation in longitudinal research.**

When children are enrolled at a young age and continue for many years, it is best practice to re-obtain assent (or to obtain it for the first time, if it was not obtained at the beginning of their participation).

When children reach the legal age of consent, informed consent must be obtained from the now-adult subject for (1) any ongoing interactions or interventions with the subjects, or (2) the continued analysis of specimens or data for which the subject’s identify is readily identifiable to the researcher, unless the IRB waives this requirement.

**7.6.a**. Describe the plans (if any) to re-obtain assent from children.

| Click or tap here to enter text. |
| --- |

**7.6.b.** Describe the plans (if any) to obtain consent for children who reach the legal age of consent.

- If adult consent will be obtained from them, describe what will happen regarding now-adult subjects who cannot be contacted.
- If consent will not be obtained or will not be possible, explain why.

| Click or tap here to enter text. |
| --- |

**7.7. Other regulatory requirements.** (This is for information only; no answer or response is required.) Researchers are responsible for determining whether their research conducted in schools, with student records, or over the Internet comply with permission, consent, and inspection requirements of the following federal regulations:

- PPRA – Protection of Pupil Rights Amendment
- FERPA – Family Education Rights and Privacy Act
- COPPA – Children’s Online Privacy Protection Act

8 CONSENT OF ADULTS

| When designing consent process and forms, researchers should first review the [**GUIDANCE Consent**](https://www.washington.edu/research/hsd/guidance/consent/) and [**TIPSHEET Consent**](https://www.washington.edu/research/hsd/guidance/consent/consent-tip-sheets/). The topics of *A Foundation for Meaningful Consent* and *The Key Information Requirement* are particularly important for ensuring subject comprehension and voluntary participation in research. Information about parental permission can be found in the [**GUIDANCE Consent** *Protected and Vulnerable Populations.*](https://www.washington.edu/research/hsd/guidance/consent/#6) |
| --- |

**Review the following definitions before answering the questions in this section.**

| **TERM** | **DEFINITION** |
| --- | --- |
| **CONSENT** | is the process of informing potential subjects about the research and asking them whether they want to participate. It does not necessarily include the signing of a consent form. |
| **CONSENT DOCUMENTATION** | refers to how a subject’s decision to participate in the research is documented. This is typically obtained by having the subject sign a consent form. |
| **CONSENT FORM** | is a document signed by subjects, by which they agree to participate in the research as described in the consent form and in the consent process. |
| **ELEMENTS OF CONSENT** | are specific information that is required to be provided to subjects. |
| **CHARACTERISTICS OF CONSENT** | are the qualities of the consent process as a whole. These are:   - Consent must be legally effective. - The process minimizes the possibility of coercion or undue influence. - Subjects or their representatives must be given sufficient opportunity to discuss and consider participation. - The information provided must:   - Begin with presentation of key information (for consent materials over 2,000 words).   - Be what a reasonable person would want to have.   - Be organized and presented so as to facilitate understanding.   - Be provided in sufficient detail.   - Not ask or appear to ask subjects to waive their rights. |
| **PARENTAL PERMISSION** | is the parent’s active permission for the child to participate in the research. Parental permission is subject to the same requirements as consent, including written documentation of permission and required elements. |
| **SHORT FORM CONSENT** | is an alternative way of obtaining written documentation of consent that is most commonly used for the unanticipated enrollment of individuals who are illiterate or whose language is one for which translated consent forms are not available. |
| **WAIVER OF CONSENT** | means there is IRB approval for not obtaining consent or for not including some of the elements of consent in the consent process.  **NOTE**: if you plan to obtain identifiable information or identifiable biospecimens without consent, any waiver granted by the IRB does not override a subject’s refusal to provide broad consent (for example, the Northwest Biotrust). |
| **WAIVER OF DOCUMENTATION OF CONSENT** | means that there is IRB approval for not obtaining written documentation of consent. |

**8.1. Groups**. Identify the groups to which the answers in this section apply:

**Adult** subjects

**Parents** who are providing permission for their children to participate in research

**→** *If you selected* ***PARENTS****, the word “consent” below should also be interpreted as applying to parental permission and “subjects” should also be interpreted as applying to the parents.*

**8.2. The consent process and characteristics.** This series of questions is about whether consent will be obtained for all procedures except recruiting and screening, and, if yes, how.

*The issue of consent for recruiting and screening activities is address in* [*question 4.7*](#QFourDotSeven)*. You do not need to repeat your answer to question 4.7.*

**8.2.a.** Are there any procedures for which consent will not be obtained?

**No**

**Yes →** Use the table below to identify the procedures for which consent will not be obtained. “All” is an acceptable answer for some studies.

*Be sure to consider all research procedures and plans, including future contact, and sharing/banking of data and specimens for future work.*

| **Group**[**^1^**](#tableFootnote1) | **Describe the procedures of data/specimen collection (if any) for which there will be NO consent process** | **Reason why consent will not be obtained** | **Will subjects be provided with info about the research after they finish? (Check Yes or No)** | |
| --- | --- | --- | --- | --- |
|  |  |  | **YES** | **NO** |
| Click or tap here to enter text. | Click or tap here to enter text. | Click or tap here to enter text. |  |  |
| Click or tap here to enter text. | Click or tap here to enter text. | Click or tap here to enter text. |  |  |
| Click or tap here to enter text. | Click or tap here to enter text. | Click or tap here to enter text. |  |  |
| Click or tap here to enter text. | Click or tap here to enter text. | Click or tap here to enter text. |  |  |
| Click or tap here to enter text. | Click or tap here to enter text. | Click or tap here to enter text. |  |  |
| Click or tap here to enter text. | Click or tap here to enter text. | Click or tap here to enter text. |  |  |

*Table footnotes*

*1. If the answer is the same for all groups, collapse your answer across the groups and/or procedures.*

**8.2.b.** Describe the consent process, if consent will be obtained for any or all procedures, for any or all groups. Address groups and procedures separately if the consent processes are different.

*Be sure to include:*

- *The location/setting where consent will be obtained*
- *Who will obtain consent (refer to positions, roles, or titles, not names)*
- *How subjects will be provided sufficient opportunity to discuss the study with the research team and consider participation.*

| Study staff will obtain informed consent from study participants who are eligible for the study in a private location in the facility. Participants will be informed of the study procedures and will be encouraged to ask questions and seek clarification on any of the research procedures.  Consent for eligibility screening will be obtained verbally.  Consent for enrollment in study activities will be obtained in writing. A copy of the consent will be provided for their future reference. |
| --- |

**8.2.c.** Comprehension. Describe the methods that will be used to ensure or test the subjects’ understanding of the information during the consent process.

| Consent and study details will be presented in a language that the subject understands. After providing study information and answering participant questions, the participants will be asked simple questions to ascertain their understanding of the study. Questions include:   1. What is the purpose of this study? 2. How long are you being asked to participate in this study? 3. Will we access your medical records as part of this study? 4. When can you decide not to participate in this study? |
| --- |

**8.2.d.** Influence. Does the research involve any subject groups that might find it difficult to say “no” to participation because of the setting or their relationship with someone on the study team, even if they aren’t pressured to participate?

*Examples: Student participants being recruited into their teacher’s research; patients being recruited into their healthcare provider’s research; study team members who are participants; outpatients recruited from an outpatient surgery waiting room just prior to their surgery.*

**No**

**Yes** **→** Describe what will be done to reduce any effect of the setting or relationship on the participation decision.

*Examples: a study coordinator will obtain consent instead of the subject’s physician; the researcher will not know which subjects agreed to participate; subjects will have two days to decide after hearing about the study.*

| Click or tap here to enter text. |
| --- |

**8.2.e.** Information provided is tailored to the needs of the subject population. Describe the basis for concluding that the information that will be provided to subjects (via written or oral methods) is what a *reasonable member* of the *subject population(s)* would want to know. If the research consent materials contain a key information section, also describe the basis for concluding that the information present in that section is that which is *most likely* to assist the selected subject population with making a decision. See [**GUIDANCE Consent** *Key Information*](https://www.washington.edu/research/hsd/guidance/consent/#5) and [**EXAMPLE Key Information**](https://www.washington.edu/research/forms-and-templates/example-key-info/).

*For example: Consultation with publications about research subjects’ preferences, disease-focused nonprofit groups, patient interest groups, or other researchers/study staff with experience with the specific population. It may also involve directly consulting selected members of the study population.*

| Information provided in the consent form describes all the activities that the participants will be involved in put in a simple clear language (including local translations that will be done) and is what has been agreed on by the team of experienced researchers and implementors in the study. |
| --- |

**8.2.f.** Ongoing process, new information, and reconsent.

For research that involves multiple or continued interaction with subjects over time, describe the opportunities (if any) that will be given to subjects to ask questions or to change their minds about participating.

Throughout the course of the study, subjects may need to be notified about new information. This might take the form of a verbal or written communication or may require subjects to provide reconsent. When a modification is submitted in which subjects need to be informed about new information, describe the method and process the research team will use to provide this information.

*See* [***TIPSHEET Consent***](https://www.washington.edu/research/hsd/guidance/consent/consent-tip-sheets/) *Reconsent and Ongoing Subject Communication and* [***GUIDANCE Consent Reconsent and Ongoing Subject Communication***](https://www.washington.edu/research/hsd/guidance/consent/#11) *for details.*

| Participants will be made aware of any changes in the protocol that will require them to re-consent. |
| --- |

**8.3**. **Electronic presentation of consent information**. Will any part of the consent-related information be provided electronically for some, or all of the subjects?

*This refers to the use of electronic systems and processes instead of (or in addition to) a paper consent form. For example, an emailed consent form, a passive or an interactive website, graphics, audio, video podcasts. See* ***GUIDANCE Consent*** [*Electronic Consent*](https://www.washington.edu/research/hsd/guidance/consent/#9) *and* [*Documentation of Consent*](https://www.washington.edu/research/hsd/guidance/consent/#10) *for information about electronic consent requirements at UW.*

**No** **→** Skip to [question **8.4.**](#QEightDotFour)

**Yes** **→** Answer questions **8.3.a.** through **8.3.e.**

**8.3.a.** Describe the electronic consent methodology and the information that will be provided.

*All information materials must be made available to the IRB. Website content should be provided as a Word document. It is considered best practice to give subjects information about multi-page/multi-screen information that will help them assess how long it will take them to complete the process. For example, telling them that it will take about 15 minutes, or that it involves reading six screens or pages.*

| All information on the informed consent will be available on REDCap and participants will sign electronically if they agree to the consent information discussed. Study research assistants will be with the participants during the whole consenting process to answer questions and navigate the pages. Signing will be done on the study tablets. |
| --- |

**8.3.b.** Describe how the information can be navigated (if relevant).

*For example, will the subject be able to proceed forward or backward within the system, or to stop and continue at a later time?*

| The participant will be able to navigate backward and forward through the REDCap consenting module. The process will be completed in one sitting with study staff. |
| --- |

**8.3.c.** In a standard paper-based consent process, the subjects generally have the opportunity to go through the consent form with study staff and/or to ask study staff about any question they may have after reading the consent form. Describe what will be done, if anything, to facilitate the subject’s comprehension and opportunity to ask questions when consent information is presented electronically. Include a description of any provisions to help ensure privacy and confidentiality during this process.

*Examples: hyperlinks, help text, telephone calls, text messages or other type of electronic messaging, video conference, live chat with remotely located study team members.*

| All consent procedures will take place in the presence of a study staff member who will be able to answer any questions from potential participants and provide needed assistance to facilitate consenting for all interested/eligible potential participants. Signing will be done on the study tablets |
| --- |

**8.3.d.** What will happen if there are individuals who wish to participate but who do not have access to the consent methodology being used, or who do not wish to use it? Are there alternative ways in which they can obtain the information, or will there be some assistance available? If this is a clinical trial, these individuals cannot be excluded from the research unless there is a compelling rationale.

*For example, consider individuals who lack familiarity with electronic systems, have poor eyesight or impaired motor skills, or who do not have easy email or internet access.*

| All consent procedures will take place in person in the presence of a study staff member, using study tablets. Staff will provide needed assistance to facilitate consenting for all interested/eligible potential participants. |
| --- |

**8.3.e.** How will the research team ensure continued accessibility of consent materials and information during the study?

| A copy of the consent will be given to the participants |
| --- |

**8.3.f**. How will additional information be provided to subjects during the research, including any significant new findings (such as new risk information). If this is not an issue, explain why.

| Any additional information will be provided during the study visits |
| --- |

**8.4. Written documentation of consent**. Which of the statements below describe whether documentation of consent will be obtained? NOTE: This question does not apply to screening and recruiting procedures which have already been addressed in [question **4.7**](#QFourDotSeven)**.**

*Documentation of consent that is obtained electronically is not considered written consent unless it is obtained by a method that allows verification of the individual’s signature. In other words, saying “yes” by email is rarely considered to be written documentation of consent.*

**8.4.a.** Is written documentation being obtained for:

**None** of the research procedures **→** Use the following table to provide justification then go to [question **8.5.**](#QEightDotFive)

**All** of the research procedures **→** Do not complete the following table, go to [question **8.4.b.**](#QEightDotFourDotB)

**Some** of the research procedures **→** Use the following table to identify the procedures for which written documentation of consent will not be obtained from adult subjects.

| **Adult subject group**[**^1^**](#tableFootnote2) | **Describe the procedures or data/specimen collection (if any) for which there will be NO documentation of consent** | **Will they be provided with a written statement describing the research (optional)?**  **(Check Yes or No)** | |
| --- | --- | --- | --- |
|  |  | **YES** | **NO** |
| Aim 1, 2, 3 participants | Eligibility screening |  |  |
| Click or tap here to enter text. | Click or tap here to enter text. |  |  |
| Click or tap here to enter text. | Click or tap here to enter text. |  |  |
| Click or tap here to enter text. | Click or tap here to enter text. |  |  |
| Click or tap here to enter text. | Click or tap here to enter text. |  |  |

*Table footnotes*

*1. If the answer is the same for all adult groups or all procedures, collapse the answer across the groups and/or procedures.*

**8.4.b. Electronic consent signature**. For studies in which documentation of consent will be obtained, will subjects use an electronic method to provide their consent signature?

- *See* [***GUIDANCE Consent*** *Documentation of Consent*](https://www.washington.edu/research/hsd/guidance/consent/#10) *and* [***INSTRUCTIONS UW E-Signature Tools***](https://www.washington.edu/research/hsd/guidance/consent/econsent) *for information about options (including* ***REDCap*** *e-signature and the* ***DocuSign*** *system) and any associated requirements.*
- *FDA-regulated studies must use a system that complies with the FDA’s “Part 11” requirements about electronic systems and records. Note that the UW-IT supported DocuSign e-signature system does not meet this requirement.*
- *Having subjects check a box at the beginning of an emailed or web-based questionnaire is not considered legally effective documentation of consent.*

**No**

**Yes** **→** Indicate which methodology will be used

**UW ITHS REDCap** (excludes REDCap Mobile application, which is a separate software application for use with a mobile device for consent when internet service is absent or unreliable)

**Other REDCap installation** **→** Please name the institutional version you will be using (e.g., Vanderbilt, Univ. of Cincinnati) in the following field and provide a completed [**SUPPLEMENT Other REDCap Installation**](https://www.washington.edu/research/forms-and-templates/supplement-other-redcap-installation/) with your submission.

| Click or tap here to enter text. |
| --- |

**UW DocuSign**

**Other** **→** Please describe in the following field and provide a signed [**TEMPLATE Other E-signature Attestation Letter**](https://www.washington.edu/research/forms-and-templates/template-other-esignature-attestation-letter/) with your submission.

| Click or tap here to enter text. |
| --- |

**8.4.b.1.** Is this method legally valid in the jurisdiction where the research will occur?

*NOTE: UW ITHS REDCap (excludes REDCap Mobile application) and UW DocuSign have been vetted for compliance with Washington State and federal laws regarding electronic signatures.*

**No**

**Yes** **→** What is the source of information about legal validity?

| Click or tap here to enter text. |
| --- |

**8.4.b.2.** Will verification of the subject’s identity be obtained if the signature is not personally witnessed by a member of the study team? Note that this is required for FDA-regulated studies.

*See the* [***GUIDANCE Consent*** *Documentation of Consent*](https://www.washington.edu/research/hsd/guidance/consent/#10) *for information and examples*

**No** **→** Provide the rationale for why this is not required or necessary to protect subjects or the integrity of the research. Also, what would be the risks to the actual subject if somebody other than the intended signatory provides the consent signature?

| Click or tap here to enter text. |
| --- |

**Yes** **→** Describe how subject identity will be verified, providing a non-technical description that the reviewer will understand.

| Click or tap here to enter text. |
| --- |

**8.4.b.3.** How will the requirement be met to provide a copy of the consent information (consent form) to individuals who provide an e-signature?

*The copy can be paper or electronic and may be provided on an electronic storage device or via email. If the electronic consent information uses hyperlinks or other websites or podcasts to convey information specifically related to the research, the information in these hyperlinks should be included in the copy provided to the subjects and the website must be maintained for the duration of the entire study.*

| Click or tap here to enter text. |
| --- |

**8.4.c. Barriers to written documentation of consent**. There are many possible barriers to obtaining written documentation of consent. Consider, for example, individuals who are functionally illiterate; do not read English well; or have sensory or motor impairments that may impede the ability to read and sign a consent form.

**8.4.c.1.** Describe the plans (if any) for obtaining written documentation of consent from potential subjects who may have difficulty with the standard documentation process (that is, reading and signing a consent form).

*Examples of solutions: Translated consent forms; use of the Short Form consent process; reading the form to the person before they sign it; excluding individuals who cannot read and understand the consent form.*

| A witness may sign on behalf of the participant. A witness will be an individual who must be independent of the study, literate and selected by the participant |
| --- |

**8.5. Non-English-speaking or-reading adult subjects**. Will the research enroll adult subjects who do not speak English or who lack fluency or literacy in English?

**No**

**Yes** **→** Describe the process that will be used to ensure that the oral and written information provided to them during the consent process and throughout the study will be in a language readily understandable to them and (for written materials such as consent forms or questionnaires) at an appropriate reading/comprehension level.

| Translations will be done in Kiswahili and Dholuo |
| --- |

**8.5.a.** Interpretation. Describe how interpretation will be provided, and when. Also, describe the qualifications of the interpreter(s) - for example, background, experience, language proficiency in English and in the other language, certification, other credentials, familiarity with the research related vocabulary in English and the target language.

| In the case where participant requires interpretation even with translated documents, a witness may be called in to interpret. This is an individual independent of the study and chosen by participant. |
| --- |

**8.5.b.** Translations. Describe how translations will be obtained for all study materials (not just consent forms). Also, describe the method for ensuring that the translations meet the UW IRB’s requirement that translated documents will be linguistically accurate, at an appropriate reading level for the participant population, and culturally sensitive for the local in which they will be used.

Check this box to confirm that before using them with subjects, you will upload in *Zipline* all translated consent materials that will be provided to subjects in written or electronic form (per [**HSD policy**](https://www.washington.edu/research/hsd/guidance/consent/#8a2)).

*If the IRB determines that your study is greater than minimal risk, or otherwise determines it is required, you will need to work with your translator to provide a* [***TEMPLATE Translation Attestation***](https://www.washington.edu/research/forms-and-templates/template-translation-attestation/)*. If the attestation is required, you will be informed by the IRB during the course of the review.*

| Translations done will meet the UW IRB’s requirement. Translated documents will be linguistically accurate, at an appropriate reading level for the participant population, and culturally sensitive for the local in which they will be used. Professional translating services will be sought. All translations will then be back translated by different individuals from those who translated to ensure that the context and meanings are maintained. Once the back and forth of translated documents are agreeable then that is when they will be used. |
| --- |

**8.6. [DETERMINATION]**  **Deception**. Will information be deliberately withheld, or will false information be provided, to any of the subjects?

*NOTE: “Blinding” subjects to their study group/condition/arm is not considered to be deception, but not telling them ahead of time that they will be subjects to an intervention or about the purpose of the procedure(s) is deception.*

**No**

**Yes** **→** Describe what information and why.

*Example: it may be necessary to deceive subjects about the purpose of the study (describe why).*

| Click or tap here to enter text. |
| --- |

**8.6.a.** Will subjects be informed beforehand that they will be unaware of or misled regarding the nature or purposes of the research? (Note: this is not necessarily required.)

**No**

**Yes**

**8.6.b.** Will subjects be debriefed later? (Note: this is not necessarily required.)

**No →** Provide your reasoning for not debriefing subjects.

| Click or tap here to enter text. |
| --- |

**Yes** **→** Describe how and when this will occur. Upload any debriefing materials, including talking points or a script, to ***Zipline***.

| Click or tap here to enter text. |
| --- |

**8.7. [DETERMINATION]** **Cognitively impaired adults, and other adults unable to consent**. Will such individuals be included in the research?

*Examples: individuals with Traumatic Brain Injury (TBI) or dementia; individuals who are unconscious, or who are significantly intoxicated.*

**No →** Go to [question **8.8.**](#QFiveDotEight)

**Yes →** Answer the following question.

**8.7.a.** Rationale. Provide the rationale for including this population.

| Click or tap here to enter text. |
| --- |

**8.7.b**. Capacity for consent/decision making capacity. Describe the process that will be used to determine whether a cognitively impaired individual is capable of consent decision making with respect to the research protocol and setting.

| Click or tap here to enter text. |
| --- |

**8.7.b.1.** If there will be repeated interactions with the impaired subjects over a time period when cognitive capacity could increase of diminish, also describe how (if at all) decision-making capacity will be re-assessed and (if appropriate) consent obtained during that time.

| Click or tap here to enter text. |
| --- |

**8.7.c**. Permission (surrogate consent). If the research will include adults who cannot consent for themselves, describe the process for obtaining permission (“surrogate-consent”) from a legally authorized representative (LAR).

*For research conducted in Washington State, see* [***GUIDANCE Consent*** *Diminished and Fluctuating Consent Capacity and Use of a Legally Authorized Representative (LAR)*](https://www.washington.edu/research/hsd/guidance/consent/#7) *to learn which individuals meet the state definition of “legally authorized representative”.*

| Click or tap here to enter text. |
| --- |

**8.7.d.** Assent. Describe whether assent will be required of all, some, or none of the subjects. If some, indicate which subjects will be required to assent and which will not (and why not). Describe any process that will be used to obtain and document assent from the subjects.

| Click or tap here to enter text. |
| --- |

**8.7.e.** Dissent or resistance. Describe how a subject’s objection or resistance to participation (including non-verbal) during the research will be identified, and what will occur in response.

| Click or tap here to enter text. |
| --- |

**8.8.** **Research use of human fetal tissue obtained from elective abortion**. Federal and UW Policy specify some requirements for the consent process. If you are conducting this type of research, check the boxes to confirm these requirements will be followed. N/A

Informed consent for the donation of fetal tissue for research use will be obtained by someone other than the person who obtained the informed consent for abortion.

Informed consent for the donation of fetal tissue for research use will be obtained after the informed consent for abortion.

Participation in the research will not affect the method of abortion.

No enticements, benefits or financial incentives will be used at any level of the process to incentivize abortion or the donation of human fetal tissue.

The informed consent form for the donation of fetal tissue for use in research will be signed by both the woman and the person who obtains the informed consent.

**8.9. Consent-related materials**. Upload to ***Zipline*** all consent scripts/talking points, consent forms, debriefing statements, Information Statements, Short Form consent forms, parental permission forms, and any other consent related materials that will be used. Materials that will be used by a specific site should be uploaded to that site’s Local Site Documents page.

- *Translations must be submitted and approved before they can be used. However, we strongly encourage you to wait to provide them until the IRB has approved the English versions.*
- *Combination forms: it may be appropriate to combine parental permission with consent if parents are subjects as well as providing permission for the participation of their children. Similarly, a consent form may be appropriately considered an assent form for older children.*
- *For materials that cannot be uploaded: upload screenshots or written descriptions that are sufficient to enable the IRB to understand the types of data that will be collected and the nature of the experience for the participants. URLs (website addresses) may also be provided, or written descriptions of websites. Examples of materials that usually cannot be uploaded: mobile apps; computer-administered text; licensed and restricted standardized tests.*

9. PRIVACY AND CONFIDENTIALITY

**9.1. [DETERMINATION]**  **Privacy protections.** Describe the steps that will be taken, if any, to address possible privacy concerns of subjects and potential subjects.

*Privacy refers to the sense of being in control of access that others have to ourselves. This can be an issue with respect to recruiting, consenting, sensitivity of the data being collected, and the method of data collection.*

*Examples:*

- *Many subjects will feel a violation of privacy if they receive a letter asking them to participate in a study because they have ____ medical condition, when their name, contact information, and medical condition were drawn from medical records without their consent. Example: the IRB expects that* [*“cold contact” recruitment letters*](https://www.washington.edu/research/hsd/guidance/recruitment/cold-contact-recruitment/) *will inform the subject about how their information was obtained.*
- *Recruiting subjects immediately prior to a sensitive or invasive procedure (e.g., in an outpatient surgery waiting room) will feel like an invasion of privacy to some individuals.*
- *Asking subjects about sensitive topics (e.g., details about sexual behavior) may feel like an invasion of privacy to some individuals.*

| Participants will be made aware of risk of breach of confidentiality despite all the measures the study will take during the consenting process. There will be risk of others still knowing that they are taking part in the study. The study will ensure all data is kept private and confidential in the following ways:  All in-person data collection (questionnaire and interviews) will be conducted in a private area. All study materials will be stored in a password-protected folder on a secure server maintained by the Kenyatta National Hospital (KNH) with back-up at the end of each day. All datasets will be subject to data standards such as documentation of methods of collection through codebooks, SOPs for data collection, and limitations associated with data collection. Data containing participant identifiers will not be shared, but will be preserved for six years after termination of the study, per institutional policy. Questionnaire data will be collected and stored electronically in HIPAA-compliant, secure, password-protected encrypted REDCap databases. These databases will be managed by the PIs and study coordinator. |
| --- |

**9.2. [DETERMINATION]**  **Identification of individuals in publications and presentations.** Will potentially identifiable information about subjects be used in publications and presentations, or is it possible that individual identities could be inferred from what is planned to be published or presented?

**No**

**Yes →** Will subject consent be obtained for this use?

**Yes**

**No →** Describe the steps that will be taken to protect subjects (or small groups of subjects) from being identifiable.

| Click or tap here to enter text. |
| --- |

**9.3. [DETERMINATION]**  **State mandatory reporting**. Each state has reporting laws that require some types of individuals to report some kinds of abuse, and medical conditions that are under public health surveillance. These include:

- Child abuse
- Abuse, abandonment, neglect, or financial exploitation of a vulnerable adult
- Sexual assault
- Serious physical assault
- Medical conditions subject to mandatory reporting (notification) for public health surveillance

Are you or a member of the research team likely to learn of any of the above events or circumstances while conducting the research **AND** feel obligated to report it to state authorities?

**No**

**Yes →** The UW IRB expects subjects to be informed of this possibility in the consent form or during the consent process, unless you provide a rationale for not doing so:

| This information is part of the information given during consenting |
| --- |

**9.4. [DETERMINATION]**  **Records retention requirements.** Check the box below to indicate assurance that any identifiers (or links between identifiers and data/specimens) and data that are part of the research records will not be destroyed until after the end of the applicable records retention requirements (e.g., Washington State; funding agency or sponsor; Food and Drug Administration). If it is important to say something about destruction of identifiers (or links to identifiers) in the consent form, state that identifying information will be destroyed at the end of the study or after the records retention period required by state and/or federal law.

*See the “Research Data” and “Personal Identifiers” sections of the following website for UW Records management for the Washington State research records retention schedules that apply in general to the UW (not involving UW Medicine data):* [***http://f2.washington.edu/fm/recmgt/gs/research?title=R***](http://f2.washington.edu/fm/recmgt/gs/research?title=R)***;*** ***<https://finance.uw.edu/recmgt/gs/research?title=P>.***

*See the “Research Records and Data” information in Section 8 of this document for the retention schedules for UW Medicine Records:* [***https://www.uwmedicine.org/recordsmanagementuwm-records-retention-schedule.pdf***](https://www.uwmedicine.org/recordsmanagementuwm-records-retention-schedule.pdf)

**Confirm**

**9.5. [DETERMINATION]**  **Certificates of Confidentiality.** Will a federal Certificate of Confidentiality be obtained for the research data? *NOTE: Answer “No” if the study is funded by NIH or the CDC, because most NIH-funded and CDC-funded studies automatically have a Certificate.*

**No**

**Yes**

**9.6. [DETERMINATION]**  **Data and specimen security protections**. Identify the data classifications and the security protections that will be provided for all sites where data will be collected, transmitted, or stored, referring to the [**GUIDANCE Data and Security Protections**](https://www.washington.edu/research/policies/guidance-data-security-protections/) for the minimum requirements for each data classification level. ***It is not possible to answer this question without reading this document. Data security protections should not conflict with records retention requirements.***

**9.6.a.** Choose the level(s) of protections that will be applied to the data and specimens. If more than one level will be used, use the text box to describe which level will apply to which data and which specimens and at which sites.

**Level 1:** Very low risk of harm if disclosed

**Level 2:** Some risk of minor harm if disclosed

**Level 3:** Could cause risk of material harm if disclosed

**Level 4:** Would likely cause serious harm to individuals if disclosed

**Level 5:** Extremely sensitive; could cause severe harm to individuals if disclosed

|  |
| --- |

**9.6.b.** Use this space to provide additional information, details, or to describe protections that do not fit into one of the levels. **HSD allows researchers to request exceptions to data security requirements, if the exception is necessary and does not significantly increase risk to participants**. If there are any protections within the level listed in 9.6.a which will not be followed, list those here, including identifying the sites where this exception will apply. For example, if you intend to store subject identifiers with study data (not permitted under requirement U9 for Risk Levels 3-5), then indicate this in the box below (e.g., “We will not adhere to requirement U9 for screening data”).

| Click or tap here to enter text. |
| --- |

10. RISK / BENEFIT ASSESSMENT

**10.1. [DETERMINATION]**  **Anticipated risks.** Describe the anticipated risks of harm, discomforts, and hazards to the subjects and others of the research procedures.

For each harm, discomfort, or hazard:

- Describe the magnitude, probability, duration, and/or reversibility of the harm, discomfort, or hazard, AND
- Describe how the risks will be reduced or managed. Do not describe data security protections here, these are already described in [question **9.6**.](#QNineDotSix)
- *Consider possible physical, psychological, social, legal, and economic harms, including possible negative effects on financial standing, employability, insurability, educational advancement, or reputation. For example, a breach of confidentiality might have these effects.*
- *Examples of “others”: embryo, fetus, or nursing child; family members; a specific group.*
- *Ensure applicable risk information from any Investigator Brochures, Drug Package Inserts, and/or Device Manuals is included in your description.*
- *Do not include the risks of non-research procedures that are already being performed.*
- *If the study design specifies that subjects will be assigned to a specific condition or intervention, then the condition or intervention is a research procedure - even if it is a standard of care.*
- *Examples of mitigation strategies: inclusion/exclusion criteria; taking blood samples to monitor something that indicates drug toxicity.*
- *Review* [***GUIDANCE Consent*** *Identifying and Describing Reasonably Foreseeable Risks in Research*](https://www.washington.edu/research/hsd/guidance/consent/#r) *for information about which risks should be described in the consent process/form.*
- *As with all questions on this application, you may refer to uploaded documents.*

| We do not anticipate any physical risks due to this study. Women participating in the RCT may experience psychological distress due to interviews about sensitive mental health experiences. There is also a risk of breach of confidentiality and disclosure of information to persons other than the participant. Healthcare professionals are at risk of inducement by their employer to respond to questions in favourable ways. |
| --- |

**10.2. [DETERMINATION]**  **Reproductive risks**. Are there any risks of the study procedures to subjects or partner of subjects related to pregnancy, fertility, lactation or effects on a fetus or neonate?

*Examples: direct teratogenic effects; possible germline effects; effects on fertility; effects on a woman’s ability to continue a pregnancy; effects on future pregnancies.*

**No →** Go to [question **10.3**.](#QTenDotThree)

**Yes →** Answer the following questions:

**10.2.a. Risks.** Describe the magnitude, probability, duration and/or reversibility of the risks.

| Click or tap here to enter text. |
| --- |

**10.2.b. Steps to minimize risk.** Describe the specific steps that will be taken to minimize the magnitude, probability or duration of these risks.

*Examples: inform the subjects about the risks and how to minimize them; require a pregnancy test before and during the study; require subjects to use contraception; advise subjects about banking of sperm and ova.*

*If the use of contraception will be required, describe the allowable methods and the time period when contraception must be used.*

| Click or tap here to enter text. |
| --- |

**10.2.c. Pregnancy**. Describe what will be done if a subject (or a subject’s partner) becomes pregnant.

*For example; will subjects be required to immediately notify study staff, so that the study procedures can be discontinued or modified, or for a discussion of risks, and/or referrals or counseling?*

| Click or tap here to enter text. |
| --- |

**10.3. [DETERMINATION]**  **MRI risk management**. A rare but serious adverse reaction called nephrogenic systemic fibrosis (NSF) has been observed in individuals with kidney disease who received gadolinium-based contrast agents (GBCAs) for the scans. Also, a few healthy individuals have a severe allergic reaction to GBCAs.

**NA**

**10.3.a. Use of gadolinium**. Will any of the MRI scans involve the use of a gadolinium-based contrast agent (GBCA)?

**No**

**Yes →** Which agents will be used? *Check all that apply.*

| **Check all that apply** | **Brand Name** | **Generic Name** | **Chemical Structure** | |
| --- | --- | --- | --- | --- |
|  | Dotarem | Gadoterate meglumine | Macrocylic | |
|  | Eovist / Primovist | Gadoxetate disodium | Linear | |
|  | Gadavist | Gadobutro | Macrocyclic | |
|  | Magnevist | Gadpentetate dimeglumine | Linear | |
|  | MultiHance | Gadobenate dimeglumine | Linear | |
|  | Omniscan | Gadodiamide | Linear | |
|  | OptiMARK | Gadoversetamide | Linear | |
|  | ProHance | Gadoteridol | Macrocyclic | |
|  | Other, provide name: |  |  | |
| Click or tap here to enter text. | | |  |  |

**10.3.a.1.** The FDA has concluded that gadolinium is retained in the body and brain for a significantly longer time than previously recognized, especially for linear GBCAs. The health-related risks of this longer retention are not yet clearly established. However, the UW IRB expects researchers to provide a compelling justification for using a linear GBCA instead of a macrocyclic GBCA, to manage the risks associated with GBCAs.

Describe why it is important to use a GBCA with the MRI scan(s). Describe the dose that will be used and (if it is more than the standard clinical dose recommended by the manufacturer) why it is necessary to use a higher dose. If a linear GBCA will be used, explain why a macrocyclic GBCA cannot be used.

| Click or tap here to enter text. |
| --- |

**10.3.a.2.** Information for subjects. Confirm by checking this box that subjects will be provided with the FDA-approved Patient Medication Guide for the GBCA being used in the research or that the same information will be inserted into the consent form.

**Confirmed**

**10.3.b.** Who will (1) calculate the dose of GBCA; (2) prepare it for injection; (3) insert and remove the IV catheter; (4) administer the GBCA; and (5) monitor for any adverse effects of the GCBA? Also, what are the qualifications and training of these individual(s)?

| Click or tap here to enter text. |
| --- |

**10.3.c.** Describe how the renal function of subjects will be assessed prior to MRI scans and how that information will be used to exclude subjects at risk for NSF.

| Click or tap here to enter text. |
| --- |

**10.3.d.** Describe the protocol for handling a severe allergic reaction to the GBCA or any other medical event/emergency during the MRI scan, including who will be responsible for which actions.

| Click or tap here to enter text. |
| --- |

**10.4. [DETERMINATION]**  **Unforeseeable risks**. Are there any research procedures that may have risks that are currently unforeseeable?

*Example: using a drug that hasn’t been used before in this subject population.*

**No**

**Yes** **→** Identify the procedures.

| Click or tap here to enter text. |
| --- |

**10.5. Subjects who will be under regional or general anesthesia.** Will any research procedures occur while patients are under general or regional anesthesia, or during the 3 hours preceding general or regional anesthesia (supplied for non-research reasons)?

**No**

**Yes** **→** Check all the boxes that apply.

Administration of any drug for research purposes

Inserting an intra-venous (central or peripheral) or intra-arterial line for research purposes

Obtaining samples of blood, urine, bone marrow or cerebrospinal fluid for research purposes

Obtaining a research sample from tissue or organs that would not otherwise be removed during surgery.

Administration of a radio-isotope for research purposes**

Implantation of an experimental device

Other manipulations or procedures performed solely for research purposes (e.g., experimental liver dialysis, experimental brain stimulation)

If any of the boxes are checked:

Provide the name and institutional affiliation of a physician anesthesiologist who is a member of the research team or who will serve as a safety consultant about the interactions between the research procedures and the general or regional anesthesia of the subject-patients. If the procedures will be performed at a UW Medicine facility or affiliate, the anesthesiologist must be a UW faculty member, and the Vice Chair of Clinical Research in the UW Department of Anesthesiology and Pain Medicine must be consulted in advance for feasibility, safety and billing.

| Click or tap here to enter text. |
| --- |

*** If the box about radio-isotopes is checked, the study team is responsible for informing in advance all appropriate clinical personnel (e.g., nurses, technicians, anesthesiologists, surgeons) about the administration and use of the radio-isotope, to ensure that any personal safety issues (e.g., pregnancy) can be appropriately addressed. This is a condition of IRB approval.*

**10.6. Data and Safety Monitoring**. A Data and Safety Monitoring Plan (DSMP) is required for clinical trials (as defined by NIH). If required for this research, or if there is a DSMP for the research regardless of whether it is required, upload the DSMP to ***Zipline***. If it is embedded in another document being uploading (for example, a Study Protocol) use the text box below to name the document that has the DSMP. Alternatively, provide a description of the DSMP in the text box below. For guidance on developing a DSMP, see the [ITHS webpage on Data and Safety Monitoring Plans](https://www.iths.org/investigators/services/dsm/data-and-safety-monitoring-plans/).

| DSMP will be uploaded |
| --- |

**10.7. Un-blinding**. If this is a double-blinded or single-blinded study in which the participant and/or relevant study team members do not know the group to which the participant is assigned, describe the circumstances under which un-blinding would be necessary, and to whom the un-blinded information would be provided.

| This trial is not blinded |
| --- |

**10.8. Withdrawal of participants**. If applicable, describe the anticipated circumstances under which participants will be withdrawn from the research without their consent. Also, describe any procedures for orderly withdrawal of a participant, regardless of the reason, including whether it will involve partial withdrawal from procedures and any intervention but continued data collection or long-term follow-up.

| Participants will be made aware of their right to withdraw from the study at any time without consequences. |
| --- |

**10.9. [DETERMINATION]**  **Anticipated direct benefits to participants**. If there are any direct research-related benefits that some or all individual participants are likely to experience from taking part in the research, describe them below:

*Do not include benefits to society or others, and do not include subject payment (if any). Examples: medical benefits such as laboratory tests (if subjects receive the results); psychological resources made available to participants; training or education that is provided.*

| All participants will be screened for PMAD using the PHQ2 tool and provided with psychosocial support regardless of the PHQ2 score. Participants in the intervention arm will receive PM+ and, if necessary, telepsychiatry, which are both evidence-based interventions to alleviate depression and anxiety symptoms. There are also possible benefits from regular interactions with the healthcare workers to answer mental health-related questions and concerns. |
| --- |

**10.10. [DETERMINATION]**  **Return of individual research results.**

*In this section, provide your plans for the return of individual results. An “individual research result” is any information collected, generated or discovered in the course of a research study that is linked to the identity of a research participant. These may be results from screening procedures, results that are actively sought for purposes of the study, results that are discovered unintentionally, or after analysis of the collected data and/or results has been completed.*

*See the* [***GUIDANCE Return of Individual Results***](https://www.washington.edu/research/hsd/guidance/results/) *for information about results that should and should not be returned, validity of results, the Clinical Laboratory Improvement Amendment (CLIA), consent requirements and communicating results.*

**10.10.a.** Is it anticipated that the research will produce any individual research results that are clinically actionable?

*“Clinically actionable” means that there are established therapeutic or preventive interventions or other available actions that have the potential to change the clinical course of the disease/condition or lead to an improved health outcome.*

*In general, every effort should be made to offer results that are clinically actionable, valid and pose life-threatening or severe health consequences if not treated or addressed quickly. Other clinically actionable results should be offered if this can be accomplished without compromising the research.*

**No**

**Yes** **→** Answer the following questions (**10.10.a.1** through **10.10.a.3.**)

**10.10.a.1.** Describe the clinically actionable results that are anticipated and explain which results, if any, could be urgent (i.e., because they pose life-threatening or severe health consequences if not treated or addressed quickly).

*Examples of urgent results include very high calcium levels, highly elevated liver function test results, positive results for reportable STDs.*

| Click or tap here to enter text. |
| --- |

**10.10.a.2**. Explain which of these results will be offered to subjects.

| Click or tap here to enter text. |
| --- |

**10.10.a.3**. Explain which results will not be offered to subjects and provide the rationale for not offering these results.

*Reasons not to offer the results might include:*

- *There are serious questions regarding validity or reliability*
- *Returning the results has the potential to cause bias*
- *There are insufficient resources to communicate the results effectively and appropriately*
- *Knowledge of the result could cause psychosocial harm to subjects*

| Click or tap here to enter text. |
| --- |

**10.10.b.** Is there a plan for offering subjects any results that are not clinically actionable?

*Examples: non-actionable genetic results, clinical tests in the normal range, experimental and/or uncertain results.*

**No**

**Yes** **→** Explain which results will be offered to subjects and provide the rationale for offering these results.

| Click or tap here to enter text. |
| --- |

**10.10.c.** Describe the validity and reliability of any results that will be offered to subjects.

*The IRB will consider evidence of validity such as studies demonstrating diagnostic, prognostic, or predictive value, use of confirmatory testing, and quality management systems.*

| Click or tap here to enter text. |
| --- |

**10.10.d.** Describe the process for communicating results to subjects and facilitating understanding of the results. In the description, include who will approach the participant with regard to the offer of results, who will communicate the result (if different), the circumstances, timing, and communication methods that will be used.

| Click or tap here to enter text. |
| --- |

**10.10.e.** Describe any plans to share results with family members (e.g., in the event a subject becomes incapacitated or deceased).

| Click or tap here to enter text. |
| --- |

**10.10.f.** Check the box to indicate that any plans for return of individual research results have been described in the consent document. If there are no plans to provide results to participants, this should be stated in the consent form.

*See the* [*GUIDANCE Return of Individual Results*](https://www.washington.edu/research/hsd/guidance/results/) *for information about consent requirements.*

**Confirmed**

**10.11. Commercial products or patents**. Is it possible that a commercial product or patent could result from this study?

**No**

**Yes →** Describe whether subjects might receive any remuneration/compensation and, if yes, how the amount will be determined.

| Click or tap here to enter text. |
| --- |

11. ECONOMIC BURDEN TO PARTICIPANTS

**11.1. Financial responsibility for research-related injuries**. Answer this question only if the lead researcher is not a UW student, staff member, or faculty member whose primary paid appointment is at the UW.

For each institution involved in conducting the research: Describe who will be financially responsible for research-related injuries experienced by subjects, and any limitations. Describe the process (if any) by which participants may obtain treatment/compensation.

| NA-UW affiliated |
| --- |

**11.2. Costs to subjects**. Will there be any research-related costs for which subjects and/or their health insurance may be responsible (examples might include: CT scan required for research eligibility screening; co-pays; surgical costs when a subject is randomized to a specific procedure; cost of a device; travel and parking expenses that will not be reimbursed)?

**No**

**Yes →** Provide a description of the costs that may be incurred.

| Click or tap here to enter text. |
| --- |

12. RESOURCES

**12.1. [DETERMINATION]**  **Faculty Advisor**. (For researchers who are students or residents.) Provide the following information about the faculty advisor.

- Advisor’s name
- Your relationship with your advisor (for example: graduate advisor; course instructor)
- Your plans for communication/consultation with your advisor about progress, problems, and changes.

| NA |
| --- |

**12.2. UW Principal Investigator Qualifications**. Upload a current or recent Curriculum Vitae (CV), Biosketch (as provided to federal funding agencies), or similar document to the Local Site Documents page in ***Zipline***. The purpose of this is to address the PI’s qualifications to conduct the proposed research (education, experience, training, certifications, etc.).

*For help with creating a CV, see* [*http://adai.uw.edu/grants/nsf_biosketch_template.pdf*](http://adai.uw.edu/grants/nsf_biosketch_template.pdf) *and* [*https://intranet.medicine.uw.edu/academic-hr/curriculum-vitae-cv*](https://intranet.medicine.uw.edu/academic-hr/curriculum-vitae-cv)

**The CV will be uploaded.**

**12.3. UW Study team qualifications**. Describe the qualifications and/or training for each UW study team member to fulfill their role on the study and perform study procedures. (You may be asked about non-UW study team members during the review; they should not be described here.) You may list these individuals by name, however if you list an individual by name, you will need to modify this application if that individual is replaced. Alternatively, you can describe study roles and the qualifications and training the PI or study leadership will require for any individual who might fill that role. The IRB will use this information to assess whether risks to subjects are minimized because study activities are being conducted by properly qualified and trained individuals.

**Describe: The role (or name of person), the study activities they will perform, and the qualifications or training that are relevant to performing those study activities.**

| PRINCIPAL INVESTIGATORS  Responsible for providing leadership on all aspects of the project including study protocol, implementation analyses and results, administration, regulatory requirements, standard operating procedures and oversee the study teams. Extensive experience in leading collaborative research including clinical trials.  IMPLEMENTATION SCIENTISTS  Responsible for overseeing the implementation science aspects of the project. Experience in Implementation Science in LMIC  BIOSTATISTICIAN AND STUDY ANALYSTS  Responsible for overseeing analysis of results. Experienced biostatistician and research analysts.  STUDY CO-ORDINATORS  Responsible for protocol submission, staff training, coordination. Experience in coordination.  PSYCHIATRISTS  Responsible for oversight of the clinical management of participants. Trained psychiatrists.  PROGRAM MANAGERS  Responsible for ensuring smooth day to day running of study activities. Experience in managing collaborative studies |
| --- |

**12.4. Study team training and communication.** Describe how it will be ensured that each study team member is adequately trained and informed about the research procedures and requirements (including any changes) as well as their research-related duties and functions.

There is no study team

| All study staff will be trained prior to start of study activities. Dr. John Kinuthia (PI) will supervise training of clinical personnel and study staff in study procedures.  The study team will communicate through weekly Zoom calls. |
| --- |

13. OTHER APPROVALS, PERMISSIONS, AND REGULATORY ISSUES

**13.1. [DETERMINATION]** **Approvals and permissions**. Identify any other approvals or permissions that will be obtained. For example: from a school, external site/organization, funding agency, employee union, UW Medicine clinical unit.

*Do not attach the approvals and permissions unless requested by the IRB.*

| Kenyatta National Hospital- University of Nairobi Ethics Review Committee Approval (KNH-UoN ERC) |
| --- |

**13.2. Financial Conflict of Interest**. Does any UW member of the team have ownership or other Significant Financial Interest (SFI) with this research as defined by [UW policy GIM 10?](https://www.washington.edu/research/policies/gim-10/)

**No**

**Yes** **→** Has the Office of Research made a determination regarding this SFI as it pertains to the proposed research?

**No** **→** Contact the Office of Research (206.616.0804, [research@uw.edu](mailto:research@uw.edu)) for guidance on how to obtain the determination.

**Yes** **→** Upload the Conflict Management Plan for every UW team member who has a FCOI with respect to the research, to ***Zipline***. If it is not yet available, use the text box to describe whether the Significant Financial Interest has been disclosed already to the UW Office of Research and include the FIDS Disclosure ID if available.

| Click or tap here to enter text. |
| --- |
