# Supplementary material for: Integration of stepped care for perinatal mood and anxiety disorders among women attending maternal and child health clinics in Kenya: Protocol for a cluster randomized controlled trial
Source: PLoS One. 2026 Jun 11;21(6):e0349732. doi: 10.1371/journal.pone.0349732 (PMC13257984; doi:10.1371/journal.pone.0349732)
Supplement: S3 File — (DOCX) [file pone.0349732.s003.docx]

**Integration of stepped care for perinatal mood and anxiety disorders among women attending MCH clinics**

Study Protocol

Principal Investigator: John Kinuthia, Keshet Ronen, Amritha Bhat

**Table of Contents**

[**1)** **TITLE** 4](#_Toc133580527)

[**2)** **INVESTIGATORS (Roles and responsibilities)** 4](#_Toc133580528)

[**3)** **SIGNATURES:** 6](#_Toc133580529)

[**4) COLLABORATING INSTITUTIONS** 7](#_Toc133580530)

[**5) FUNDING AGENCY** 7](#_Toc133580531)

[**6) LIST OF ABBREVIATIONS** 7](#_Toc133580532)

[**7) ABSTRACT** 7](#_Toc133580533)

[**8) BACKGROUND** 8](#_Toc133580534)

[**9) LITERATURE REVIEW** 9](#_Toc133580535)

[**10) RATIONALE** 13](#_Toc133580536)

[**11) HYPOTHESIS, OBJECTIVES, & STUDY QUESTIONS:** 14](#_Toc133580537)

[11.1) BROAD OBJECTIVES 14](#_Toc133580538)

[11.2) SPECIFIC OBJECTIVES 14](#_Toc133580539)

[**12) STUDY DESIGN AND METHODOLOGY** 15](#_Toc133580540)

[12.1) STUDY AREA DESCRIPTION: 15](#_Toc133580541)

[12.2) STUDY DESIGN OVERVIEW 15](#_Toc133580542)

[AIM 1: Using participatory design, optimize IPMH and develop implementation strategies. 16](#_Toc133580543)

[**STUDY DESIGN** 16](#_Toc133580544)

[**STUDY POPULATION AND SAMPLE SIZE DETERMINATION** 16](#_Toc133580545)

[**RECRUITMENT, ENROLMENT AND DATA COLLECTION** 17](#_Toc133580546)

[**DATA ANALYSIS** 18](#_Toc133580547)

[AIM 2: Determine the effect of IPMH and implementation strategies on mental health and pregnancy outcomes among perinatal women from pregnancy to 6 months postpartum. 18](#_Toc133580548)

[**STUDY DESIGN** 18](#_Toc133580549)

[**STUDY POPULATION AND SAMPLE SIZE DETERMINATION** 18](#_Toc133580550)

[**RECRUITMENT AND ENROLMENT** 19](#_Toc133580551)

[**RANDOMIZATION** 20](#_Toc133580552)

[**DATA COLLECTION** 20](#_Toc133580553)

[AIM 3: Determine effect of IPMH and its implementation strategies on service delivery and implementation outcomes, and identify multilevel drivers of successful implementation. 21](#_Toc133580554)

[**STUDY DESIGN** 21](#_Toc133580555)

[Aim 3a: Determine service delivery and implementation outcomes. 21](#_Toc133580556)

[**STUDY POPULATION** 22](#_Toc133580557)

[**DATA COLLECTION** 23](#_Toc133580558)

[Aim 3b: Identify multilevel drivers of implementation success. 24](#_Toc133580559)

[**STUDY POPULATION** 24](#_Toc133580560)

[**DATA COLLECTION** 24](#_Toc133580561)

[**OUTCOMES** 24](#_Toc133580562)

[**AIM 1** 24](#_Toc133580563)

[**AIM 2** 25](#_Toc133580564)

[**AIM 3** 26](#_Toc133580565)

[**13) TRAINING PROCEDURES** 26](#_Toc133580566)

[**14) QUALITY ASSURANCE PROCEDURES** 26](#_Toc133580567)

[**15) ETHICAL CONSIDERATIONS** 27](#_Toc133580568)

[15.1 ASSESSMENT OF RISKS AND BENEFITS 27](#_Toc133580569)

[**POTENTIAL RISKS** 27](#_Toc133580570)

[**PROTECTION AGAINST RISK** 27](#_Toc133580571)

[**POTENTIAL BENEFITS OF PROPOSED RESEARCH TO THE SUBJECTS AND OTHERS** 28](#_Toc133580572)

[**IMPORTANCE OF THE KNOWLEDGE GAINED** 28](#_Toc133580573)

[**16) DATA MANAGEMENT AND SHARING PLAN** 29](#_Toc133580574)

[ELEMENT 1: DATA TYPE 29](#_Toc133580575)

[ELEMENT 2: RELATED TOOLS, SOFTWARE AND/OR CODE 30](#_Toc133580576)

[ELEMENT 3: STANDARDS 30](#_Toc133580577)

[ELEMENT 4: DATA PRESERVATION, ACCESS, AND ASSOCIATED TIMELINES 30](#_Toc133580578)

[ELEMENT 5: ACCESS, DISTRIBUTION, OR REUSE CONSIDERATIONS 30](#_Toc133580579)

[ELEMENT 6: OVERSIGHT OF DATA MANAGEMENT AND SHARING 31](#_Toc133580580)

[**17) STUDY LIMITATIONS AND HOW TO MINIMIZE THEM:** 31](#_Toc133580581)

[**18) DISSEMINATION PLAN** 32](#_Toc133580582)

[**19) TIMELINE/ TIME FRAME:** 33](#_Toc133580583)

[**20) HUMAN SUBJECTS** 34](#_Toc133580584)

[**21) REFERENCES** 36](#_Toc133580585)

# **TITLE**

Integration of stepped care for perinatal mood and anxiety disorders among women attending MCH in Kenya

# **INVESTIGATORS (Roles and responsibilities)**

John Kinuthia, MBChB, MMed, MPH (PRINCIPAL INVESTIGATOR)

(Responsible for providing leadership on all aspects of the project, administration, regulatory requirements and will oversee the study team in Kenya.)

Head, Department of Research & Programs, Kenyatta National Hospital

PO Box 20723-00202, Nairobi, Kenya

Tel: +254 0722 799-052

Email: [kinuthia@uw.edu](mailto:kinuthia@uw.edu)

Keshet Ronen, MPH, PhD (CO- PRINCIPAL INVESTIGATOR)

(Responsible for providing leadership of study protocol, study design and analyses

Assistant Professor, Department of Global Health

Harborview Medical Center, 325 Ninth Ave., Box 359909, Seattle, WA 98104

Tel: +1-206-543-4278

Fax: +1-206-543-4818

Email: [keshet@uw.edu](mailto:keshet@uw.edu)

Amritha Bhat, MBBS, MD, MPH (CO- PRINCIPAL INVESTIGATOR)

(Responsible for providing leadership of the overall science of the project, development of standard operating procedures, data collection tools, data analysis and interpretation of study results)

Assistant Professor, Department of Global Health

Harborview Medical Center, 325 Ninth Ave., Box 359909, Seattle, WA 98104

Tel: +1-206-543-3117

Email: [amritha@uw.edu](mailto:amritha@uw.edu)

Anjuli Wagner, PhD, MPH (CO-INVESTIGATOR)

(Responsible for overseeing the implementation science aspects of the project)

Acting Assistant Professor, Department of Global Health, University of Washington

Harborview Medical Center, 325 Ninth Ave., Box 359909, Seattle, WA 98104

Tel: +1-978-460-2331

Email: [anjuliw@uw.edu](mailto:anjuliw@uw.edu)

Barbra Richardson, PhD (BIOSTATISTICIAN)

(Responsible for overseeing analysis of results)

Research Professor, Biostatistics and Global Health, University of Washington

Hans Rosling Center, 398 15^th^ Ave NE, Box 351620, Seattle WA, 98195

Tel: +1-206-550-7540

Email: [barbrar@uw.edu](mailto:barbrar@uw.edu)

Bryan Weiner, PhD (CO-INVESTIGATOR)

(Responsible for providing methodologic leadership on implementation science)

Professor, Departments of Health Services and Global Health

University of Washington, Box 357965, Seattle WA, 98104

Tel: +1 206-282-7882

Email: [bjweiner@uw.edu](mailto:bjweiner@uw.edu)

Carol Levin, PhD (CO-INVESTIGATOR)

(Responsible for providing methodologic leadership on implementation science)

Professor, Departments of Health Services and Global Health

University of Washington, Box 357965, Seattle WA, 98104

Tel: +1 206-685-7302

Email: [klevin@uw.edu](mailto:klevin@uw.edu)

Nancy Ngumbau MBChB, MPH (CO-INVESTIGATOR)

(Responsible for the site protocol development, data analysis and staff oversight)

Research Scientist, Kenyatta National Hospital

Nairobi, Kenya

Tel: +254 0713 917 226

Email: nancym390@gmail.com

Linnet Ongeri MBChB, MMed, MPH (CO-INVESTIGATOR)

(Responsible for advisement on telepsychiatry, management of study staff and project evaluation)

Psychiatrist and Research Scientist, Kenya Medical Research Institute (KEMRI)

Nairobi, Kenya

Tel: +254-722-615-999

Email: [linongeri@gmail.com](mailto:linongeri@gmail.com)

Agnes Karingo, MBChB, MPH (CO-INVESTIGATOR)

(Responsible for overseeing study coordination, ERC/IRB communication, protocol development and analysis)

Research Scientist, Kenyatta National Hospital

Nairobi, Kenya

Tel: +254 0715-181-283

Email: [karumeagnes@gmail.com](mailto:karumeagnes@gmail.com)

Anna Larsen PhD (CO-INVESTIGATOR)

(Responsible for the development of standard operating procedures, data collection tools and data analysis)

Post-doctoral Research Scientist, Department of Epidemiology

University of Washington, Box 357965, Seattle WA, 98104

Email: annalar@uw.edu

Julia Dettinger, MPH (RESEARCH SCIENTIST)

(Responsible for overseeing study coordination, ERC/IRB communication, protocol development and analysis)

Department of Global Health and Nursing, University of Washington

Hans Rosling Center, 398 15^th^ Ave NE, Box 351620, Seattle WA, 98195

Tel: +1-206-221-1041

Fax: +1-206-744-3693

Email: [jcdettin@uw.edu](mailto:jcdettin@uw.edu)

Asterico Neema, MPH

(Responsible for coordinating and overseeing day to day research activities at the sites)

Research Scientist, Kenyatta National Hospital

Tel: +254714242437

Email: [astericoneema@gmail.com](mailto:astericoneema@gmail.com)

David Owaga

(Responsible for REDCAP, data management, analysis and data quality checks)

Kenyatta National Hospital

Tel: +254790179268

Email: owaga2007@gmail.com

Yuwei Wang, PhDc(Research Scientist)

(Research assistant responsible for project data management and overall implementation support)

Hans Rosling Center, 398 15th Ave NE, Box 351620, Seattle WA, 98195

Email: [yuwei97@uw.edu](mailto:yuwei97@uw.edu)

Lincoln Pothan (Research Scientist)

(Research coordinator, Global WACh responsible for standard operating procedures and coordination support)

Hans Rosling Center, 398 15th Ave NE, Box 351620, Seattle WA, 98195

Email: [lpothan@uw.edu](mailto:lpothan@uw.edu)

# **3) COLLABORATING INSTITUTIONS**

Kenyatta National Hospital, Nairobi, Kenya

University of Washington,

Kenya Medical Research Institute

# **4) FUNDING AGENCY**

Funding type: Grant

Name of Funding agency: National Institute of Mental Health

Principal Investigator on Proposal: John Kinuthia, Keshet Ronen, Amritha Bhat

Title of Proposal: Integration of stepped care for perinatal mood and anxiety disorders among women attending MCH in Kenya

Dates: 04/01/2023-31/03/2028

# **5) LIST OF ABBREVIATIONS**

ANC Antenatal care

ART Antiretroviral therapy

CBT Cognitive Behavior Therapy

CFIR Consolidated Framework for Implementation Research

DALYs Disability Adjusted Life Years

EBIs Evidence-based interventions

eSOC Enhanced Standard of Care

GAD Generalized Anxiety Disorder

GEE Generalized Estimating Equations

HCW Healthcare worker

HIC High Income Countries

IOF Proctor’s Implementation Outcomes Framework

IPMH Integrated Perinatal Mental Health program

LMICs Low- and middle-income countries

MCH Maternal Child Health

mhGAP Mental Health Gap Action Programme

NCD Noncommunicable disease

PDG Participatory Design Group

PHQ Patient Health Questionnaire

PMAD Perinatal mood and anxiety disorders

PM+ Problem Management Plus

PMTCT Prevention of Mother To Child HIV Transmission

RCT Randomized Clinical trial

WHO World Health Organization

WHOQOL-BREF World Health Organization Quality of Life Brief Version

WLWH Women Living with HIV

# **6) ABSTRACT**

**Background:** Globally, perinatal mood and anxiety disorders (PMAD) impacts 10-20% of perinatal women, with a pooled prevalence of depression at 11.9% and anxiety at 15.2%. Most of these cases go undetected and untreated since worldwide under 30% of those requiring mental health services have access to them. The Kenya Mental Health Action Plan 2021-2025 highlights a goal of expanding access to mental health services including in MCH clinics. In Kenya, over 95% of all pregnant women receive at least one antenatal care (ANC) service from a skilled provider. However, mental health screening is not currently standardized in antenatal and postnatal care visits in Kenya, representing a missed opportunity to offer mental health services to those with PMAD symptoms. Using mhGAP assessment by non-specialized providers can increase screening in MCH and PMTCT clinics with limited specialized workforce. However, screening alone does not improve PMAD outcomes and needs to be followed by improved access to perinatal mental health treatments.

**Broad objective:** This study seeks to evaluate the effectiveness and implementation outcomes of Integrated Perinatal Mental Health Program (universal mental health screening, problem management plus counseling intervention and tele psychiatry) in a Hybrid Type II trial.

**Study design:** This is a hybrid type II trial that will test a combination of interventions (universal WHO Mental Health Gap Action Programme (mhGAP)-recommended mental health screening, the Problem Management Plus (PM+) counseling intervention for women experiencing PMAD, and telepsychiatry for women with severe symptoms, suicidality or no response to PM+) in a stepped care model among perinatal women, develop implementation strategies to support the model’s integration into routine perinatal care in Kenya and evaluate IPMH’s effectiveness and implementation outcomes. The study will be conducted in clinics in Kisumu, Siaya, and Homa Bay counties of Western Kenya, Kenya and will include Perinatal women, specialist and non-specialist HCWs (HTS and lay providers, facility nurses), and policymakers working in perinatal and mental health at the national and county levels

# **7) BACKGROUND**

Perinatal mood and anxiety disorders (PMAD), defined as depression and anxiety during pregnancy or up to 1 year postpartum, account for substantial morbidity and mortality among birthing people globally. Approximately 10-20% of pregnant and postpartum women experience PMAD, with elevated burden in low- and middle-income countries (LMICs) (>20% vs. 13% in high-income countries)^1,2^. In addition to causing significant disability among perinatal women, PMAD is associated with risks to the infant, including elevated risk of preterm birth, low birth weight and intra uterine growth retardation, pregnancy loss and stillbirth.^5–8^ Kenya, like many LMICs, experiences a severe shortage of specialized mental healthcare workers (HCWs) and poor coverage of screening and treatment for PMAD. It is estimated that over 75% of those who need mental health services do not receive them, and those who do often travel large distances and experience long waits to see specialist providers. The World Health Organization (WHO) and Kenya’s national mental health strategy have prioritized integration of mental health care in primary care settings, with care provision by non-specialist providers, engagement of lay workers, and use of technology to improve intervention reach.

Several evidence-based interventions (EBIs) are recommended by the WHO for identification and management of PMAD by non-specialist providers in resource-limited settings.^9^ However, their implementation in routine care in Kenya is currently limited, due to lack of provider training and comfort with perinatal mental health treatments and poor linkage across cadres of providers (such as primary care providers and mental health providers). Sustainable integration of perinatal mental healthcare in Kenya’s routine perinatal services requires development and testing of tailored interventions that strengthen workforce capacity and facilitate linkage across provider cadres, as well as implementation strategies to facilitate high-quality intervention delivery. We have identified three EBIs to promote perinatal mental health: universal WHO Mental Health Gap Action Programme (mhGAP)-recommended mental health screening, the Problem Management Plus (PM+) counseling intervention for women experiencing PMAD, and telepsychiatry for women with severe symptoms, suicidality or no response to PM+. **We propose to combine these interventions in a stepped care model, named the Integrated Perinatal Mental Health program (IPMH), and develop implementation strategies to support the model’s integration into routine perinatal care in Kenya. We will then evaluate IPMH’s effectiveness and implementation outcomes in a Hybrid Type II trial.**

# **8) LITERATURE REVIEW**

**A1. Mental disorder is the leading cause of disability burden worldwide.** Mental disorders worldwide represent the leading cause of disability measured as Disability Adjusted Life Years (DALYs), accounting for over 10% of global DALYs^1^. They are the second leading cause of death (17% of deaths)^1^. As disease burden globally continues to shift from predominantly communicable to noncommunicable disease (NCD) under the epidemiologic transition^2^, the NCD burden is shifting toward a higher proportion of neuropsychiatric disorders^3–5^. Globally, depressive disorders are among the top three causes of years lived with disability^6^, account for 40% of all mental illness^5^, and affect 350 million people comprising 4% of the population.^7^ Women are disproportionally burdened by depression, with double the lifetime risk than their male counterparts^8,9^. Periods of biological change in women’s lives are characterized by higher risk for depression^4,10^.

**A2. Pregnancy and postpartum are periods of high vulnerability to mental disorder.** More than 1 in 10 women experience a mental disorder during pregnancy and 13% after delivery^11–13^, most commonly depression^12^. Hormonal shifts during these periods of biological change are widely implicated as important causes of mental disorder^13,14^. Despite the prevalence and ubiquity of risk for depression in pregnancy and postpartum, most cases go undetected and untreated since worldwide under 30% of those requiring mental health services have access to them^15^.

Pregnancy in adolescence leads to additive physiologic and psychosocial alterations between the parallel biological changes of teenage development and childbearing, putting pregnant adolescents at high risk for depression^16,17^. Pregnancy and postpartum are critical time periods for the health and survival of mothers and their infants^10,18,19^, yet maternal depression, especially among adolescents and young women, remains underprioritized^4^.

**Women in low- and middle-income countries are disproportionately affected by maternal mental health.** A systematic review and meta-analysis of maternal depression from 47 studies across 17 low- and middle-income countries (LMICs) revealed even higher prevalence of maternal depression than seen in high-income countries (HICs), identifying 15.6% (95% confidence interval (CI): 15.4 – 15.9) prevalence of antenatal depression and 19.8% (95% CI: 19.5 – 20.0) postnatally^20,21^. While the burden of maternal depression in LMICs is high, of further concern are the gaps in evidence – WHO reports that maternal mental health data exist for 90% of HICs yet only 10% of LMICs^22^. Health resources in LMICs are stretched and most have a dearth of mental health professionals and services^20,23^. The annual rate of visits to mental health outpatient facilities in the African Region is 14 visits per 100,000. This is 75-fold lower than the global annual rate of 1051 per 100,000^24^. Consequently, 76-85% of those in LMICs requiring mental health services do not receive intervention^25^. Further, women in LMICs may be at higher risk for perinatal depression due to high lifetime fertility^26^. The ten countries with the highest total fertility (6-8 children per woman) are located in the African Region, thus women in this region spend much of their reproductive years in pregnant or postpartum states – times of high risk for depression^26^.

**A4. Perinatal mood and anxiety disorders are common pregnancy complications among perinatal women and associated with poor maternal and child outcomes**^27^. Globally, PMAD impacts 10-20% of perinatal women^20^, with a pooled prevalence of depression at 11.9% and anxiety at 15.2%.^28^ PMAD prevalence is substantially higher among women from LMICs vs. high income countries (20% vs. 13%)^20,29,30^; a meta-analysis from multiple sub-Saharan African settings found a 26.3% prevalence of depression in pregnancy.^31^ In addition, WLWH are at greater risk of developing depressive and anxiety symptoms in the perinatal period compared to those without HIV.^32^ Among WLWH, multiple studies have established that PMAD are associated with lower adherence to antiretroviral therapy (ART), more so than structural barriers such as income and travel time.^33^ PMAD cause low self-efficacy and low motivation, which can interfere with medication adherence.^34^ The effects of untreated maternal depression and anxiety extend to the baby as well.^35^ Adverse outcomes such as preterm birth, low birth weight, insecure infant attachment, and later child mental health and behavioral problems are associated with PMAD.^36^ Settings with high HIV prevalence and slower maternal child health gains, such as some African settings, are particularly in-need of PMAD services.

**A5. Links between maternal depression, adolescence, and poor neonatal health are well-established.** Associations between a mother’s well-being and her infant’s health have been found across varied contexts.^37–40^ A meta-analysis from 2010 combining data from HICs and LMICs found an association between depression during pregnancy and both preterm birth and low birthweight^40^, while another in 2013 confirmed the influence of maternal depression on preterm birth^39^ (Table 1). Studies have found adolescent pregnancy is associated with low birthweight, preterm birth, and neonatal death^41–43^. While initiatives such as the Grand Challenges for Global Mental Health articulate the need for research in global contexts, ^4,44^, neither meta-analysis includes data from the African Region. One meta-analysis among LMICs including four countries in SSA (Nigeria^45^, Malawi^46^, Ethiopia^47^, and South Africa^48^) points to a relationship between maternal depression (assessed during and after pregnancy) and stunting (pooled OR: 1.4, 95% CI: 1.2 – 1.8).^49^. The neonatal and infant health consequences of maternal depression must be explored longitudinally in SSA.

**Table 1. Association between maternal depression and negative perinatal outcomes**


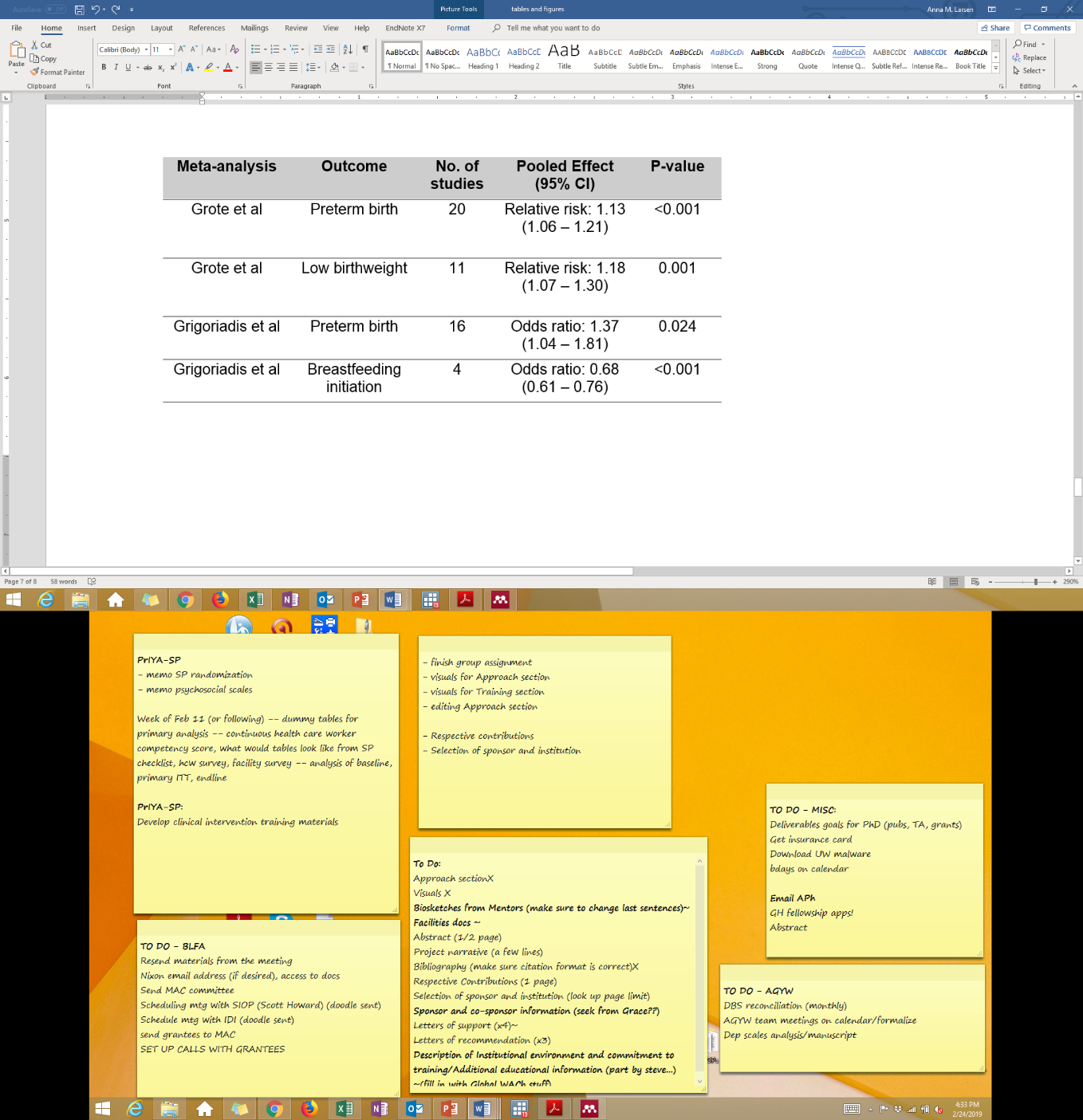


**A6. Improving outcomes in perinatal women begins with identification of those with PMAD by systematic screening.** Expert-led clinical intervention programs, global development agendas, and donor-led initiatives increasingly call for integration of depression screening into routine care settings in LMICs.^4,44,50^ Maternal child health (MCH) care and PMTCT programs are widely attended in sub-Saharan Africa, particularly in Kenya, where over 95% of all pregnant women receive at least one antenatal care (ANC) service from a skilled provider.^51^ This offers an opportunistic access point for PMAD screening. The Kenya Mental Health Action Plan 2021-2025 highlights a goal of expanding access to mental health services via existing MCH and HIV care programs, yet these efforts have not yet been implemented. Mental health screening is not currently standardized in antenatal and postnatal care visits in Kenya, representing a missed opportunity to offer mental health services to those with PMAD symptoms.

In a recent study among a large cohort of women in Western Kenya, our team found 25% had likely depression during pregnancy and 17% postpartum,^52^ yet few-to-none of these women would have been routinely screened per current Kenyan MCH guidelines. Among the same cohort of perinatal Kenyan women, we compared depressive symptom screening tools for a range of performance characteristics, finding that the PHQ-2 and PHQ-9 had acceptable properties supporting the mhGAP screening approach of having a non-specialized provider screen clients with two questions (like the PHQ-2/GAD-2), followed by screening by a more-specialized provider using a longer, validated scale (like the PHQ-9/GAD-7).^50^ Using mhGAP assessment by non-specialized providers can increase screening in MCH and PMTCT clinics with limited specialized workforce needs. However, **screening alone does not improve PMAD outcomes; screening needs to be followed by improved access to perinatal mental health treatments.**

**A7. Reducing perinatal morbidity and mortality in Africa—the region with the highest burden worldwide—will require integrated mental health services**. Despite a 41% global reduction in neonatal mortality over the last decade, preventable neonatal deaths continue at 2.5 million neonatal deaths per year, accounting for 47% of deaths among children under five.^53^ The burden is highest in SSA, where 27 neonates die within the first month of life among every 1,000 live births.^53^ Gains in neonatal survival have been slower than those seen in maternal and child survival overall—slowest in the highest burden regions, especially Africa.^54,55^ Conventional interventions to improve population-level neonatal indicators through promotion of attendance to routine MCH care have improved maternal and child health across SSA.^56,57^ Specific postnatal interventions such as treatment for infection and hemorrhage, and counselling on exclusive breastfeeding and birth spacing have reduced neonatal deaths.^56,58^ Yet achieving satisfactory neonatal survival remains elusive. Closing the “last mile” in neonatal health demands extension beyond conventional approaches, such as integrating depression screening into MCH services to increase treatment and reduce maternal depression-related neonatal outcomes.^4,15,55,59^

**A8. Improving access to perinatal mental health treatments requires attention to both the delivery framework and the intervention being delivered.** With regard to treatment delivery framework, only 20% of referred women with a positive PMAD screen typically access services following referral to specialty mental health provider.^60^ Barriers such as transportation, childcare, stigma and cost contribute to this low follow-through rate on referrals.^61^ Moreover, LMICs experience extreme shortages of specialist mental health providers:^62^ Kenya, for example, has 1 psychiatrist per 500,000 residents^63^ (compared to the recommended 1 per 30,000),^64^ making specialty models alone unrealistic. Additionally, women prefer to receive mental health treatments in their prenatal or medical setting where possible.^65^ Integrating treatments into MCH clinics can address multiple barriers, and is effective and cost effective.^66,67^ The WHO recommends that mental healthcare be integrated into MCH care and suggests providing interventions based on an individual’s needs, and at increasing intensity based on the severity of mental health symptoms. Recommended interventions include psychoeducation, psychotherapies, referral to specialty care, and antidepressants. However, **perinatal mental health interventions and delivery frameworks for women have not been rigorously evaluated in Kenya.**

Perinatal peer support programs can improve PMAD symptoms whether provided face-to-face, over phone or over the internet, presenting a crucial adjunct to specialty care. Interventions with ≥1 session per week that last ≤3 months have been shown to be particularly effective.^70^ Studies demonstrated that non-specialist providers can effectively deliver evidence-based psychological interventions, like culturally adapted cognitive behavioral interventions.^71^ ^70,72^ However, these effectiveness studies failed to evaluate implementation outcomes that would offer policymakers the data required to ensure sustainment and scale-up.

With regard to PMAD interventions women prefer psychotherapy to medication for mild-to-moderate depression, especially during pregnancy.^65,73^ **Problem Management Plus (PM+)** is an evidence-based low-intensity psychological intervention endorsed by WHO for adults experiencing adversity. It is a manualized 5-session program based on Cognitive Behavior Therapy (CBT) **and is feasible for delivery by lay providers**. It is effective in the treatment of depression, anxiety and stress. PM+ is an ideal psychological intervention in the MCH and PMTCT context given its brevity (as compared to Problem Solving Therapy which includes 8-15 sessions), its focus on psychosocial problems, which are central to the experience of perinatal women,^74^ and feasibility for delivery by providers without mental health training. However not all women will respond to PM+ and those with severe or persistent symptoms will require additional treatment – either psychotherapy or pharmacotherapy.

**A9. Stepped care interventions can ensure treatment for women with symptoms of any severity.** Lay providers may not be able to support patients with more severe depressive or anxiety symptoms requiring referral to specialty mental health for assessment and treatment. However, treatment intensification is often lacking in programs that aim to integrate mental health care into routine MCH settings. A systematic review of such programs found that very few programs included availability of pharmacotherapy or referral to specialty mental health when needed.^75^ **There is a need for evaluation of stepped care integrated mental health models within MCH and PMTCT that include peer-delivered interventions and intensification of care (both psychotherapy and pharmacotherapy) using the expertise of psychiatrists and psychologists where needed within a multidisciplinary team**.^75^

**A10. In the context of mental health workforce shortages in LMICs, telemedicine platforms have the potential to bring specialty expertise where needed.** The onset of the COVID-19 pandemic accelerated implementation of telepsychiatry in different settings, including perinatal integrated care models.^76^ Recent research suggests telemedicine is appropriate and feasible in treating psychiatric disorders in Kenya,^77^ and there are clear guidelines and recommendations for integrating telemedicine into health systems in Africa.^78^ There is a need to study how telemedicine can be incorporated into a stepped care model for mental health treatment integrated into a MCH program. **A stepped care treatment model which deploys peer-delivered PM+ followed by tele-consultation from a psychiatrist (as required) has the potential to change the way PMAD is detected and treated among women, efficiently utilize scarce mental healthcare resources, and improve clinical outcomes for mothers and babies.**

**A11. In addition to clinical effectiveness, it is essential to ensure this stepped care intervention has optimal implementation.** Implementation determinants – barriers and facilitators to implementation – can be overcome or enhanced using implementation strategies – specific approaches aimed at enhancing implementation. Simultaneously testing the clinical effectiveness of an intervention alongside measuring implementation outcomes and testing implementation strategies is an efficient Hybrid II design,^79^ which can accelerate the rate at which evidence-based interventions reach populations. To enable Kenyan policymakers to make informed decisions about a new stepped care approach to PMAD in MCH and PMTCT, it is important to evaluate service delivery and implementation outcomes, and test implementation strategies that are essential for delivery of the intervention at scale.

In order to deliver the multi-component stepped care intervention, certain implementation strategies are essential and should be bundled together. Training and supervisory strategies are needed to ensure fidelity and sustainment of new service delivery models by providing peer, MCH, and PMTCT providers with the education and support necessary to build capacity and confidence. Technology-enabled collaborative learning is being used more frequently in health care organizations to support provider education, including in Kenya,^80^ is equivalent to face-to-face education, and can increase participants’ knowledge and knowledge integration.^81^. Tele-mentoring may also improve provider reported satisfaction, confidence, self-efficacy and behaviors in identifying and managing conditions that are within their scope of practice but in which they lack training.^82^ When these tele-mentoring sessions have a multidisciplinary composition, the effects on learning can be greater than that seen with one-on-one teaching.^83^ Beyond essential implementation strategies, additional implementation strategies may enhance service delivery and require careful selection to match critical barriers.

**A12. Tailoring evidence-based interventions will be needed to successfully deliver them in routine care.** An expansive evidence base supports stepped care mental health interventions as a promising approach to expanding access to mental health services. However, implementing such an approach in a new setting, like Kenya, requires careful adaptation of the model to suit the context.^84^ Participatory design methods are increasingly used in behavioral and mental health research to engage stakeholders (e.g., policymakers, HCWs, and community members) in defining intervention design and adaptation. **Co-design principles applied to adapting intervention components and selecting implementation strategies with relevant players enables high-quality, scalable, and sustainable delivery of a complex intervention.**

Overall, evidence suggests that a stepped care intervention for PMAD treatment integrated into MCH and PMTCT services has high potential to positively impact the health and wellbeing of mother-infant pairs. To our knowledge, no study has formally evaluated effectiveness of a stepped care approach to alleviating PMAD while assessing implementation outcomes that could ensure future expansion and sustainment among women in an LMIC setting.

# **9) RATIONALE**

Our approach to **integrate mental health services in MCH in Kenya** is novel, potentially scalable and aligns with the Kenyan National initiatives and mental health policy goals. Integrating **a multi-component stepped care intervention that includes universal mhGAP screening, peer-delivered PM+ counselling, and tele-psychiatry into routine MCH care** has the potential to address a critical gap in mental health among perinatal women in low- and middle-income countries.

**Engaging integral stakeholders in participatory design of an integrated intervention and its implementation strategies is an innovative approach to adapting and optimizing IPMH for the Kenyan context.** Including end users (those delivering and receiving the intervention) in adapting interventions and designing implementation strategies enhances feasibility and acceptability and increases the likelihood of widespread adoption. While participatory design approaches have been used in developing digital mental health intervention, this is the first application, to our knowledge, of the participatory design approach to an integrated mental health treatment in MCH.

**Implementing a stepped care approach that includes peer-led psychotherapy and in-facility tele-psychiatry in a multidisciplinary team integrated into MCH and PMTCT is unique.** Integration of mental healthcare in primary care settings with care provision by non-specialist providers and engagement of lay workers has been endorsed by WHO and Kenya’s national mental health strategy. The proposed IPMH strategy utilizes trained HTS providers and other lay providers who are already an integral part of the perinatal team to screen perinatal women for depression and offer PM+ under the supervision and support of the clinical team hence mitigating against the severe shortage of specialized mental healthcare providers in Kenya. In-facility tele-linkage with mental health specialists for perinatal women who have severe depression, anxiety or suicidal ideation is novel and will ensure increased access to specialized mental health care for those who need it the most without straining the overburdened mental health care delivery system.

**Utilizing multidisciplinary tele-mentoring sessions for shared training and professional development will optimize delivery of IPMH.** These sessions will provide a platform for specialists to share expertise, advice, and clinical mentoring; and for sites to learn from each other as they implement screening and treatment for depression among perinatal women. The integrated multidisciplinary discussions will thus be sustainable beyond the project time and can be scaled to other facilities and disciplines. This “training” implementation strategy is unique as it will bring together diverse members of the clinical team, including HTS providers, mentor mothers, lay providers, nurses, primary care physicians, and psychiatrists on one platform for bidirectional learning.

**Evaluating implementation science outcomes of IPMH will provide rigorous data that will facilitate sustainment and scale-up**, including on its effectiveness, acceptability, penetration, equity of care provision, and cost that will facilitate sustainment and scale-up.  This will be among the first studies to adapt evidence based stepped care model, PM+ and telepsychiatry linkage in Kenya and which in addition incorporates teleconferenced didactic and case discussions.

# **10) HYPOTHESIS, OBJECTIVES, & STUDY QUESTIONS:**

## **10.1) BROAD OBJECTIVES**

This proposed study aims to evaluate the effect of IPMH, a stepped care intervention for screening and treatment of PMAD among perinatal women, on clinical and implementation outcomes.

## **10.2) SPECIFIC OBJECTIVES**

**Aim 1:** **Using participatory design, optimize and adapt IPMH and develop implementation strategies.**

1. **IPMH intervention components will be optimized** and **adapted**, guided by client, provider and policymaker stakeholder input to ensure that IPMH can be implemented in these facilities. We will conduct quantitative surveys, key informant interviews (KIIs) and focus group discussions (FGDs), followed by workshops with stakeholders to co-design the intervention.
2. **Implementation strategies** **will be developed** to address stakeholders’ perceived barriers to implementing IPMH using the Consolidated Framework for Implementation Research (CFIR). We will conduct KIIs and FGDs with providers and policymakers to identify barriers, followed by workshops with stakeholders to co-design strategies to overcome them.

**Hypothesis:** Implementation barriers include insufficient provider knowledge and skills in counselling techniques, overburdened staff, and inefficient patient flow. We predict that promising implementation strategies will include provider training, structured supervision, continuous quality improvement, multidisciplinary teleconferenced team-based learning, streamlining service delivery points, provision of job aides and manuals and audit and feedback in the first 6 months of implementation.

**Aim 2:** **Determine the effect of IPMH and implementation strategies on mental health, HIV care, and pregnancy outcomes among perinatal women from pregnancy to 6 months postpartum.**

We will conduct a 1:1 cluster-randomized trial in 20 facilities in Western Kenya among pregnant women (2^nd^ trimester) with PMAD symptoms (Patient Health Questionnaire-2 [PHQ-2]≥3 or Generalized Anxiety Disorder-7 [GAD-7]≥3). Control facilities will provide enhanced standard of care (PMAD screening followed by standard of care). Intervention facilities will provide IPMH. Effectiveness outcomes will be compared between study arms. Primary outcomes include PMAD symptoms (PHQ-9 score and GAD-7 score) at 6 months postpartum. Secondary outcomes include quality of life and adverse pregnancy outcomes. Exploratory outcomes include HIV viral suppression, MTCT, and HIV-related stigma.

**Hypothesis:** Perinatal women at facilities randomized to IPMH will have lower depressive and anxiety symptoms, lower adverse pregnancy outcomes, and improved quality of life compared with standard of care.

**Aim 3: Determine effect of IPMH and its implementation strategies on service delivery and implementation outcomes, and identify multilevel drivers of successful implementation.**

**a)** Guided by Proctor’s Implementation Outcomes Framework (IOF), we will quantitatively compare penetration, efficiency and equity of screening and care provision. Additionally, acceptability, adoption, fidelity, and cost of IPMH will be described within the intervention arm.

**b)** Guided by the Consolidated Framework for Implementation Research (CFIR), we will explore intervention-, facility- and provider-level drivers of implementation success, by conducting quantitative and qualitative analysis in facilities with high vs. low penetration, equity, efficiency, acceptability, and fidelity.

**Hypothesis:** IPMH will increase penetration and equity of care provision to perinatal women experiencing PMAD without negatively impacting efficiency of care delivery.

# **11) STUDY DESIGN AND METHODOLOGY**

## **11.1) STUDY AREA DESCRIPTION:**

The study will be conducted in 20 health care facilities in Kisumu, Siaya, and Homa Bay counties of Western Kenya, the region with the highest HIV prevalence. Facilities will be selected based on HIV sero-prevalence (15-20%) and ANC volume (≥20 new ANC clients per month), and will include a diversity of facility characteristics such as rural/urban location, mental healthcare model, and facility level.

## **11.2) STUDY DESIGN OVERVIEW**

Aim 1 is a qualitative aim using participatory design to optimize and adapt the study intervention and develop implementation strategies. Aim 2 & 3 comprise a Hybrid Type II Trial which includes a cluster-randomized controlled trial to evaluate the clinical outcomes (Aim 2) and implementation science outcomes (Aim 3) of the IPMH study. This goal will be achieved by (1) Stakeholder input, refining the IPMH intervention and developing implementation strategies to facilitate its integration into MCH and PMTCT services in Kenya, (2) evaluating the intervention’s effect on perinatal mental health and perinatal, and (3) evaluating the effect of implementation strategies on service delivery and implementation outcomes (penetration, efficiency, equity, acceptability, adoption, fidelity, and cost) and identifying drivers of implementation success

**
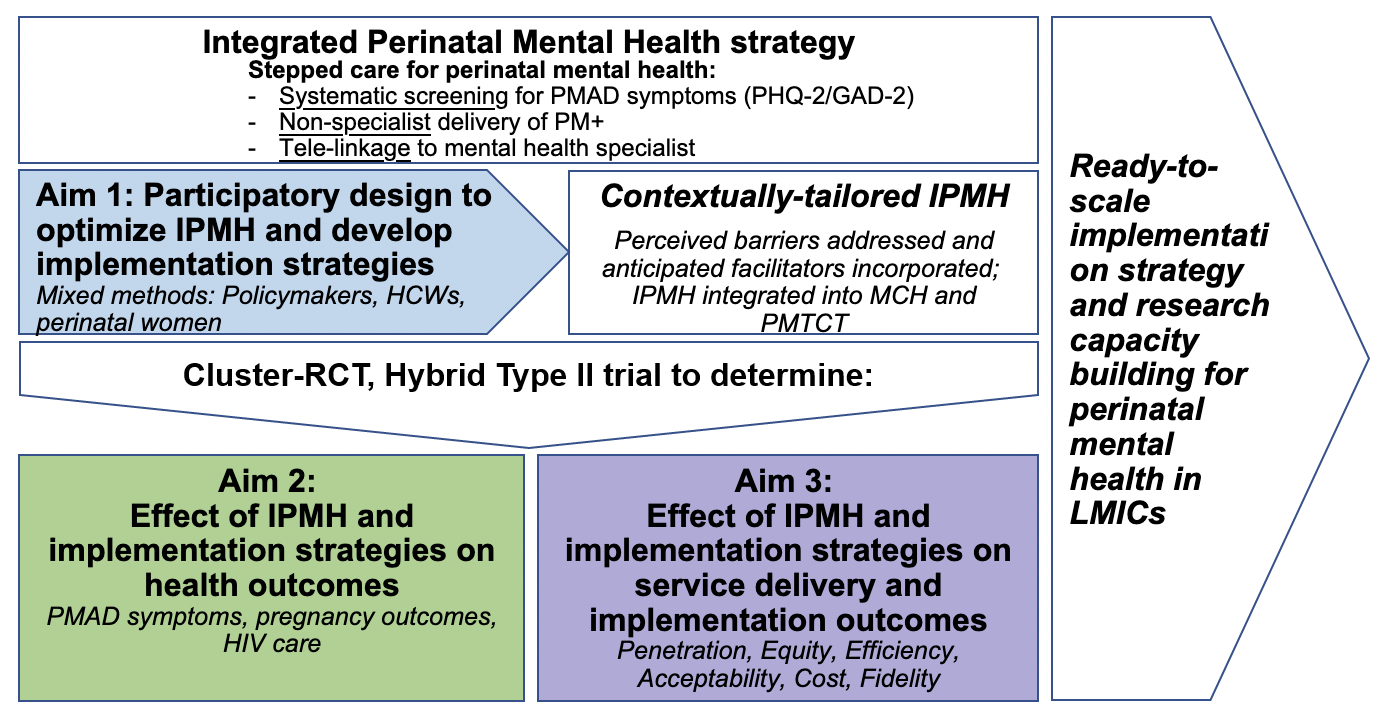
Figure 1: Study Overview**

## **AIM 1: Using participatory design, optimize IPMH and develop implementation strategies.**

### **STUDY DESIGN**

This will be a sequential mixed methods aim, including quantitative surveys followed by qualitative KIIs and FGDs. Surveys will answer questions related to the frequency of delivered services and associated barriers; qualitative data will probe to answer questions related to the depth of barriers and engage in brainstorming solutions. Data collection activities will gather current mental health services offered and stakeholder input on intervention components (Aim 1a) and implementation strategies (Aim 1b). Data will be used to co-design intervention components and implementation strategies with stakeholders. Outputs of Aim 1 will be materials for IPMH delivery such as standard operating procedures, job aides,

patient flow pathways, and training manuals.

### **STUDY POPULATION AND SAMPLE SIZE DETERMINATION**

We will engage several stakeholder groups: perinatal women, specialist and non-specialist HCWs (HTS and lay providers), and policymakers working in perinatal and mental health at the national and county levels. Aim 1a will be addressed using data from all groups, while Aim 1b will use data from policymakers and HCWs, since they are best suited to comment on systems- and HCW-level strategies needed to implement IPMH. Participant numbers, inclusion criteria, and outcomes are summarized in Sample sizes were selected to enable sufficient precision (quantitative) or to reach theoretical saturation of themes (qualitative).

| **Table 3. Aim 1 participant characteristics and data collection activities** | | | | | |
| --- | --- | --- | --- | --- | --- |
| **Population** | **Policymakers** | **HCWs** | | **Perinatal Women** | |
| **Data collection** | - KIIs | - Quantitative survey | - FGDs | - Quantitative survey | - FGDs |
| **Sample size** | - 30 - 10 specialists | - 180 non-specialists - 20 specialists | - 40 non-specialists | - 400 | - 40 |
| **Inclusion criteria** | - Age ≥18 - Involved in perinatal & mental health policy at national or county level | - Age ≥18 - Nurse, clinical officer, or lay providers at study facility; - clinical psychologist or psychiatrist at county or national referral hospital | | - Age ≥14 - Receiving perinatal care at study facility - Pregnant or <1 year postpartum | |
| **Aim 1a Outcomes** | - Surveys: Current mental health services offered, experienced barriers and facilitators - FGDs, IDIs: Design recommendations for perinatal mental health care | | | | |
| **Aim 1b Outcomes** | - FGDs, IDIs: Anticipated barriers & facilitators to IPMH implementation; recommended implementation strategies | | | N/A | |

A subset of 12 participants from FGDs and KIIs across all stakeholder groups will be invited to join a participatory design group (PDG). This will be a compensated group of policymakers, HCWs, HTS providers, lay providers and perinatal women who will be invited to participate in a series of 4-6 workshops aimed at analyzing the summary of survey, KII and FGD data and adapting IPMH and defining its implementation strategies.

### **RECRUITMENT, ENROLMENT AND DATA COLLECTION**

Potential participants will be approached by study staff and referred to the study research assistant at each clinic to obtain additional information and provide informed consent if interested. The study team will provide information about the study and facility staff will be encouraged to contact the study if they are interested. Policymakers will be individually recruited based on existing contact with the study team, email contacts from government directory searches, or network referral. We will conduct cross-sectional surveys among HCWs and perinatal women to characterize current mental health services, barriers and facilitators among a broad sample of participants. We aim for a statistically representative sample of participants for this quantitative survey; based on past studies conducted by this team, we expect low refusal rates. We will then conduct individual semi-structured KIIs with purposively selected policymakers and FGDs with purposively selected HCWs and perinatal women for in-depth exploration. Each FGD will include 6-12 participants, large enough to stimulate discussion but not so large to hinder participation. FGDs and KIIs will be facilitated in participants’ preferred language(s) by a Kenyan qualitative interviewer fluent in English, Swahili and Luo, the most common languages in the area. FGDs and KIIs will be audio-recorded, transcribed verbatim, and translated into English. Discussion guides will first enumerate experienced barriers and facilitators to mental healthcare implementation, and key design questions to optimize IPMH. Then they will elicit and prioritize implementation strategies drawing from the CFIR and published literature on barriers and implementation strategies

### **DATA ANALYSIS**

Quantitative data will be summarized descriptively, focusing on the frequency and diversity of service delivery. Qualitative transcripts will be analyzed by a team of at least two analysts, who will develop a codebook using a combination of deductive and inductive methods. Deductive codes will be drawn from the CFIR pre-developed codebook and unique constructs presented in the recent task sharing review, and inductive coding in which emergent themes from the transcripts are added. Analysts will independently code transcripts using the Dedoose or Atlas software, meet to resolve coding discrepancies, and synthesize themes to create a concept map. In addition to preparation for academic publication, findings of quantitative and qualitative data collection will be summarized in a format that is accessible to stakeholders, to guide PDG workshops.

## **AIM 2: Determine the effect of IPMH and implementation strategies on mental health and pregnancy outcomes among perinatal women from pregnancy to 6 months postpartum.**

### **STUDY DESIGN**

We will conduct a 1:1 cluster randomized controlled trial (RCT) in 20 facilities in Western Kenya to determine the effect of IPMH (optimized in Aim 1) on clinical outcomes. All steps of IPMH will be delivered by facility-based staff (not study staff) to align with real-world conditions and evaluate implementation outcomes (Aim 3). Facilities will be randomized to receive IPMH and associated implementation strategies vs. the SOC. To identify potential study participants across both study arms, HTS providers at all study sites will screen clients for PMAD symptoms using the PHQ-2 and GAD-2, offering potential study enrollment to pregnant women with PMAD symptoms (PHQ-2≥3 and/or GAD-2≥3). To determine the effect of IPMH and its implementation strategies, we will compare outcomes between facilities randomized to receive IPMH versus the SOC.

### **STUDY POPULATION AND SAMPLE SIZE DETERMINATION**

The IPMH study is designed to identify pregnant women who would benefit from mental health support; thus women are eligible for inclusion in the study if they are:

- Pregnant and ≥20 weeks gestation
- Attending ANC care at the facility
- ≥14 years old
- screen positive for PMAD symptoms (PHQ-2≥3 and/or GAD-2≥3).

In Kenya, pregnant women age ≥14 are considered emancipated minors and can consent to study participation independently. Participants on antidepressants will be eligible if they have been on a stable dose for ≥6 months. Among attending ANC, we anticipate 15% will screen positive for PMAD symptoms (PHQ-2≥3 and/or GAD-2≥3) and will be offered enrollment.

Table shows minimum detectable differences between groups based on expected frequencies, with 10 clusters per arm,10% attrition, α=0.05, 80% power, a conservative coefficient of variation (0.25), and 2-sided tests. Effect sizes of 0.4 for psychological outcomes are clinically meaningful

| **Indicator** | **N (cohort)** | **Outcome (control)** | **Outcome (intervention)** | | **Absolute difference** | **Effect size*** |
| --- | --- | --- | --- | --- | --- | --- |
| ***Primary*** | | | | | | |
| PHQ-9 score at 6mo  (Mean [SD]) | 2970(Total) | 10.5 [5.0] | 8.84 [5.0] | | 1.66 | 0.33 |
|  |  | 9.5 [5.0] | 7.84 [5.0] | |  |  |
| GAD-7 score at 6mo  (Mean [SD]) | 2970(Total) | 10 [5.0] | 8.34 [5.0] | |  |  |
|  |  | 9 [5.0] | 7.34 [5.0] | |  |  |
| ***Secondary*** | | | | | | |
| PHQ-9 score at 6mo  (Mean [SD]) | 405(WLWH) | 10.5 [5.0] | 8.35 [5.0] | | 2.15 | 0.43 |
|  |  | 9.5 [5.0] | 7.35 [5.0] | |  |  |
| GAD-7 score at 6mo  (Mean [SD]) | 405(WLWH) | 10 [5.0] | 7.85 [5.0] | |  |  |
|  |  | 9 [5.0] | 6.85 [5.0] | |  |  |
| WHOQOL BREF score at 6mo  (Mean [SD]) | 2970(Total) | 3.4 [0.5] | 3.565 [0.5] | | 0.165 | 0.33 |
|  | 405(WLWH) | 3.4 [0.5] | 3.62 [0.5] | | 0.22 | 0.44 |
| Any adverse perinatal outcome at 6 wks (%) | 2970(Total) | 35.0% | 23.5% | | 11.5% | 0.67 |
|  | 405(WLWH) | 35.0% | 18.6% | | 16.4% | 0.53 |
| *Effect size: Cohen’s d (continuous variable) or Relative Risk (categorical variable) | | | |  |  |  |

### **
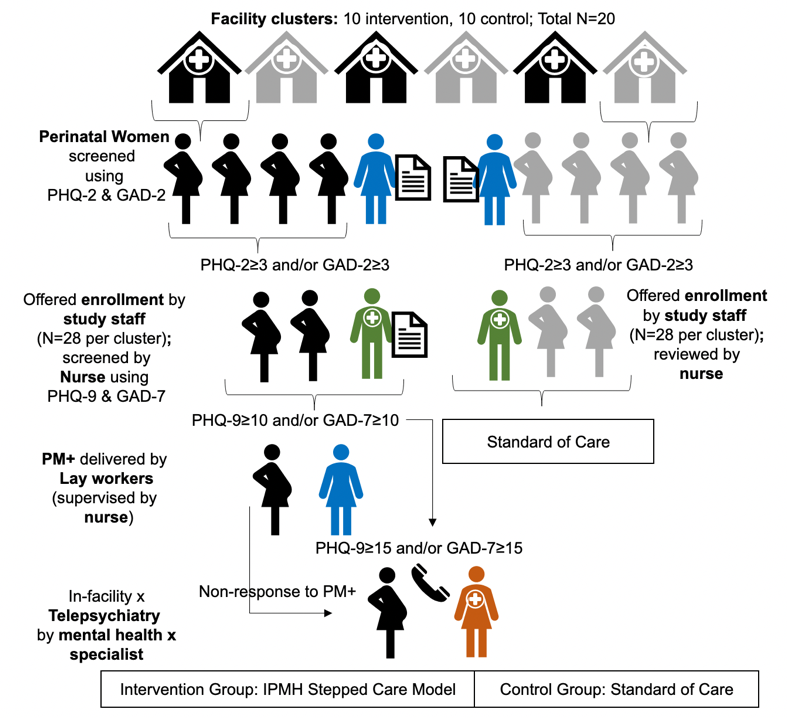
RECRUITMENT AND ENROLMENT**

**Figure 2: Schematic of IPMH RCT design**

Pregnant women seeking ANC at each of the 20 RCT facilities will be recruited for study participation through in-person outreach by the study team at the facilities. Potential participants will be approached by MCH clinic staff and referred to the study research assistant at each clinic to obtain additional information and provide informed consent if interested. Study staff will assess eligibility with a tablet-based screening questionnaire. Any woman at high risk of self-harm based on a study self-harm assessment protocol, has cognitive impairments, or psychotic symptoms will not be eligible to participate, but will be directly assessed by a supervising clinician who will determine next steps in referral and treatment. We will enroll ≥28 participants per facility. All perinatal women will be screened for PMAD symptoms by lay workers, using the PHQ-2 and GAD-2. Positive screeners (PHQ-2≥3 and/or GAD-2≥3) will be requested to provide informed consent. After providing informed consent, data will be collected by study staff through tablet-based questionnaires recording demographics, maternal and infant medical history and conditions, care seeking history, and maternal social and mental health, and perinatal care history. At enrollment, telephone numbers and addresses will also be collected for participants and 1-2 other people with separate addresses who can be contacted in the event that the participant cannot be reached. Women who opt not to provide secondary contact information will still be eligible for study participation in an effort to improve study generalizability. Participants will then be referred to nurses to administer additional screening by PHQ-9 and GAD-7. Those with likely depression (PHQ-9≥10) or anxiety (GAD-7≥10) will be offered PM+, delivered weekly by lay provider, under the supervision of MCH nurses (Figure 2).

Women with severe depressive symptoms (PHQ-9≥15), those who endorse suicidality, or non-responders to PM+ will be provided mental healthcare via in-facility tele-linkage to a psychiatrist based at a regional or national referral hospital. Nonresponse is defined as <50% reduction or <5-point decrease in PHQ-9 or GAD-7 score after conclusion of PM+ sessions, ascertained by a MCH nurse. Regardless of PHQ-9 score, those who endorse PHQ-9 item 9 (suicidal ideation) will be assessed further using our study self-harm protocol and those considered high risk will be withdrawn from the study and assessed by a supervising clinician. Those deemed appropriate to continue in the study will receive in-facility tele-linkage to a psychiatrist. The psychiatrist will make recommendations regarding medication management prescribed by facility provider or referral to a psychologist for tele-linked psychotherapy for 10-12 cognitive behavioral therapy sessions.

PM+: PM+ is a brief psychotherapy endorsed by WHO for delivery by non-specialists in LMICs for treatment of depression, anxiety, and stress (Figure 3). It consists of 2 assessment sessions and 5 weekly 90-minute sessions that cover 4 topics: managing stress, managing problems, increasing activity, and strengthening social support. The mechanism of action is development of psychosocial skills measured by the Reducing Tension Checklist. PM+ builds ability to manage emotional distress and address psychological problems (stress, fear, feelings of helplessness) and practical problems (livelihood problems, conflicts, etc.).
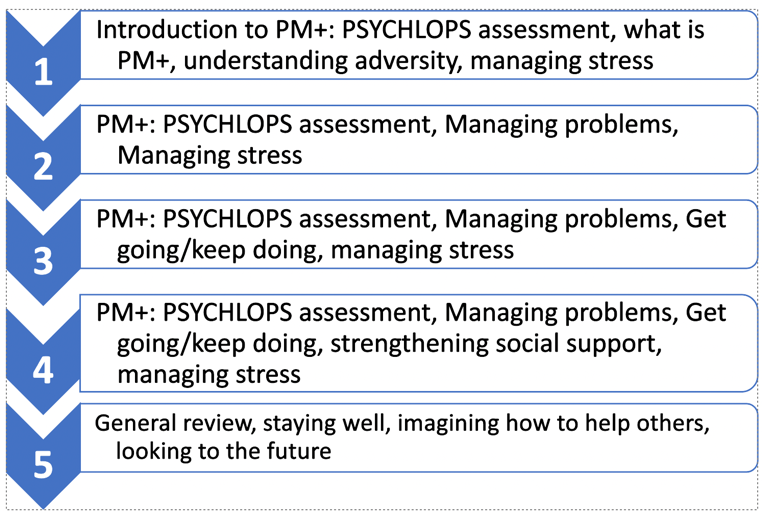
 PM+ is available in Kiswahili, has been delivered by community health workers in Kenya, and its dissemination is supported by Kenya MOH. In intervention sites, we will train lay workers to administer PM+ and nurses to supervise and assess treatment response.

**Figure 3: PM+ overview**

**In-facility tele-psychiatry:** Perinatal women who screen positive for severe depression (PHQ-9≥15), severe anxiety (GAD-7≥15) or who fail to respond to PM+ (<50% reduction or <5-point decrease in PHQ-9 or GAD-7 score after conclusion of PM+ sessions) will be offered in-facility tele-psychiatry services (we expect ~8% of participants; unpublished data). Tele-psychiatry services will be provided on a secure Zoom platform, which is currently used in Kenya. Intervention facilities will have consultation rooms with a tablet with cellular internet to facilitate telepsychiatry sessions, scheduled by the facility nurse. Tele-psychiatry services will be provided by psychiatrists and psychologists engaged by the study. The facility nurse will document the care plan for the patient.

**Control:** Enhanced standard of care: eSOC includes two enhancements: (1) HTS providers and lay workers will conduct screening for PMAD symptoms (PHQ-2 and GAD-2). (2) The study team will provide a PMAD referral information sheet to all facilities describing inpatient and outpatient psychiatry services at the nearby referral hospitals (JOORTH and Kisumu County Hospital).

### **RANDOMIZATION**

Ten facilities will be randomized to IPMH, 10 to eSOC. We will use restricted randomization to ensure balance of confounding factors, such as: HIV prevalence, client volume, and distance to regional referral hospital (Jaramogi Oginga Odinga Teaching and Referral Hospital [JOORTH]).

### **DATA COLLECTION**

Study visits will be conducted by study staff using tablet-based questionnaires at enrollment (pregnancy), 6 weeks, 14 weeks, and 6 months postpartum among participants in both study arms to align with routine well-baby visits. Questionnaires will be used to record self-reported outcomes and sociodemographic and clinical characteristics that may be associated with them, such as maternal education, household income, employment, parity, distance from home to clinic, intimate partner violence, food insecurity, prior healthcare experiences, and infant sex. The exit visit questionnaire will also evaluate participants’ experiences and satisfaction with the care received. Data will also be abstracted from patient facility records and the MCH booklet for pregnancy outcomes. Participants who are 2 weeks late for a study visit will be actively traced by phone call and/or home visit to optimize completeness of visit data. This approach has been highly acceptable in our other cohorts of peripartum women in Western Kenya and has resulted in excellent retention.

## **AIM 3: Determine effect of IPMH and its implementation strategies on service delivery and implementation outcomes, and identify multilevel drivers of successful implementation.**

### **STUDY DESIGN**

Drawing from the IOF and CFIR frameworks, we will employ quantitative and qualitative methods to assess service delivery and implementation outcomes of IPMH (penetration, efficiency, equity, acceptability, adoption, fidelity, cost) and multilevel drivers of implementation success.

## **Aim 3a: Determine service delivery and implementation outcomes.**

Alongside clinical outcomes (Aim 2), we will evaluate service delivery and implementation outcomes at the 20 study facilities during the implementation period (Figure 4). We hypothesize that, compared with the eSOC, IPMH will improve: penetration of any mental healthcare (defined as the proportion of clients receiving screening and treatment) and equity of mental healthcare (defined as demographic characteristics of clients receiving screening and treatment). We also hypothesize that efficiency of regular clinical care will not be reduced by integration of IPMH into perinatal care (defined as perinatal visit completion, healthcare worker workload, and client wait times). To test these hypotheses, these outcomes will be compared between the intervention and control arms. In the intervention arm, we hypothesize that IPMH will have high acceptability to both perinatal women and HCWs (based on self-report), will be implemented with high fidelity (based on the number of PM+ sessions, appropriate referral for tele-psychiatry, and provider competence in IPMH delivery), and will have affordable cost (based on cost estimates from provider and client perspectives).

| Table C7: Summary of service delivery and implementation outcome indicators, data sources, and analyses  ME, mixed effects; RE, random effect | | | | | |
| --- | --- | --- | --- | --- | --- |
| Outcome | **Definition** | **Indicator** | **Data source** | **Timepoint** | **Analysis** |
| *Compare control vs intervention arms* | | | | | |
| Penetration | Extent to which MCH and PMTCT clients receive screening & treatment for PMAD | - Proportion of clients receiving recommended screening (PHQ-9 and GAD-7) - Proportion of positive screeners linking to any mental healthcare | - Perinatal women self-report questionnaires - Perinatal women self-report questionnaires | Cross-sectionally per client throughout study | ME binomial regression (RE by facility) |
| Equity | Absence of avoidable differences in care delivery between groups of people | - Disparities in accessing:   - screening - any mental healthcare between quintiles of demographic characteristics (e.g., age, income, distance to facility) | - Perinatal women self-report questionnaires | Cross-sectionally per client throughout study | ME linear or binomial regression (RE by facility) |
| Efficiency | Minimize intervention and implementation strategy inputs for number of perinatal women receiving care. | - Average number of provider hours per month per perinatal woman - Average number of hours perinatal women wait time per month - Average incremental cost per perinatal women screened & treated (see cost) | - Time & motion study; direct observation of patient flow and provider time - Routine program data abstraction | - RCT launch - 6 months post-launch - 12 months post-launch | ME linear regression (RE by facility) |
| *Evaluate within intervention arm* | | | | | |
| Acceptability | Perception that IPMH is agreeable or satisfactory | - Acceptability of Intervention Measure (AIM) score (Perinatal & HCWs)^78^ - Qualitative assessment | - HCW & perinatal women questionnaires & IDIs | - RCT launch - 6 months post-launch - 12 months post-launch | - Descriptive quantitative & qualitative - Quantitative determinants by ME linear regression (RE by facility, RE by perinatal women/HCW respondent for acceptability & fidelity) - Qualitative determinants by thematic analysis |
| Cost | Incremental costs to health system & client from IPMH implementation | -Average incremental total facility cost by arm  -Average incremental unit cost per facility by arm, where unit costs are defined as incremental cost per client screened or per client screened and treated  -Average incremental cost per percent reduction in postpartum depression | - Activity-based costing - Time and motion of provider time and non-personnel resource use - HCW & perinatal women questionnaire, IDI or FGD - Project and MOH facility expense reports | - RCT launch - 12 months post-launch |  |
| Fidelity | The extent to which IPMH is implemented as designed | - Proportion of positive screeners receiving 5 sessions of PM+ - Proportion of positive screeners receiving all PM+ manual components - Proportion of PM+ non-responders receiving tele-linkage - Lay provider competence in PM+ following initial training & during implementation | - Perinatal women report of PM+ services delivered - Self-administered HCW competence checklist based on content of training | - 6 months post-launch - 12 months post-launch |  |

### **STUDY POPULATION**

Aim 3a involves data collection from 2970 perinatal women and 200 healthcare workers working at the intervention and control facilities. Eligibility criteria are:

Perinatal women:

• Pregnant and ≥20 weeks gestation

• Age ≥14 years

• Screen positive for PMAD symptoms (PHQ-2≥3 and/or GAD-2≥3)

• Willing to return to the MCH and PMTCT clinic for study visits

Healthcare workers:

• Age ≥18

• Nurse, clinical officer, HTS provider or lay healthcare worker at study facility, or clinical psychologist or psychiatrist at county or national referral hospital

Perinatal women, HTS providers, lay workers, facility nurses, and psychology/psychiatry specialists at study facilities will be recruited by study staff for participation in questionnaires assessing CFIR determinants. Perinatal women will participate in questionnaires at each study visit and study close. Healthcare providers will participate in questionnaires at study launch, 6 months and 12 months after study launch. No active efforts will be made to retain providers in their professional positions. However, telephone numbers will be collected for providers at enrollment. If a provider misses a study visit but is confirmed to still be in their professional role, study staff will trace the provider by phone.

### **DATA COLLECTION**

Service delivery and implementation outcomes will be determined from several data sources:

**RCT questionnaires:** Self-report questionnaires will be administered to RCT participants at study visits to ascertain IPMH acceptability, receipt of screening, linkage to care, and PM+ components received. They will be administered to RCT participants at study visits (see Aim 2). These will ascertain receipt of screening by PHQ-9 and linkage to care for depression or anxiety, including HTS/ lay HCW delivered PM+, specialist-delivered psychiatric care, or antidepressant description. Participants reporting receipt of IPMH will be asked which topics they discussed. Additionally, participants’ RCT exit questionnaire will include standardized questions assessing IPMH acceptability

**Checklists:** We will assess HCW competence directly after training using a self-administered checklist.

**Routine program data:** We will abstract anonymous patient count data on a number of MCH and PMTCT appointments completed per month from registers at all facilities.

**RCT HCW questionnaires:** All HCWs will complete a questionnaire at RCT launch, 6 months and 12 months after launch to assess IPMH acceptability and HCW time use.

**Time and motion study and direct observation of patient flow:** This will be conducted to determine the time and resources necessary to provide IPMH compared to eSOC. Time and motion studies will be conducted over a two-week period at each site at RCT launch and 12 months later. Trained research assistants will conduct direct observation of patient flow (physical walk throughs to graphically represent patient flow pathways) and time-and-motion data collection (counting minutes spent waiting and receiving services).

**Costing:** Measures of costs will include (a) clinical and health system costs and (b) participant cost. We will use a combination of methods for collecting data using project expenditures and micro-costing methods to estimate the incremental economic costs in each arm in a subset of 5-10 facilities, purposively selected to represent heterogeneity in drivers of cost. We will collect site-specific inputs for labor and non-labor costs related to planning, training, facilitation administration, and IPMH service delivery. For perinatal women, we will estimate the value of their time as well as costs incurred for childcare and lost wages. We will estimate start-up and recurrent costs for all activities.

Where available, we will obtain expenditure data directly from project records or facility financial records to ensure that we capture all costs associated with planning, program design, adaptation, materials development, training, and clinical service delivery. For resource use not captured in project expense reports, we will conduct interviews with IPMH providers, perinatal women, and administrators on the resource use for each intervention component and implementation strategy, capturing key information on quantities and prices each activity, as possible. At the facility level, we will collect site-specific inputs for labor (personnel time, volunteer labor) and non-labor costs (commodities, capital goods (e.g., vehicles, computers, cell phones, other equipment, overhead) related to planning, training, facilitation administration, and IPMH service delivery. All goods and services will be valued regardless of if they were paid for by the project or donated. For perinatal women, we will estimate the value of their time traveling to and from facilities and receiving care services, as well as costs incurred for childcare and lost wages. To estimate a total incremental cost and unit cost, we will estimate the cost of IPMH plus implementation strategies compared to eSOC in control facilities, along with the number of participants receiving care in each arm. We will conduct costing activities in a subset of 5-10 facilities purposively selected to represent heterogeneity in drivers of cost (e.g., patient volume, staffing, location, prevalence of PMAD symptoms). We will estimate start-up and recurrent costs for all intervention and relevant implementation strategies following best practices.

## **Aim 3b: Identify multilevel drivers of implementation success.**

Determinants of implementation outcomes as guided by the CFIR, will include positive and negative determinants of penetration, equity, efficiency, acceptability, and fidelity of IPMH (Figure 4 above).

### **STUDY POPULATION**

We will recruit 2970 perinatal women and 100 healthcare workers working at the intervention facilities. Eligibility criteria are:

Perinatal women:

• Pregnant and ≥20 weeks gestation

• Age ≥14 years

• Screen positive for PMAD symptoms (PHQ-2≥3 and/or GAD-2≥3)

• Willing to return to the MCH clinic for study visits

Healthcare workers:

• Age ≥18

• Nurse, clinical officer, HTS provider, lay healthcare worker at study facility, or clinical psychologist or psychiatrist at county or national referral hospital

We will recruit perinatal women participants, and HCWs (HTS providers, lay HCWs, facility nurses, and psychology/psychiatry specialists) in intervention facilities to participate in in-depth interviews, focus group discussions, and key informant interviews about overall experience, acceptability, fidelity, and cost of the intervention.

### **DATA COLLECTION**

**RCT for perinatal women & HCW questionnaires:** We will administer standardized questionnaires to perinatal women and HCW during the RCT assessing CFIR determinants quantitatively and use mixed effects regression to identify the drivers associated with high vs low penetration, efficiency, equity, acceptability, and fidelity. The study is not specifically powered to detect changes in this analysis of exploratory determinants.

**Qualitative interviews with perinatal women and HCWs:** IDIs, FGDs and KIIs will be conducted among perinatal women and HCWs in intervention facilities. We will assess constructs from CFIR using contextually adapted questions. We will analyze transcripts as described in Aim 1. Qualitative and quantitative data will be combined in a convergent mixed methods approach for triangulation.

### **OUTCOMES**

### **AIM 1**

Anticipated implementation strategies: Selection of the bundle of strategies to optimize IPMH delivery will be based on data collected in Aim 1b. Table 4 summarizes barriers we anticipate based on the literature and the team’s experience, as well as implementation strategies that may be effective to overcome them, based on published literature in similar contexts. Data from KIIs and FGDs will be used to identify 3 bundles of strategies based on feasibility, alignment with theory, and impact (effect size) of strategies in literature. The PDG will additionally specify each implementation strategy per Proctor’s strategy specification guidelines (including actor, action, action target, dose, temporality, implementation outcome targeted, and theoretical justification). These tables will form the basis of training materials and SOPs to be used in Aim 2, and a training curriculum to be shared with MOH for later use.

| **Table 4. Anticipated implementation barriers & strategies** | |
| --- | --- |
| **Barrier** | **Implementation strategy** |
| Lack of staff training in counseling | - Provide initial & refresher training - Develop job aides and manuals - Multidisciplinary learning collaborative - Audit and feedback - Supportive supervision |
| Inefficient patient flow | - Streamlining service delivery points - Fast tracking |
| Lack of stakeholder buy-in | - Targeted meetings |
| Overburdened HCWs | - Task shifting/sharing - Client self-delivered steps |

###

### **AIM 2**

**Primary outcomes:** We will ascertain PMAD symptoms as PHQ-9 scores and GAD-7 scores collected by study staff, compared between arms at 6 months postpartum. We will compare continuous PHQ-9 and GAD-7 scores between the study arms at 6 months postpartum using generalized estimating equations (GEE) with exchangeable correlation, Gaussian family, and identity link, clustered by facility.

**Secondary outcomes:** We will compare self-reported quality of life (using the WHOQOL BREF instrument) at 6 months postpartum and any adverse pregnancy outcome (per self-report and clinic record validation) at 6 weeks postpartum (composite outcome defined as pregnancy loss, stillbirth, preterm birth, low birthweight, small for gestational age, neonatal hospital admission, or neonatal death) between arms using generalized estimating equations (GEE) with exchangeable correlation, Gaussian family, and identity link, clustered by facility. To confirm the hypothesized mechanism of action of the intervention, we will evaluate the 10-item Reducing Tensions Checklist which measures the use of behavioral and psychosocial coping skills related to PM+.

**Exploratory outcomes:** To evaluate the impact of IPMH on HIV-related outcomes, we will compare HIV viral suppression (viral load<200c/ml), HIV MTCT, self-reported ART adherence (using the Wilson 3-item scale76) and HIV-related stigma (using the HASI-P77) between study arms at 6 months postpartum.

Primary analyses will be Intent-To-Treat. To determine if randomization was balanced, we will compare baseline characteristics between study arms using Chi-squared (categorical variables) and Kruskal-Wallis tests (continuous) between arms. Primary analyses will be adjusted for imbalanced baseline factors

| **Outcome** | **Indicator** | **Source** | **Timing of Analysis*** |
| --- | --- | --- | --- |
| PMAD symptoms | Patient Health Questionnaire-9 (PHQ-9) score | Questionnaire | 6 months postpartum |
|  | Generalized Anxiety Disorder-7 (GAD-7) score | Questionnaire | 6 months postpartum |
| Quality of life | WHOQOL BREF score | Questionnaire | 6 months postpartum |
| Any adverse perinatal outcome | Any of the following: Pregnancy loss stillbirth, preterm birth, low birthweight, intrauterine growth restriction, neonatal hospital admission, and neonatal death | Questionnaire, Medical records, MCH booklet | 6 weeks postpartum |
| Mechanism of action for PM+ | Reducing Tension Checklist | Questionnaire | 6 weeks postpartum |
| HIV viral suppression | Proportion of WLWH with HIV viral load <200 | Questionnaire, Medical records | 6 months postpartum |
| Mother-to-child transmission of HIV | Proportion of mother-infant pairs with HIV MTCT | Questionnaire, Medical records | 6 months postpartum |
| ART adherence | Proportion of WLWH who took 95% of ART doses | Questionnaire, Medical records | 6 months postpartum |
| HIV-related stigma | Proportion of WLWH reporting any HIV stigma | Questionnaire | 6 months postpartum |

### **AIM 3**

Aim 3a: We will compare penetration, equity and efficiency between arms. All data will be correlated at the level of facility, so arms will be compared by mixed effects (ME) regression with random effect for facility. Acceptability, cost, and fidelity will be presented descriptively within the intervention arm.

Aim 3b: We will use mixed effects regression to identify the drivers associated with high vs low penetration, efficiency, equity, acceptability, and fidelity from RCT perinatal women & HCW questionnaires.

# **12) TRAINING PROCEDURES**

Dr. John Kinuthia will supervise training of clinical personnel and study staff in study procedures.

# **13) QUALITY ASSURANCE PROCEDURES**

Clinical Care: the study will adhere to Government of Kenya (GoK) guidelines for the care of pregnant/postpartum women and their infant. Data collected as part of the study will be abstracted from the mother’s “Mother and Child Health booklet” and patient file, as well as the MCH and CCC clinic’s medical records, where possible, in addition to questionnaires administered to participants.

Adherence to protocol: Weekly reporting of enrollment, follow-up, medical complications, laboratory results and specimen collection will enable us to monitor that the study is running according to approved protocols. Frequent reporting will also enable us to respond quickly to any problems that arise during the study.

Data Quality: A dedicated data team will be responsible for ensuring quality data collection using an electronic data collection platform. The data team will communicate weekly with the operations team and leadership including reporting on data cleaning, study monitoring, and interim analyses.

# **14) ETHICAL CONSIDERATIONS**

*COVID precautions for all visits*

We will follow local Kenyan guidelines regarding COVID-19 precautions. *If social distancing guidelines due to the COVID-19 global pandemic prevent in-person administration of questionnaires, these will be conducted via telephone, whenever possible.*

*Compensation:*

All study participants will receive Ksh 500 at all study visits to compensate for their time and transportation expenses to participate in the study.

*Participant Retention:*

Study staff will make every reasonable effort to retain participants in follow-up. This will include tracing phone calls or home visits as needed. Study procedures will be conducted at these visits as appropriate.

*Participant Withdrawal:*

Participants may voluntarily withdraw from the study for any reason at any time.

*Future contact with subjects:*

Contact information will be limited to our study team. We may contact participants to confirm data collected during the course of the study, as issues may be identified during data analysis once the participant had exited. We will also retain contact information to inform participants about future studies. If participants do not wish to be re-contacted we will not do so.

## **14.1 ASSESSMENT OF RISKS AND BENEFITS**

### **POTENTIAL RISKS**

We do not anticipate any physical risks due to this study. Women participating in the RCT may experience psychological distress due to interviews about sensitive mental health experiences. Additionally, there may be a breach in confidentiality of participants. There is also a risk of breach of confidentiality and disclosure of information to persons other than the participant. Healthcare professionals are at risk of inducement by their employer to respond to questions in favourable ways.

### **PROTECTION AGAINST RISK**

Human subjects training: We will train study staff on the importance of confidentiality and ensure they receive human subjects training.

Confidentiality: Risk of breach of confidentiality of study data is low, as study data will not contain identifiers, and the link between study data and identifiable information will be protected. Additionally, all questionnaire and SMS data will be stored on secure, password-protected servers and accessed on password-protected devices. Study staff will be trained in the importance of confidentiality during human subjects training prior to study implementation. Throughout the consent process we will emphasize the potential risk associated with breach of confidentiality. All in-person data collection (questionnaire and interviews) will be conducted in a private area.

Psychological distress: The study team is supported by clinicians experienced in care of peripartum women and neonates, who will assist with training study staff in the management of emotional distress and mental illness, and referral to in-person services.

Risk of inducement and disciplinary action: The informed consent process for provider FGDs and IDIs will stress that participation is voluntary, the purpose of the data collection is to evaluate and improve the IPMH intervention, not punish poor worker performance, and their employment will not be affected by their responses. All study participants may opt-out of any study procedure or refuse to answer any question at any time.

### **POTENTIAL BENEFITS OF PROPOSED RESEARCH TO THE SUBJECTS AND OTHERS**

**Direct:** All participants will be screened for PMAD using the PHQ2 tool and provided with psychosocial support regardless of the PHQ2 score. Participants in the intervention arm will receive PM+ and, if necessary, telepsychiatry, which are both evidence-based interventions to alleviate depression and anxiety symptoms. There are also possible benefits from regular interactions with the healthcare workers to answer mental health-related questions and concerns. Infants will be provided with in-depth assessment of growth. All participants are hypothesized to benefit more than persons attending other clinics in the region not participating in the study.

**Care for persons with severe depression and/or suicidal ideation:** Women with severe depressive symptoms (PHQ-9≥15), those who endorse suicidality, or non-responders to PM+ will be provided mental healthcare via in-facility tele-linkage to a psychiatrist l. Non-response is defined as <50% reduction or <5-point decrease in PHQ-9 score after conclusion of PM+ sessions, ascertained by a MCH or PMTCT nurse. Regardless of PHQ-9 score, those who endorse PHQ-9 item 9 (suicidal ideation) will be assessed further using our study self-harm protocol and those considered high risk will be withdrawn from the study and assessed by a supervising clinician. Those deemed appropriate to continue in the study will receive in-facility tele-linkage to a psychiatrist. The psychiatrist will make recommendations regarding medication management or referral to a psychologist for tele-linked psychotherapy where 10-12 cognitive behavioral therapy sessions will be delivered. If medication is recommended, the facility physician will prescribe and the psychiatrist will provide up to four telemedicine follow-ups to ensure adequate dose, assess for side effects and monitor response.

**Benefits to the community:** An important goal of this study is to achieve the study objectives and get results that can be translated into policy briefs and guidelines in the management of PMAD among pregnant and postpartum women in the community. This can then be cascaded to all pregnant and postpartum women. This will benefit the community beyond the proposed study timeline.

### **IMPORTANCE OF THE KNOWLEDGE GAINED**

Knowledge gained from the proposed study will aid in closing the PMAD screening and treatment gap experienced by pregnant and postpartum women which can be cascaded to all perinatal women. It will highlight the barriers, facilitators and provide a feasible, sustainable and scalable strategy to provide care for women with PMAD within LMICs. This has not been previously studied in Kenya hence the knowledge gained will guide the ANC and mental health guidelines and policies in Kenya. Moreover, it will have substantial impact on regional and global programs for screening and treating PMAD among perinatal women.

# **15)** **DATA MANAGEMENT AND SHARING PLAN**

## **ELEMENT 1: DATA TYPE**

1. **Types and amount of scientific data expected to be generated in the project:**

This proposal will generate data from approximately 3800 participants:

1. 630 participants (healthcare workers and perinatal clients) in formative data collection (Aim 1)
2. 2970 participants (perinatal clients) in the longitudinal RCT cohort (Aim 2)
3. 200 participants (healthcare workers) providing care in the RCT (Aim 3)

Data include quantitative questionnaire data from all participant groups, transcripts of key informant interviews (KIIs) and focus group discussions (FGDs) from Aims 1 and 3 participants. Additionally, we will conduct costing activities that will generate time, resource and cost estimates for intervention delivery.

1. **Scientific data that will be preserved and shared, and the rationale for doing so:**

All study materials will be stored in a password-protected folder on a secure server maintained by the Kenyatta National Hospital (KNH) with back-up at the end of each day. All datasets will be subject to data standards such as documentation of methods of collection through codebooks, SOPs for data collection, and limitations associated with data collection. Data containing participant identifiers will not be shared, but will be preserved for six years after termination of the study, per institutional policy. Questionnaire data will be collected and stored electronically in HIPAA-compliant, secure, password-protected encrypted REDCap databases. These databases will be managed by the PIs and study coordinator.

A de-identified dataset will also be created to share publicly for dissemination and publication. The final versions of any de-identified data collected and/or generated will be made publicly available within 12 months of publication. Final data gathered from this project will be archived for data sharing purposes in Dryad, NIH’s recommended open-access generalist data repository. As an open access repository, Dryad only takes data with no personally identifiable information and deposits must be made open access to the public. No special software is needed to submit materials. In addition, the repository software creates persistent URLs that will not change over time.

1. **Metadata, other relevant data, and associated documentation:**

The study protocols, sample informed consent/assent forms, data collection instruments (including questionnaires or interview guides, as appropriate), and data dictionaries will be made accessible in data repositories where data are shared. Each variable in the data dictionaries will include a brief description of the item, question text, variable name/label, value name/label, and codes for missing values (including unknown, not reported, not applicable, or refusal). Documentation will be provided in PDF, Word, or Excel format. Deposit of materials to Dryad requires a minimum set of descriptive information (metadata) to be provided at the point of deposit. Some of this metadata will be automatically generated by the software used by Dryad; other pieces including author, title, and date, will be provided by the depositor.

## **ELEMENT 2: RELATED TOOLS, SOFTWARE AND/OR CODE**

For the study use, questionnaire data will be collected in and accessed from the REDCap databases. Data entered into REDCap will be de-identified. These data will be analyzed using R statistical software and de-identified data and analysis files developed during the study will be stored in the KNH server and only accessible to investigators from KNH and UW. Focus group transcripts, time and motion data, and cost estimates will be stored as text documents and stored in the UW’s OneDrive. No specialized tools, software, and/or code are needed to access or manipulate shared scientific data. In addition to sharing study data, statistical analysis code will be made available as part of publications, by posting it in GitHub.

## **ELEMENT 3: STANDARDS**

The FAIR Guiding Principles will be applied to the scientific data generated by this study: the data will be findable, accessible, interoperable, and reusable. The data will be assigned a persistent unique identifier and indexed in a searchable repository. An authentication and authorization procedure will be put in place to make the data accessible. Data will be shared in an interoperable format to allow for use by third parties. Finally, the data will be well-described through data dictionaries, codebooks, and reference lists to allow for replication in different settings. Dryad integrates with any SWORD-compliant repository.

## **ELEMENT 4: DATA PRESERVATION, ACCESS, AND ASSOCIATED TIMELINES**

1. **Repository where scientific data and metadata will be archived:**

Scientific data arising from the supplement will be archived in Dryad within 12 months of publication date. Dryad is one of NIH’s recommended open-access generalist repositories and UW is a member of Dryad. Select study data and information will be made available to the research community free of charge.

1. **How scientific data will be findable and identifiable:**

Cleaned finalized data will be findable and identifiable using a persistent unique identifier (“digital object identifier”) assigned to the dataset by Dryad. Dryad also uses persistent unique identifiers for depositing authors (ORCID) and funders (Funder Registry).

1. **When and how long the scientific data will be made available:**

Cleaned de-identified data will be made available to other users within 12 months of an associated publication. Data deposited in Dryad is intended to remain permanently archived and available.

## **ELEMENT 5: ACCESS, DISTRIBUTION, OR REUSE CONSIDERATIONS**

1. **Factors affecting subsequent access, distribution, or reuse of scientific data:**

De-identified and cleaned data will be shared as allowed by the participant’s informed consent, PI approval, and the institutional certification, following associated publication.

1. **Whether access to scientific data will be controlled:**

De-identified data deposited in Dryad will be openly available to the public under the terms of a Creative Commons Zero (CC0) waiver.

1. **Protections for privacy, rights, and confidentiality of human research participants:**

Throughout and following data collection procedures, all participant identifiers will be stored in a HIPAA-compliant, secure, password-protected encrypted REDCap database separate from the main study databases. Identifying information can only be accessed by study staff approved by the PIs. Informed consent documents completed at enrollment into the cohorts or during other data collection activities such as prior to focus group discussions and time and motion activities include explicit language about storage of data for use in future research and sharing of de-identified data. Participants will not be contacted or re-consented for future sharing or accessing data through repositories. Only de-identified data will be archived and available for sharing.

## **ELEMENT 6: OVERSIGHT OF DATA MANAGEMENT AND SHARING**

Monitoring of and compliance with this Data Management and Sharing Plan will be the responsibility of the PIs and study team. The PIs will provide oversight and ensure appropriate, ethical, data management adherence to KNH and UW standards. Issues related to confidentiality, security, and intellectual property will adhere to guidelines set forth by KNH and UW.

**Roles and Responsibilities**

A Data Safety and Monitoring Board (DSMB) will be convened to provide external monitoring of participant safety and study data collection. The DSMB will be comprised of experts in biostatistics, mental health in resource limited settings, HIV/AIDS outcomes, perinatal health outcomes, stepped care designs, and the conduct and analysis of cluster-randomized controlled trials. The DSMB will also include an ex officio member from NIMH. The main responsibilities of DSMB will be, but not limited to, the following:

- Review of protocols, consent procedures, consent forms, and safety plans prior to initiation of the study;
- Monitoring of the progress of the study, including recruitment and retention of participants, adverse events (AEs), serious adverse events (SAEs), reasons for participant withdrawal, adherence to the timeline of the study, quality of data, and protocol violations;

**Frequency of Monitoring**

The DSMB will meet at the initiation of the study, every 6 months during active recruitment, and at study end. The DSMB will review study protocols, analysis plans, progress, retention, and AEs/SAEs. During the first meeting, the DSMB will evaluate the analysis plan and determine stopping rules for benefit, futility, or harm using O’Brien-Fleming stopping boundaries. The DSMB will review data from one interim analysis, to be conducted when approximately half of the total person-time has been accrued. At this interim analysis, the DSMB will determine whether stopping rules have been met. The study statistician (Dr. Barbra Richardson) will oversee preparation of interim analyses and presentation to the DSMB.

**Clinical Trials Registration**

Dr. Ronen will be responsible for registering and providing updated information about the clinical trial on ClinicalTrials.gov.

# **16) STUDY LIMITATIONS AND HOW TO MINIMIZE THEM:**

During direct observation, such as that employed for time-and-motion data collection, there is the possibility of the Hawthorne effect, in which individuals modify their behavior because they are observed. This is typically a concern with shorter data collection periods; these data will be collected over a series of approximately 2 weeks, so we anticipate a more limited or negligible Hawthorne effect. There is no potential benefit for the individuals being observed to modify their practices since the evaluation of is of the health system in general rather than a specific behavior.

# **17) DISSEMINATION PLAN**

The investigators are committed to the open and timely dissemination of study outcomes which are important for guiding policy and practice. Established Community Advisory Boards (CABs) will include providers and community members living in the areas to whom results will be disseminated and who will give guidance if community issues arise. The findings will be shared with the participating facilities as presentations. Written reports and Policy briefs will be developed for sharing with the Kenya Ministry of Health at the conclusion of the trial. In addition the research team will organize two workshops in Kenya with policy makers, mental health providers, early career investigators and the community advisory board. The first workshop will deliberate on findings of the Integrated Perinatal Mental Health program developed in aim 1. The second workshop will be used to disseminate results of the cluster randomized RCT (aim 2). Participants will also deliberate on the service delivery and implementation outcomes, and identify drivers of successful implementation of the IPMH (aim 3). A policy brief will be developed from the discussion of the workshops highlighting areas that require further research and/or proposing strategies to optimize integration of mental health services in perinatal care. Finally findings will be disseminated to the research community in the form of conference presentations and journal articles.

# **18) TIMELINE/ TIME FRAME:**

|  | Year 1 | | | | Year 2 | | | | Year 3 | | | | Year 4 | | | | Year 5 | | | |
| --- | --- | --- | --- | --- | --- | --- | --- | --- | --- | --- | --- | --- | --- | --- | --- | --- | --- | --- | --- | --- |
|  | Q1 | Q2 | Q3 | Q4 | Q1 | Q2 | Q3 | Q4 | Q1 | Q2 | Q3 | Q4 | Q1 | Q2 | Q3 | Q4 | Q1 | Q2 | Q3 | Q4 |
| Ethical Approvals |  |  |  |  |  |  |  |  |  |  |  |  |  |  |  |  |  |  |  |  |
| CRFS, SOPS, Database preparation |  |  |  |  |  |  |  |  |  |  |  |  |  |  |  |  |  |  |  |  |
| **Aim 1: Using participatory design, optimize and adapt IPMH and develop implementation strategies.** |  |  |  |  |  |  |  |  |  |  |  |  |  |  |  |  |  |  |  |  |
| - Quantitative surveys, KIIs and FGDs |  |  |  |  |  |  |  |  |  |  |  |  |  |  |  |  |  |  |  |  |
| - Participatory Design Group |  |  |  |  |  |  |  |  |  |  |  |  |  |  |  |  |  |  |  |  |
| **Aim 2:**  **Determine effect of IPMH on mental health, HIV care, and pregnancy outcomes among perinatal women from pregnancy to 6 months postpartum.** |  |  |  |  |  |  |  |  |  |  |  |  |  |  |  |  |  |  |  |  |
| - Training study staff |  |  |  |  |  |  |  |  |  |  |  |  |  |  |  |  |  |  |  |  |
| - Participant recruitment and follow-up |  |  |  |  |  |  |  |  |  |  |  |  |  |  |  |  |  |  |  |  |
| **Aim 3: Determine effect of IPMH and its implementation strategies on service delivery and implementation outcomes, and identify multilevel drivers of successful implementation.** |  |  |  |  |  |  |  |  |  |  |  |  |  |  |  |  |  |  |  |  |
| - Perinatal women self-report questionnaires |  |  |  |  |  |  |  |  |  |  |  |  |  |  |  |  |  |  |  |  |
| - Time and motion study |  |  |  |  |  |  |  |  |  |  |  |  |  |  |  |  |  |  |  |  |
| - Post-RCT qualitative interviews |  |  |  |  |  |  |  |  |  |  |  |  |  |  |  |  |  |  |  |  |
| Data verification and cleaning |  |  |  |  |  |  |  |  |  |  |  |  |  |  |  |  |  |  |  |  |
| Data analysis |  |  |  |  |  |  |  |  |  |  |  |  |  |  |  |  |  |  |  |  |
| Development and dissemination of policy briefs to the Kenyan MOH |  |  |  |  |  |  |  |  |  |  |  |  |  |  |  |  |  |  |  |  |
| Facility and community dissemination of study results |  |  |  |  |  |  |  |  |  |  |  |  |  |  |  |  |  |  |  |  |
| Manuscript preparation |  |  |  |  |  |  |  |  |  |  |  |  |  |  |  |  |  |  |  |  |

# **19) HUMAN SUBJECTS**

1. Protection of Human Subjects

Procedures for human subject’s protection in this research will follow guidelines and policies of the Kenyatta National Hospital Ethical Review Committee (ERC) and the University of Washington Institutional Review Board (IRB), in addition to NIH policies. All researchers and research staff will complete appropriate CITI modules, including local Kenya staff. Prior to initiating research, IRB approval will be completed with the Kenyatta National Hospital ERC and the University of Washington IRB. We will conduct face-to-face study training to review all human subjects procedures. This will include training on the study protocol, study consents, recruitment and enrollment procedures. All study materials will be reviewed and approved by both the UW IRB and the KNH ERC.

1. **Collaborating sites**

The study will be conducted in collaboration with the UW, KNH, HomaBay, Siaya and Kisumu counties. The study will be reviewed by the KNH ERC and UW IRB and will not be started before approvals are obtained from all two organizational review boards. The study will be conducted at 20 clinics in Kisumu County, in collaboration with Kenyatta National Hospital. University of Washington and Kenyatta National Hospital study investigators will be responsible for implementation of study procedures and overseeing and managing study staff. The study PI and co-Is will have access to deidentified study data stripped of names and phone numbers. The PI and co-Is will conduct site visits to research sites; however, only facility-based study staff members will have direct contact with study participants. Facility-based study staff will be responsible for obtaining consent from interested participants.

1. **Informed Consent**

Electronic written informed consent will be obtained for all study activities. All participants who agree to participate in study activities will sign the informed consent form using the electronic consent module through REDCap. This will allow for electronic collection of consent signatures, allowing for real time consent quality monitoring, as well as secure, encrypted, storage. Participants will receive a hardcopy of the electronically signed consent documents.

- RCT participants: Participants interested in study participation will be referred to study staff, who will provide detailed information about the types of data to be collected at the study visits, the intervention, potential risks and benefits of participation, and answer any participant questions. Informed consent will occur during one-on-one counseling sessions with study staff in a private clinic room, and will be conducted in English, Kiswahili, or Luo depending on the preference of the potential participant. Written consent materials will be translated into both Swahili and Luo. Women will be told that their clinical care will continue to be provided by facility (not study) staff regardless of their participation in the RCT. They will also be informed that they can withdraw from the study at any time. Pregnant adolescents age 14-17 are considered emancipated minors by Kenya law, and are able to legally provide their own consent without parental permission for participation in research.
- Healthcare workers and policymakers: Study staff will conduct informed consent with study nurses. Written materials will be shared, as well as a description of the content of the interview.

1. **Handling Adverse events**

Adverse events will be ascertained during follow-up visits, by phone, or home visit, using standardized questionnaires. Additionally, adverse events may be spontaneously reported to the study team. Adverse events include illness of participants or their infants, miscarriage, and reports of suicidal ideation. Serious adverse events (SAE) will include deaths, admissions to hospital, and prolongation of hospitalization. All events will be reported in writing, graded by the study clinicians and reviewed by the PI within 24 hours of the study learning of the event. Weekly summaries of adverse events will be reviewed by the study investigators. All adverse events will be recorded, including those unlikely to be related to the intervention. SAEs will be reported to the Institutional Review Boards in Nairobi and Seattle within 48 hours of detection and summarized in the annual NIH progress report. Any SAEs related to study participation and Unanticipated Problems will be reported to the NIMH PO within 10 business days of the team becoming aware. Blinded summaries of AEs and SAEs will be reviewed at DSMB meetings. The DSMB will determine if there are any safety issues that require unblinding of DSMB members in order to determine whether alteration or cessation of the study is necessary. In the event of an adverse event, including mental health crisis, participants will have an emergency contact number to phone or text. If we learn about adverse events we will refer women to the standard facility services. Through our prior studies in Western Kenya we have developed a directory of resources available at the study facilities and surrounding area, and SOPs to ensure women and babies who experience illness, food insecurity, intimate partner violence, depression or suicidality are referred to available resources.

# **20) REFERENCES**

1 Feigin VL, Nichols E, Alam T, *et al.* Global, regional, and national burden of neurological disorders, 1990–2016: a systematic analysis for the Global Burden of Disease Study 2016. *Lancet Neurol* 2019; published online March. DOI:10.1016/S1474-4422(18)30499-X.

2 McKeown RE. The Epidemiologic Transition: Changing Patterns of Mortality and Population Dynamics. *Am J Lifestyle Med* 2009; **3**: 19S-26S.

3 Whiteford HA, Degenhardt L, Rehm J, *et al.* Global burden of disease attributable to mental and substance use disorders: findings from the Global Burden of Disease Study 2010. *Lancet* 2013; **382**: 1575–86.

4 Collins PY, Patel V, Joestl SS, *et al.* Grand challenges in global mental health. *Nature* 2011; **475**: 27–30.

5 Vigo D, Thornicroft G, Atun R. Estimating the true global burden of mental illness. *The Lancet Psychiatry* 2016; **3**: 171–8.

6 GBD 2017 Disease and Injury Incidence and Prevalence Collaborators SL, Abate D, Abate KH, *et al.* Global, regional, and national incidence, prevalence, and years lived with disability for 354 diseases and injuries for 195 countries and territories, 1990-2017: a systematic analysis for the Global Burden of Disease Study 2017. *Lancet (London, England)* 2018; **392**: 1789–858.

7 Depression: The Second Leading Cause of Disability Worldwide. https://www.healthline.com/health-news/mental-depression-a-leading-cause-of-global-disability-110513#1 (accessed Jan 21, 2019).

8 World Health Organisation (WHO). Gender disparities in mental health. https://www.who.int/mental_health/media/en/242.pdf?ua=1 (accessed Jan 19, 2019).

9 World Health Organization. Gender and Mental Health. 2002 http://www.who.int/gender/other_health/genderMH.pdf (accessed Nov 1, 2018).

10 Rahman A, Patel V, Maselko J, Kirkwood B. The neglected ‘m’ in MCH programmes - why mental health of mothers is important for child nutrition. *Trop Med Int Heal* 2008; **13**: 579–83.

11 World Health Organization. Maternal mental health. WHO. 2015. http://www.who.int/mental_health/maternal-child/maternal_mental_health/en/ (accessed Nov 1, 2018).

12 O’hara MW, Swain AM. Rates and risk of postpartum depression—a meta-analysis. *Int Rev Psychiatry* 1996; **8**: 37–54.

13 Howard LM, Molyneaux E, Dennis C-L, Rochat T, Stein A, Milgrom J. Non-psychotic mental disorders in the perinatal period. *Lancet (London, England)* 2014; **384**: 1775–88.

14 Meltzer-Brody S. New insights into perinatal depression: pathogenesis and treatment during pregnancy and postpartum. *Dialogues Clin Neurosci* 2011; **13**: 89–100.

15 Kohn R, Saxena S, Levav I, Saraceno B. The treatment gap in mental health care. *Bull World Health Organ* 2004; **82**: 858–66.

16 Reid V, Meadows-Oliver M. Postpartum Depression in Adolescent Mothers: An Integrative Review of the Literature. *J Pediatr Heal Care* 2007; **21**: 289–98.

17 World Health Organisation (WHO). Adolescent pregnancy. 2018. https://www.who.int/en/news-room/fact-sheets/detail/adolescent-pregnancy (accessed March 23, 2019).

18 Sines BE, Syed U, Wall S, Worley H. Postnatal Care : A Critical Opportunity to save mothers and newborns. 2006; : p.1.

19 World Health Organization. Every Newborn: an action plan to end preventable deaths. 2014 http://apps.who.int/iris/handle/10665/127938.

20 Fisher J, Cabral de Mello M, Patel V, *et al.* Prevalence and determinants of common perinatal mental disorders in women in low- and lower-middle-income countries: a systematic review. *Bull World Health Organ* 2012; **90**: 139-149H.

21 Parsons CE, Young KS, Rochat TJ, Kringelbach ML, Stein A. Postnatal depression and its effects on child development: a review of evidence from low- and middle-income countries. *Br Med Bull* 2012; **101**: 57–79.

22 World Health Organisation (WHO). Maternal mental health and child health and development in low and middle-income countries. Report of the WHO-UNFPA meeting held in Geneva Switzerland 30th Jan-1st Feb 2008. Geneva, Switzerland, 2008.

23 World Health Organization. World Health Organization Mental Health Atlas 2011. 2011 http://www.who.int/about/licensing/copyrig (accessed Nov 4, 2018).

24 Sankoh O, Sevalie S, Weston M. Mental health in Africa. *Lancet Glob Heal* 2018; **6**: e954–5.

25 Alderdice F, Newham J. Global maternal mental health: where you live matters. *J Reprod Infant Psychol* 2016; **34**. DOI:10.1080/02646838.2016.1126945.

26 World Fertility Patterns 2015. 2015 www.unpopulation.org. (accessed Jan 21, 2019).

27 Howard LM, Khalifeh H. Perinatal mental health: a review of progress and challenges. *World Psychiatry* 2020; **19**: 313–27.

28 Dennis CL, Falah-Hassani K, Shiri R. Prevalence of antenatal and postnatal anxiety: Systematic review and meta-analysis. *Br J Psychiatry* 2017; **210**: 315–23.

29 Woody CA, Ferrari AJ, Siskind DJ, Whiteford HA, Harris MG. A systematic review and meta-regression of the prevalence and incidence of perinatal depression. *J Affect Disord* 2017; **219**: 86–92.

30 Nielsen-Scott M, Fellmeth G, Opondo C, Alderdice F. Prevalence of perinatal anxiety in low- and middle-income countries: A systematic review and meta-analysis. *J Affect Disord* 2022; **306**: 71–9.

31 Dadi AF, Akalu TY, Baraki AG, Wolde HF. Epidemiology of postnatal depression and its associated factors in Africa: A systematic review and meta-analysis. PLoS One. 2020; **15**. DOI:10.1371/journal.pone.0231940.

32 Zhu QY, Huang DS, Lv J Da, Guan P, Bai XH. Prevalence of perinatal depression among HIV-positive women: a systematic review and meta-analysis. *BMC Psychiatry* 2019; **19**: 330.

33 Psaros C, Smit JA, Mosery N, *et al.* PMTCT Adherence in Pregnant South African Women: The Role of Depression, Social Support, Stigma, and Structural Barriers to Care. *Ann Behav Med* 2020; **54**: 626–36.

34 Wagner GJ, Ghosh-Dastidar B, Mukasa B, Linnemayr S. Changes in ART Adherence Relate to Changes in depression as Well! Evidence for the Bi-directional Longitudinal Relationship Between Depression and ART Adherence from a Prospective Study of HIV Clients in Uganda. *AIDS Behav* 2020; **24**: 1816–24.

35 Bonari L, Pinto N, Ahn E, Einarson A, Steiner M, Koren G. Perinatal risks of untreated depression during pregnancy. *Can J Psychiatry* 2004; **49**: 726–35.

36 Xiao PL, Zhou YB, Chen Y, *et al.* Association between maternal HIV infection and low birth weight and prematurity: A meta-analysis of cohort studies. *BMC Pregnancy Childbirth* 2015; **15**: 246.

37 Cummings EM, Davies PT. Maternal depression and child development. *J Child Psychol Psychiatry* 1994; **35**: 73–112.

38 Alder J, Fink N, Bitzer J, Hösli I, Holzgreve W. Depression and anxiety during pregnancy: A risk factor for obstetric, fetal and neonatal outcome? A critical review of the literature. *J Matern Neonatal Med* 2007; **20**: 189–209.

39 Grigoriadis S, VonderPorten EH, Mamisashvili L, *et al.* The Impact of Maternal Depression During Pregnancy on Perinatal Outcomes. *J Clin Psychiatry* 2013; **74**: e321–41.

40 Grote NK, Bridge JA, Gavin AR, Melville JL, Iyengar S, Katon WJ. A Meta-analysis of Depression During Pregnancy and the Risk of Preterm Birth, Low Birth Weight, and Intrauterine Growth Restriction. *Arch Gen Psychiatry* 2010; **67**: 1012.

41 Hodgkinson SC, Colantuoni E, Roberts D, Berg-Cross L, Belcher HME. Depressive symptoms and birth outcomes among pregnant teenagers. *J Pediatr Adolesc Gynecol* 2010; **23**: 16–22.

42 Moraes AN, Likwa RN, Nzala SH. A retrospective analysis of adverse obstetric and perinatal outcomes in adolescent pregnancy: the case of Luapula Province, Zambia. *Matern Heal Neonatol Perinatol* 2018; **4**: 20.

43 Ganchimeg T, Ota E, Morisaki N, *et al.* Pregnancy and childbirth outcomes among adolescent mothers: a World Health Organization multicountry study. *BJOG* 2014; **121**: 40–8.

44 Rahman A, Surkan PJ, Cayetano CE, Rwagatare P, Dickson KE. Grand Challenges: Integrating Maternal Mental Health into Maternal and Child Health Programmes. *PLoS Med* 2013; **10**: e1001442.

45 Adewuya AO, Ola BO, Aloba OO, Mapayi BM, Okeniyi JAO. Impact of postnatal depression on infants’ growth in Nigeria. *J Affect Disord* 2008; **108**: 191–3.

46 Stewart RC, Umar E, Kauye F, *et al.* Maternal common mental disorder and infant growth a cross-sectional study from Malawi. *Matern Child Nutr* 2008; **4**: 209–19.

47 Harpham T, Huttly S, De Silva MJ, Abramsky T. Maternal mental health and child nutritional status in four developing countries. *J Epidemiol Community Health* 2005; **59**: 1060–4.

48 Tomlinson M, Cooper PJ, Stein A, Swartz L, Molteno C. Post-partum depression and infant growth in a South African peri-urban settlement. *Child Care Health Dev* 2006; **32**: 81–6.

49 Surkan PJ, Kennedy CE, Hurley KM, Black MM. Maternal depression and early childhood growth in developing countries: systematic review and meta-analysis. *Bull World Health Organ* 2011; **89**: 608-615E.

50 World Health Organisation (WHO). mhGAP Intervention Guide for mental, neurological and substance use disorders in non-specialized health settings. 2010 www.who.int/mental_health/mhgap (accessed May 6, 2019).

51 Demographic and Health Survey - Kenya. 2014 www.DHSprogram.com. (accessed June 1, 2019).

52 Larsen A, Pintye J, Marwa MM, *et al.* Trajectories and predictors of perinatal depressive symptoms among Kenyan women: a prospective cohort study. *The Lancet Psychiatry* 2022; **9**: 555–64.

53 You D, Hug L, Ejdemyr S, Beise J, Idele P, UN IGME. Levels & Trends in Child Mortality. New York, NY, 2015 http://www.childmortality.org/files_v20/download/IGME Report 2015_9_3 LR Web.pdf.

54 Lawn J, Kerber K, Enweronu-Laryea C, Cousens S. 3.6 Million Neonatal Deaths-What Is Progressing and What Is Not? Semin. Perinatol. 2010; published online Oct 17. DOI:10.1053/j.semperi.2010.09.011 <http://dx.doi.org/10.1053/j.semperi.2010.09.011>.

55 Lawn JE, Blencowe H, Oza S, *et al.* Every Newborn: progress, priorities, and potential beyond survival. *Lancet (London, England)* 2014; **384**: 189–205.

56 Langlois É V, Miszkurka M, Zunzunegui MV, Ghaffar A, Ziegler D, Karp I. Systematic reviews Inequities in postnatal care in low-and middle-income countries: a systematic review and meta-analysis. *Bull World Heal Organ* 2015; **93**: 259–70.

57 Lawn J, Mongi P, Cousens S. Opportunities for Africa’s Newborns. https://www.who.int/pmnch/media/publications/aonsection_I.pdf (accessed Nov 1, 2018).

58 Singh A, Yadav A, Singh A. Utilization of postnatal care for newborns and its association with neonatal mortality in India: an analytical appraisal. *BMC Pregnancy Childbirth* 2012; **12**: 33.

59 Rahman A, Iqbal Z, Bunn J, Lovel H, Harrington R. Impact of Maternal Depression on Infant Nutritional Status and Illness. *Arch Gen Psychiatry* 2004; **61**: 946.

60 Byatt N, Simas TAM, Lundquist RS, Johnson J V., Ziedonis DM. Strategies for improving perinatal depression treatment in North American outpatient obstetric settings. *J Psychosom Obstet Gynaecol* 2012; **33**: 143–61.

61 King CR, Morgan SM, Firebaugh CM, *et al.* Postpartum Depression: Far Reaching Impact and the Role of Empowerment. *Open J Depress* 2021; **10**: 29–42.

62 Bruckner TA, Scheffler RM, Shen G, *et al.* The mental health workforce gap in low- and middle-income countries: a needs-based approach. *Bull World Health Organ* 2011; **89**: 184–94.

63 Global Health Workforce statistics database. https://www.who.int/data/gho/data/themes/topics/health-workforce (accessed July 18, 2022).

64 Mental Health Care Health Professional Shortage Areas (HPSAs) | KFF. https://www.kff.org/other/state-indicator/mental-health-care-health-professional-shortage-areas-hpsas/?currentTimeframe=0&sortModel=%7B%22colId%22:%22Location%22,%22sort%22:%22asc%22%7D (accessed July 18, 2022).

65 Goodman JH. Women’s attitudes, preferences, and perceived barriers to treatment for perinatal depression. *Birth* 2009; **36**: 60–9.

66 Honikman S, van Heyningen T, Field S, Baron E, Tomlinson M. Stepped Care for Maternal Mental Health: A Case Study of the Perinatal Mental Health Project in South Africa. *PLoS Med* 2012; **9**: e1001222.

67 Patel V, Belkin GS, Chockalingam A, Cooper J, Saxena S, Unützer J. Grand Challenges: Integrating Mental Health Services into Priority Health Care Platforms. *PLoS Med* 2013; **10**. DOI:10.1371/JOURNAL.PMED.1001448.

68 World Health Organization. Maternal mental health and child health and development in low and middle income countries: Report of the meeting held in Geneva, Switzerland. *World Heal Organ* 2008; : 1–34.

69 Fang Q, Lin L, Chen Q, *et al.* Effect of peer support intervention on perinatal depression: A meta-analysis. *Gen Hosp Psychiatry* 2022; **74**: 78–87.

70 Kenya Ministry of Health. National Guidelines for PMTCT Peer Education and Psychosocial Support in Kenya. 2012 http://guidelines.health.go.ke:8000/media/National_Guidelines__for__PMTCT_Peer_Education_and_Psychosocial_Support_in_Kenya_KMMP.pdf (accessed July 18, 2022).

71 Ibu JM, Mhlongo EM. The Mentor Mothers Program in the Department of Defense in Nigeria: An Evaluation of Healthcare Workers, Mentor Mothers, and Patients’ Experiences. *Healthc (Basel, Switzerland)* 2021; **9**. DOI:10.3390/HEALTHCARE9030328.

72 Futterman D, Shea J, Besser M, *et al.* Mamekhaya: a pilot study combining a cognitive-behavioral intervention and mentor mothers with PMTCT services in South Africa. *AIDS Care* 2010; **22**: 1093–100.

73 Cuijpers P, Koole SL, Van Dijke A, Roca M, Li J, Reynolds CF. Psychotherapy for subclinical depression: meta-analysis. *Br J Psychiatry* 2014; **205**: 268–74.

74 Ashaba S, Kaida A, Coleman JN, *et al.* Psychosocial challenges facing women living with HIV during the perinatal period in rural Uganda. *PLoS One* 2017; **12**. DOI:10.1371/JOURNAL.PONE.0176256.

75 Prom MC, Denduluri A, Philpotts LL, *et al.* A Systematic Review of Interventions That Integrate Perinatal Mental Health Care Into Routine Maternal Care in Low- and Middle-Income Countries. *Front psychiatry* 2022; **13**. DOI:10.3389/FPSYT.2022.859341.

76 Shore JH, Waugh M, Calderone J, *et al.* Evaluation of telepsychiatry-enabled perinatal integrated care. *Psychiatr Serv* 2020; **71**: 427–32.

77 Njenga F, Ongeri L, Nguithi A, *et al.* Integrating telepsychiatry services in a care setting in Kenya: a case report. *BJPsych Int* 2022; **19**. DOI:10.1192/BJI.2021.38.

78 Mbunge E, Muchemwa B, Batani J. Are we there yet? Unbundling the potential adoption and integration of telemedicine to improve virtual healthcare services in African health systems. *Sensors Int* 2022; **3**: 100152.

79 Curran GM, Bauer M, Mittman B, Pyne JM, Stetler C. Effectiveness-implementation Hybrid Designs: Combining Elements of Clinical Effectiveness and Implementation Research to Enhance Public Health Impact. *Med Care* 2012; **50**: 217.

80 Dreizler L, Grace ;, Wanjiku W. Tele-ECHO for Point-of-Care Ultrasound in Rural Kenya: A Feasibility Study. .

81 Chipps J, Brysiewicz P, Mars M. A systematic review of the effectiveness of videoconference-based tele-education for medical and nursing education. *Worldviews evidence-based Nurs* 2012; **9**: 78–87.

82 Cantor JC, Chakravarty S, Farnham J, Nova J, Ahmad S, Flory JH. Impact of a Provider Tele-mentoring Learning Model on the Care of Medicaid-enrolled Patients With Diabetes. *Med Care* 2022; **60**: 481–7.

83 Dascalu MI, Bodea CN, Lytras M, De Pablos PO, Burlacu A. Improving e-learning communities through optimal composition of multidisciplinary learning groups. *Comput Human Behav* 2014; **30**: 362–71.

84 Powell BJ, Beidas RS, Lewis CC, *et al.* Methods to Improve the Selection and Tailoring of Implementation Strategies. *J Behav Health Serv Res* 2017; **44**: 177–94.
